# Is detection of enteropathogens and human or animal faecal markers in the environment associated with subsequent child enteric infections and growth: an individual participant data meta-analysis

**DOI:** 10.1016/S2214-109X(23)00563-6

**Published:** 2024-02-14

**Authors:** Andrew Mertens, Benjamin F Arnold, Jade Benjamin-Chung, Alexandria B Boehm, Joe Brown, Drew Capone, Thomas Clasen, Erica R Fuhrmeister, Jessica A Grembi, David Holcomb, Jackie Knee, Laura H Kwong, Audrie Lin, Stephen P Luby, Rassul Nala, Kara Nelson, Sammy M Njenga, Clair Null, Amy J Pickering, Mahbubur Rahman, Heather E Reese, Lauren Steinbaum, Jill R Stewart, Ruwan Thilakaratne, Oliver Cumming, John M Colford, Ayse Ercumen

**Affiliations:** a Division of Epidemiology, University of California, Berkeley, CA, USA; b Division of Biostatistics, University of California, Berkeley, CA, USA; c Division of Environmental Health Sciences, University of California, Berkeley, CA, USA; d Francis I Proctor Foundation and Department of Ophthalmology, University of California, San Francisco, CA, USA; e Department of Epidemiology and Population Health, Stanford University, Stanford, CA, USA; f Department of Civil and Environmental Engineering, Stanford University, Stanford, CA, USA; g Department of Medicine, Stanford University, Stanford, CA, USA; h Division of Infectious Diseases and Geographic Medicine, Stanford University, Stanford, CA, USA; i Department of Environmental Science and Engineering, University of North Carolina, Gillings School of Global Public Health, Michael Hooker Research Center, Chapel Hill, NC, USA; j Department of Environmental and Occupational Health, Indiana University, Bloomington, IN, USA; k Department of Environmental Health, Rollins School of Public Health, Emory University, Atlanta, GA, USA; l Department of Environmental & Occupational Health Sciences, University of Washington, Seattle, WA, USA; m Department of Disease Control, London School of Tropical Medicine & Hygiene, London, UK; n Department of Microbiology and Environmental Toxicology, University of California, Santa Cruz, CA, USA; o Ministério da Saúde, Instituto Nacional de Saúde Maputo, Maputo, Mozambique; p Department of Civil and Environmental Engineering, College of Engineering, University of California, Berkeley, CA, USA; q Kenya Medical Research Institute, Nairobi, Kenya; r Mathematica, Princeton, NJ, USA; s Environmental Interventions Unit, Infectious Diseases Division, Dhaka, Bangladesh; t California Department of Toxic Substances Control, Sacramento, CA, USA; u Department of Forestry and Environmental Resources, North Carolina State University, Raleigh, NC, USA

## Abstract

**Background:**

Quantifying contributions of environmental faecal contamination to child diarrhoea and growth faltering can illuminate causal mechanisms behind modest health benefits in recent water, sanitation, and hygiene (WASH) trials. We aimed to assess associations between environmental detection of enteropathogens and human or animal microbial source tracking markers (MSTM) and subsequent child health outcomes.

**Methods:**

In this individual participant data meta-analysis we searched we searched PubMed, Embase, CAB Direct Global Health, Agricultural and Environmental Science Database, Web of Science, and Scopus for WASH intervention studies with a prospective design and concurrent control that measured enteropathogens or MSTM in environmental samples, or both, and subsequently measured enteric infections, diarrhoea, or height-for-age Z-scores (HAZ) in children younger than 5 years. We excluded studies that only measured faecal indicator bacteria. The initial search was done on Jan 19, 2021, and updated on March 22, 2023. One reviewer (AM) screened abstracts, and two independent reviewers (AM and RT) examined the full texts of short-listed articles. All included studies include at least one author that also contributed as an author to the present Article. Our primary outcomes were the 7-day prevalence of caregiver-reported diarrhoea and HAZ in children. For specific enteropathogens in the environment, primary outcomes also included subsequent child infection with the same pathogen ascertained by stool testing. We estimated associations using covariate-adjusted regressions and pooled estimates across studies.

**Findings:**

Data from nine published reports from five interventions studies, which included 8603 children (4302 girls and 4301 boys), were included in the meta-analysis. Environmental pathogen detection was associated with increased infection prevalence with the same pathogen and lower HAZ (ΔHAZ –0·09 [95% CI –0·17 to –0·01]) but not diarrhoea (prevalence ratio 1·22 [95% CI 0·95 to 1·58]), except during wet seasons. Detection of MSTM was not associated with diarrhoea (no pooled estimate) or HAZ (ΔHAZ –0·01 [–0·13 to 0·11] for human markers and ΔHAZ –0·02 [–0·24 to 0·21] for animal markers). Soil, children's hands, and stored drinking water were major transmission pathways.

**Interpretation:**

Our findings support a causal chain from pathogens in the environment to infection to growth faltering, indicating that the lack of WASH intervention effects on child growth might stem from insufficient reductions in environmental pathogen prevalence. Studies measuring enteropathogens in the environment should subsequently measure the same pathogens in stool to further examine theories of change between WASH, faecal contamination, and health. Given that environmental pathogen detection was predictive of infection, programmes targeting specific pathogens (eg, vaccinations and elimination efforts) can environmentally monitor the pathogens of interest for population-level surveillance instead of collecting individual biospecimens.

**Funding:**

The Bill & Melinda Gates Foundation and the UK Foreign and Commonwealth Development Office.

## Introduction

In settings with poor water, sanitation, and hygiene (WASH) conditions, children are exposed to enteric pathogens through environmentally mediated pathways. These exposures can lead to asymptomatic pathogen carriage, subclinical infection, or symptomatic diarrhoeal disease, and both subclinical changes to the gut and symptomatic diarrhoea can lead to nutrient loss and growth failure.^1^ Among children younger than 5 years, 62% of diarrhoea deaths and 16% of growth failure is attributed to poor WASH^2^ but several large trials of WASH interventions completed in the past decade found small or null effects on child diarrhoea and growth, despite high and sustained intervention uptake and robust study design, implementation, and outcome assessment.^3^ These results sparked debate about whether the interventions failed to reduce environmental faecal contamination, or whether environmental contamination from inadequate WASH in the home environment was not the primary cause of child diarrhoea or growth failure in those populations.

**Research in context**

**Evidence before this study**

Children living in poor drinking water, sanitation, and hygiene (WASH) conditions are exposed to enteric pathogens from faecal waste via environmentally mediated pathways such as drinking water, hands, food, soil, and flies. These exposures can result in gut colonisation with pathogens, which can lead to subclinical infections or diarrhoeal illness, which in turn can contribute to growth faltering. Recent large household-level and community-level WASH intervention studies that aimed to interrupt environmental pathogen transmission have had limited effects on children's health and on the detection of faecal indicator bacteria in the environment. These findings have generated substantial debate about whether basic WASH interventions do not sufficiently reduce environmental pathogen exposure to prevent disease in high-burden settings. In the past decade, applications of sensitive molecular methods in low-income countries allow simultaneous detection of multiple enteropathogens in environmental samples. Additionally, microbial source tracking (MST) methods can ideally help distinguish between human versus animal faeces, which pose different levels of health risk to the extent animals carry pathogens that can infect humans. Assessments using these methods can help illuminate the hypothesised causal chain between WASH improvements, environmental contamination, and child health. We reviewed the available evidence from studies published after Dec 31, 2020, and before the search date of Sept 16, 2023. We searched the PubMed and CABI Global Health databases using the search string “(diarrhea OR stunting OR wasting OR enteric infection) AND (microbial source tracking OR pathogen) AND (environmental measure OR fecal contamination)” to identify studies that detected enteropathogens or MST markers in the environment and investigated associations with child health outcomes. No language restrictions were applied to this search. The search returned 2741 publications, of which 13 were relevant to our research question. Of these, ten studies were from WASH intervention trials (and nine were included in our analysis), two studies investigated recreational water quality and swimmers’ diarrhoea in high-income settings, and one study examined pathogens in the environment and health in a low-income country (Tanzania).

**Added value of this study**

We used data from nine eligible publications reporting findings from five unique WASH intervention studies. Several pathogens in the environment were strongly associated with subsequent infection with the same pathogen in children. There was no overall association between pathogen detection in the environment and subsequent diarrhoea. Pooled across studies, pathogen detection in environmental samples was associated with slightly lower linear growth. Most human or animal MST markers were not associated with diarrhoea or child growth. Previous meta-analyses have linked faecal indicator bacteria in environmental samples to increased risk of diarrhoea and reduced linear growth in children. Data on health associations with enteropathogens and MST markers in the environment are scarce and mostly limited to high-income countries. This work is the first synthesis of evidence of the association between advanced environmental measurements and child health outcomes in low-income countries to examine causal pathways between WASH interventions and health.

**Implications of all the available evidence**

Enteropathogen detection in the environment was associated with increased risk of infection with the same pathogen and reduced child growth but not with caregiver-reported diarrhoea, supporting the causal chain leading from environmental pathogen exposure to infection to growth faltering. Our results also highlight the discordance between pathogen detection in the gut and symptomatic illness in settings where pathogen exposure is common, indicating that studies should augment self-reported diarrhoea outcomes with pathogen detection in stool. Our previous meta-analysis pooling data from the same WASH trials included in the current analysis found that the interventions had limited effects on environmental contamination with pathogens and MST markers. Together, these findings indicate that null effects on child growth in WASH trials completed in the past decade might stem from insufficient reductions in environmental exposure to pathogens. Our findings also indicate that environmental pathogen measurements are predictive of subsequent infection risk and can be used to characterise pathogen circulation to help target vaccines or other programmes and track progress for interventions on specific organisms. Environmental monitoring of specific pathogens as a population-level infection surveillance tool offers immense logistical advantages over collecting individual biospecimens from human populations, and our findings support recommendations to prioritise environmental sampling for disease surveillance in low-income countries. The reduction in height-for-age Z-scores associated with enteropathogen detection in the environment in our analysis was small and similar in magnitude to what has been reported for faecal indicator bacteria. These findings indicate that environmental faecal contamination measurements with current methods only partially explain growth faltering in children, regardless of choice of analytical target. The partial explanation could be because an environmental sample from a single location and time point does not adequately characterise environmental contamination or capture the frequency and duration of exposure, which determine the internal dose ultimately ingested by children. Future studies aiming to characterise faecal exposures and predict child growth outcomes should incorporate longitudinal and spatial environmental sampling with a combination of faecal indicator bacteria, enteropathogens, and sufficiently sensitive or specific MST markers. Faecal indicator bacteria might remain a useful tool as samples across time and space can be inexpensively analysed to capture variability.

Faecal contamination is usually measured using faecal indicator bacteria (FIB), which are associated with increased risk of diarrhoea and reduced growth in children.^4^ However, FIB are imperfect proxies of health risk as they can originate from non-faecal sources, and cannot confirm pathogen presence or differentiate between human and animal faeces, which carry different levels of health risk.^5^ Advances in multiplex nucleic acid-based methods allow simultaneous detection of a range of pathogens in environmental samples.^6^ Additionally, microbial source tracking methods aim to distinguish between human and animal faecal sources by targeting unique molecular characteristics of microorganisms strongly associated with the gastrointestinal tract of specific hosts.^7^ These methods are increasingly applied in studies in low-income countries to augment traditional FIB measurements and might better predict health risks; directly measuring enteropathogens in environmental matrices might more accurately represent exposure to disease-causing organisms, and detecting human-specific versus animal-specific microbial source tracking markers (MSTM) might indicate health risk of different magnitudes.^8^

We aimed to conducted an individual participant data (IPD) meta-analysis to assess associations between detection of enteropathogens and MSTM in different types of samples from the household environment and subsequently measured child health outcomes. Understanding to what extent these environmental measurements are associated with health outcomes can help illuminate the mechanisms behind the modest effects in recent WASH trials and guide the development of future interventions. If specific pathogens in specific environmental matrices are identified as dominant drivers of child diarrhoea and growth faltering, interventions can be tailored to interrupt these transmission mechanisms. If faecal contamination from specific animal species emerges as a risk factor, mitigation measures can target the management of these species.

## Methods

### Search strategy and selection criteria

In this individual participant data meta-analysis we searched PubMed, Embase, CAB Direct Global Health, Agricultural and Environmental Science Database, Web of Science, and Scopus to identify studies that (1) implemented a WASH intervention with a prospective design and concurrent control (ie, randomised controlled trial, matched cohort, and controlled before-and-after study), (2) measured pathogens or MSTM in environmental samples, or both, and (3) measured at least one of: pathogen-specific infections, diarrhoea, or child anthropometry. We limited the search to intervention studies to allow assessing intervention effects on environmental contamination as an additional objective. We were not aware of observational studies that report data on both environmental pathogens or MSTM and child health other than observational analyses nested within large trials, and attempting to identify and obtain individual-level data from a large and diffuse observational literature would have been prohibitive and unlikely to yield substantial additional data. We included studies published after Dec 31, 2000, to reflect recent advances in laboratory methods but we did not limit our search to any specific method (eg, molecular, culture-based, or microscopy). We excluded studies that only measured FIB. We limited our search to studies in English. The initial search was done on Jan 19, 2021, and updated on March 22, 2023. The search terms are listed in the appendix (pp 1, 23–26). Study authors were contacted but grey literature sources were not assessed. We only sought data from one unpublished study which was later published and included. One reviewer (AM) screened abstracts, and two independent reviewers (AM and RT) examined the full texts of short-listed articles, with differences resolved with a third reviewer (AE). We followed PRISMA guidelines (appendix pp 27–31). The protocol is available on Open Science Framework. All included studies include at least one author that also contributed as an author to the present Article.

### Data analysis

Our primary exposure variables were the prevalence of any enteropathogen and any MSTM in any type of environmental sample. We also tabulated exposure by sample type (eg, drinking water and hands). Secondary exposure variables included the prevalence of any viruses, any bacteria, any protozoa, or any helminths, prevalence of MSTM from humans versus specific animals, and prevalence and abundance of individual enteropathogens and MSTM. We excluded general (non-host-specific) MSTM. Our primary outcomes were the 7-day prevalence of caregiver-reported diarrhoea and height-for-age Z-scores (HAZ) in children. For specific enteropathogens in the environment, primary outcomes also included subsequent child infection with the same pathogen ascertained by stool testing. We note that pathogen detection in stool can indicate asymptomatic carriage, subclinical infection, or symptomatic infection; because we cannot discern between these possibilities, we use the term infection synonymously with pathogen detection in stool hereafter. Secondary outcomes included Z-scores for weight-for-age (WAZ), weight-for-height (WHZ), the prevalence of stunting (HAZ less than –2), underweight (WAZ less than –2), and wasting (WHZ less than –2).

We used a modified Newcastle-Ottawa scale, adapted for including randomised and quasi-randomised intervention studies, to assess bias in seven areas: selection bias, response bias, follow-up bias, misclassification bias, outcome assessment bias, ascertainment bias, and bias in the analysis.^9^

For diarrhoea and infection outcomes, we only used as exposures the environmental samples taken within 4 months of health outcome ascertainment; we selected this window empirically to maximise the number of available time-matched pairs of environmental and health measurements while maintaining exposure-disease time ordering. For HAZ outcomes, we used all environmental samples taken before health outcome ascertainment. If HAZ was measured multiple times, we used the measurement taken closest after environmental sampling.

We examined associations between environmental exposures and health outcomes within individual studies, using modified Poisson regression to estimate prevalence ratios for binary outcomes,^10^ and linear regression to estimate mean differences for continuous outcomes. We used the Huber Sandwich Estimator to calculate robust standard errors to account for repeated sampling or clustered designs.^11^ We adjusted for the following variables if measured within a given study: study group; child sex (as reported by caregiver); child age; maternal age; asset-based household wealth; household food security; number of people in household; age and education of primary caregiver; number of rooms in household; construction materials of the walls, floor, and roof; electricity access; land ownership; and agricultural occupation. We estimated associations for binary outcomes only if fewer than five cases of the outcome occurred in the rarest exposure stratum. For outcomes where data were available from four or more studies, we tested for heterogeneity using Cochran's Q-tests and *I*^2^ statistics and pooled study-specific effect estimates with random-effects models.

We conducted subgroup analyses by child age and sex, animal ownership, season, and study setting. For age, we used WHO motor milestones for immobile (age ≤254 days), crawling (age >254 days to 1 year), walking pre-school-age (age 1–5 years), and school-age (age >5 years).^12^ We defined animal ownership as the reported presence of any domestic animal in the compound (set of households that share a courtyard, water source, or latrine). We defined the wet season for each study as the 6 months of highest mean rainfall.^13^ Southeast Asia has a monsoon season (May to October), Kenya has two distinct rainy seasons (March to May and October to December), and Mozambique has a rainy season (November to April); our season definition aimed to capture these rainy periods. We assessed additive interactions by calculating prevalence differences with linear regression models that included interaction terms between the exposure and subgroup variables. Based on descriptions of study location, there was no variation in study setting (urban *vs* rural) within individual studies; we separately pooled estimates from urban versus rural studies and compared estimates with Wald tests.

As sensitivity analyses, we compared covariate-adjusted versus unadjusted estimates, and adjusted estimates from parametric regression models versus flexible machine learning-based targeted maximum likelihood estimation (TMLE).^14^ We re-estimated associations using environmental data collected within 1 month before diarrhoea measurements, and at any time with respect to diarrhoea measurements. We also estimated intervention effects on diarrhoea and HAZ within the subset of children with time-matched environmental samples. Analyses were conducted in R 4.0.4 and analysis scripts are publicly available.

All studies included in our review were approved by Institutional Review Boards (appendix p 34), and all participants of the original studies provided informed consent.

### Role of the funding source

The funders of the study had no role in study design, data collection, data analysis, data interpretation, or writing of the report.

## Results

**Figure 1:**
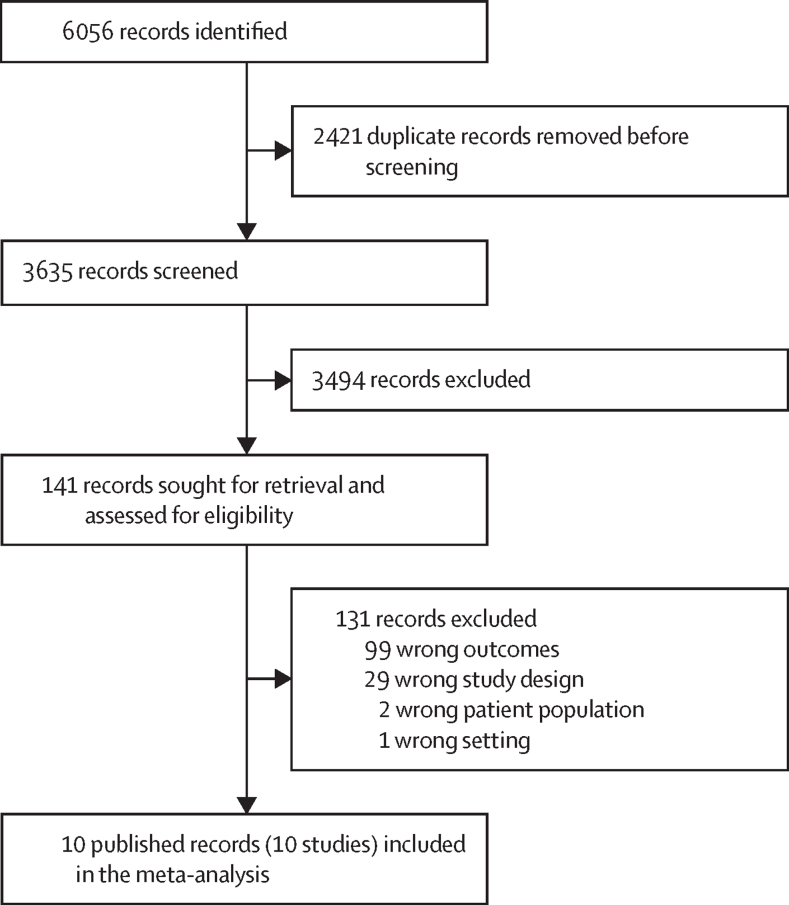
PRISMA flowchart

6056 studies were screened and after exclusions, nine published reports^15^, ^16^, ^17^, ^18^, ^19^, ^20^, ^21^, ^22^, ^23^ from five unique intervention studies were included in the meta-analysis (figure 1). The studies included the WASH Benefits Bangladesh^15^, ^16^, ^17^ and Kenya trials,^18^ the Maputo Sanitation (MapSan) trial in Mozambique,^19^, ^20^, ^21^ and the Gram Vikas^22^ and Total Sanitation Campaign^23^ studies in India (table). For the Total Sanitation Campaign, only shared village-level source water data were available. Individual publications nested within a trial sampled different subsets of participants at different times. Therefore, we report results by publication rather than by parent trial. Studies had moderate risk of bias due to the unblinded assessment of caregiver-reported diarrhoea. The Gram Vikas and MapSan studies had higher risk of bias due to higher loss to follow-up and no randomisation (appendix pp 32–33). The WASH interventions in the parent trials did not reduce child diarrhoea or growth faltering, except for the WASH Benefits Bangladesh trial in which children receiving sanitation, handwashing, and combined WASH interventions had lower diarrhoea prevalence, and in the Gram Vikas matched cohort study HAZ was higher in children receiving a combined piped water and sanitation intervention than in children receiving no intervention.^22^, ^24^ Among the subset of children with time-matched environmental data included in our IPD analysis, there was no intervention effects on child health in any study, except for WASH Benefits Kenya, where HAZ was lower in the combined WASH intervention arm (appendix p 2).^25^

**Table:** Descriptive statistics of child health outcomes by study

		**Reese (2017)**^22^	**Holcomb et al (2021)**^21^	**Capone et al (2021)**^19^	**Capone et al (2022)**^20^	**Odagiri et al (2016)**^23^	**Fuhrmeister et al (2020)**^16^	**Boehm et al (2016)**^17^	**Kwong et al (2021)**^15^	**Steinbaum et al (2019)**^18^
Parent trial or matched cohort		Gram Vikas	MapSan	MapSan	MapSan	Odisha Total Sanitation Campaign	WASH Benefits Bangladesh	WASH Benefits Bangladesh	WASH Benefits Bangladesh	WASH Benefits Kenya
Number of participants		505	183	198	223	1865	1119	497	2018	2279
Sex, n (%)										
	Girls	232 (45·9%)	88 (48·1%)	109 (55·1%)	103 (46·2%)	889 (47·7%)	562 (50·2%)	293 (59·0%)	1029 (51·0%)	1177 (51·6%)
	Boys	273 (54·1%)	95 (51·9%)	89 (44·9%)	120 (53·8%)	976 (52·3%)	557 (49·8%)	204 (41·0%)	989 (49·0%)	1102 (48·4%)
Number of distinct pathogens measured		..	..	15	10	..	1	..	2	2
Number of children with pathogens measured		..	..	96 (48·5%)	68 (30·5%)	..	261 (23·3%)	..	500 (24·8%)	1609 (70·6%)
Number pathogen infections detected		..	..	230	154	..	61	..	200	338
Pathogen prevalence		..	..	86·7%	83·9%	..	17·3%	..	23·4%	20·6%
Diarrhoea prevalence (n/N)		8·1% (17/210)	5·6% (4/72)	12·1% (12/99)	10·9% (10/92)	9·8% (163/1660)	12·1% (145/1202)	23·7% (97/409)	13·2% (140/1063)	25·9% (496/1912)
Mean HAZ (SD)		−1·78 (1·18)	−1·78 (1·49)	−1·55 (1·78)	−1·72 (1·65)	..	−1·8 (1·03)	−1·35 (1·07)	−1·58 (0·95)	−1·54 (1·09)
Stunting prevalence (n/N)		42·4 % (244/575)	48·5% (99/204)	43·2% (98/227)	42·2% (98/232)	..	40·6% (226/557)	26·3% (108/411)	30·1% (31/103)	31·6% (568/1800)
Mean WHZ (SD)		−0·87 (1·06)	0·35 (1·40)	0·09 (1·38)	0·11 (1·41)	..	−0·83 (0·94)	−0·74 (0·99)	−0·97 (0·84)	0·10 (0·95)
Wasting prevalence (n/N)		13·4% (77/574)	5·1% (10/197)	8·1% (18/221)	7·7% (17/220)	..	9·9% (55/558)	9·5% (39/412)	8·7% (9/103)	1·5% (27/1797)
Mean WAZ (SD)		..	−0·59 (1·08)	−0·68 (1·21)	−0·71 (1·15)	−1·36 (1·18)	−1·51 (1·04)	−1·35 (1·09)	−1·55 (0·92)	−0·73 (1·02)
Underweight prevalence (n/N)		..	8·0% (16/201)	11·4% (26/228)	11·6% (27/233)	29·2% (498/1703)	30·2% (171/567)	24·3% (100/412)	29·1% (30/103)	9·7% (180/1852)

Each column is for a specific study measuring different sets of environmental pathogens or microbial source tracking markers at different sampling times, with some nested within the same parent trial or matched cohort study. Pathogen-specific infection prevalence is the prevalence of at least one pathogen detected in child stools, and the number of pathogen infections is the total number of detected infections, whereby individual children can have infections from multiple pathogens. Health outcomes statistics only include health measurements occurring after environmental measures (and within 4 months for pathogen infections and diarrhoea). Distinct pathogens measured is the number of pathogens measured in both the environment and stool within a 4-month window. Stunting occurs when a child HAZ is less than −2, underweight is WAZ less than −2, and wasting is WHZ less than −2. HAZ=height-for-age Z-score. WAZ=weight-for-age Z-score. WHZ=weight-for-height Z-score.

The studies sampled source and stored drinking water, child and mother hand rinses, soil from the courtyard, household and latrine areas, food, and flies caught in the latrine and kitchen areas. Measured pathogens included bacteria (pathogenic *Escherichia coli, Vibrio cholerae, Shigella, Campylobacter jejuni* or *Campylobacter coli, Salmonella, Yersinia,* and *Clostridium difficile*), viruses (rotavirus, norovirus, sapovirus, adenovirus, astrovirus, and enterovirus), protozoa (*Cryptosporidium, Giardia,* and *Entamoeba histolytica*)*,* and helminths (*Ascaris lumbricoides* and *Trichuris trichiura*). Measured MSTM included markers for human (HumM2, HF183, BacHum, and *Methanobrevibacter smithii*), animal (BacCan and BacCow), ruminant (BacR), and avian (GFD) faecal hosts. Most studies used quantitative PCR. Additional details on study designs, sample collection, and laboratory methods of the individual studies are available elsewhere.^26^ The studies had health data from 8603 children (4302 girls and 4301 boys). The number of health observations with time-matched environmental samples ranged across the studies from 68 to 1609 for pathogen-specific infections, 72 to 1912 for diarrhoea, and 103 to 1800 for HAZ (table). Pathogen prevalence in children's stool was 17·3% to 86·7%, and diarrhoea prevalence was 5·6% to 25·9% (table). Mean HAZ ranged from –1·80 to –1·35 (table).

**Figure 2:**
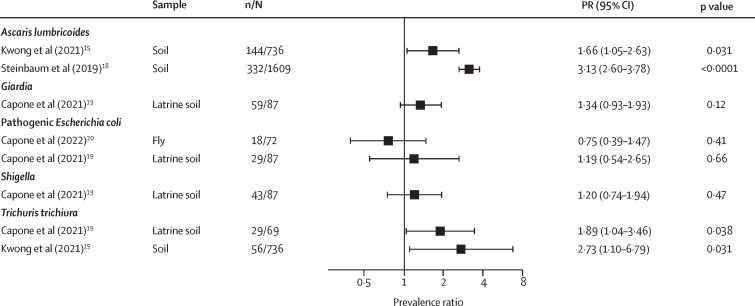
Forest plots of associations between specific enteropathogens in environmental samples and subsequent child infections with the same enteropathogens The presented prevalence ratios compare the detection prevalence of a pathogen in stool between children from compounds where the pathogen was detected versus not detected in environmental samples. Samples of the same type from different locations (eg, soil from courtyard *vs* latrine) are plotted separately and denoted by different colours. All estimates are adjusted for potential confounders.

Detection of a specific enteropathogen in the environment was consistently associated with higher prevalence of subsequent child infection with the same pathogen (figure 2). *Giardia*, *A lumbricoides*, and *T trichiura* detection in latrine and courtyard soil was associated with 1·3 times to 3·1 times higher prevalence of infection with these pathogens (figure 2).

**Figure 3:**
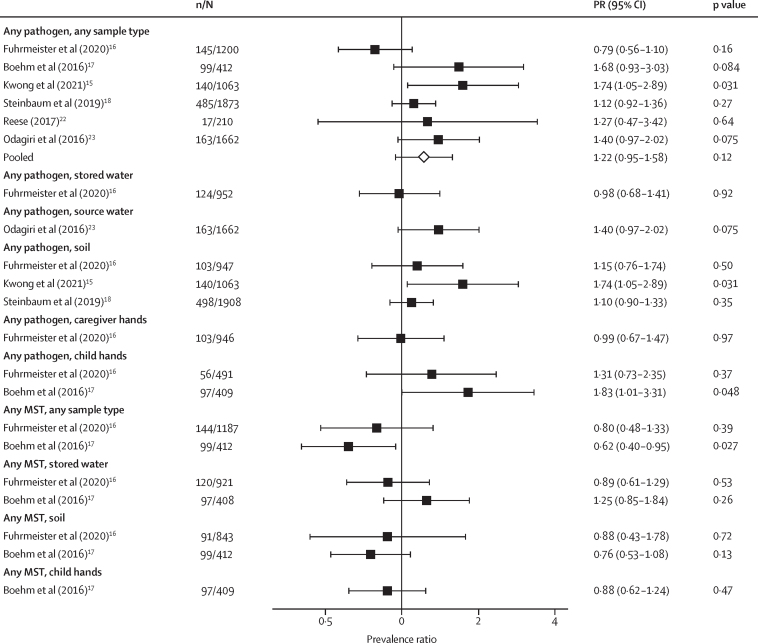
Forest plots of associations between the prevalence of any enteropathogen or any MSTM in different types of environmental samples and caregiver-reported child diarrhoeal disease The presented prevalence ratios compare diarrhoea prevalence between children from compounds where any pathogen or MSTM was detected versus not detected in environmental samples. Pooled estimates are presented when there are four or more study-specific estimates for a specific sample type and target combination and are denoted with diamond-shaped points. Grey crossed points denote that data were too sparse to estimate a prevalence ratio (ie, less than five cases in rarest exposure stratum). Samples of the same type from different locations (source *vs* stored water, flies in kitchen *vs* latrine, and soil from courtyard *vs* latrine) or different individuals (child's hands *vs* mother's hands) are plotted separately. All estimates are adjusted for potential confounders. MSTM=microbial source tracking marker.

Detection of any enteropathogen in any type of environmental sample was associated with higher diarrhoea prevalence in two studies,^15^, ^17^ but not when pooled across all studies (pooled prevalence ratio 1·22 [95% CI 0·95 to 1·58]; Q 10·2, *I*^2^ 57·5; figure 3). When analysed by pathogen group, detection of viruses on child hands^17^ and soil-transmitted helminths in soil was associated with higher diarrhoea prevalence in individual studies; other associations were non-significant (appendix pp 3–4). When analysed by individual pathogens, most associations with diarrhoea were non-significant, but detection of rotavirus on child hands,^17^ and *A lumbricoides* and *T trichiura* in household soil^15^, ^19^ was associated with 1·5 to 4·1 times higher diarrhoea prevalence in individual studies (appendix pp 5–6). Increasing abundance of *A lumbricoides* in household soil,^15^, ^17^ and rotavirus on child hands and in soil^17^ was also associated with higher diarrhoea prevalence (appendix p 7). The detection of any MSTM, or human-specific and animal-specific MSTM in any sample type was not associated with diarrhoea (no pooled estimates because there were less than four studies; figure 3; appendix p 4). Detection of the avian GFD marker on child hands was borderline associated with increased diarrhoea in one study;^17^ other individual markers were not associated with diarrhoea (appendix p 8).

**Figure 4:**
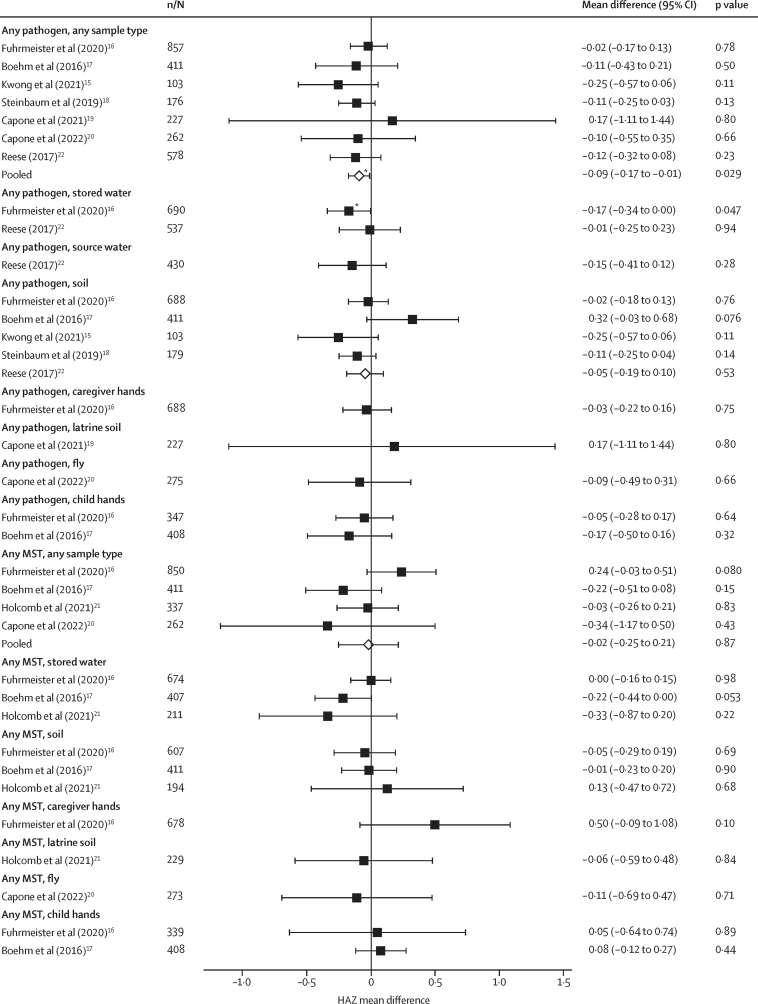
Forest plots of associations between the prevalence of any enteropathogen or any MSTM in different types of environmental samples and HAZ The presented differences compare HAZ between children from compounds where any pathogen or MSTM was detected versus not detected in environmental samples. Pooled estimates are presented when there are four or more study-specific estimates for a specific sample type and target combination and are denoted with diamond-shaped points. Grey crossed points denote that data were too sparse to estimate a mean difference. Samples of the same type from different locations (source *vs* stored water, flies in kitchen *vs* latrine, and soil from courtyard *vs* latrine) or different individuals (child's hands *vs* mother's hands) are plotted separately. All estimates are adjusted for potential confounders. HAZ=height-for-age Z-scores. MSTM=microbial source tracking marker.

Most studies showed slightly lower HAZ associated with enteropathogen detection in environmental samples but associations could not be distinguished from chance. Pooled across studies, detection of any enteropathogen in any sample type was significantly associated with lower HAZ (pooled ΔHAZ –0·09 [95% CI –0·17 to –0·01]; Q 2·3; *I*^2^; figure 4). When analysed by pathogen group, detection of bacteria in stored water and protozoa on child hands was associated with lower HAZ in one study (appendix p 3).^16^ When analysed by individual pathogens, detection of *A lumbricoides* in soil,^27^ pathogenic *E coli* in stored water,^16^ and *Giardia* on child hands^19^ was significantly associated with lower HAZ (ΔHAZ from –0·17 to –0·54; appendix pp 5–6). Many associations between individual pathogens and HAZ were non-significant, and several pathogens were associated with higher HAZ (appendix pp 5–6). Associations between the abundance of specific enteropathogens and HAZ, and between the presence or abundance of enteropathogens and WAZ, WHZ, stunting, and wasting were inconsistent (appendix p 5–7).

There was no association with HAZ for the detection of any MSTM (pooled ΔHAZ –0·02 [95% CI to –0·25 to 0·21]; Q 5·8; *I*^2^ 51·1; figure 4), any human-specific marker (pooled ΔHAZ –0·01 [–0·13 to 0·11]; Q 0·9; *I*^2^ 0; appendix p 4) or animal-specific marker (pooled ΔHAZ –0·02 [–0·24 to 0·21]; Q 5·0; *I*^2^ 41·0; appendix p 4) in any environmental sample. Associations between the presence or abundance of individual MSTM and growth measures were inconsistent and mostly non-significant (appendix pp 3–4, 8–9). When analysed by individual marker, detection of the BacCow animal marker in soil and the GFD avian and BacR ruminant markers in stored water was associated with reduced lateral and ponderal growth (Z from –0·28 to –0·76; appendix p 8). The abundance of MSTM had similar associations with health outcomes, though the abundance but not presence of BacCow in household soil and stored water was associated with lower HAZ and higher stunting prevalence, and the abundance but not presence of HumM2 in household soil was associated with higher stunting and wasting prevalence (appendix pp 8–9).

Associations did not vary consistently with child age (appendix pp 10–11); although, most studies did not have children measured in all age categories. Environmental pathogen detection was associated with twice the growth deficit in boys (ΔHAZ –0·12 [95% CI –0·24 to 0·01]) than in girls (ΔHAZ –0·06 [–0·18 to 0·07]); this pattern was supported in individual studies (appendix p 12). Child sex did not modify associations with diarrhoea (appendix p 13). Animal ownership did not modify any associations (appendix p 14). Environmental pathogen detection was associated with higher diarrhoea prevalence in the wet season (prevalence difference 0·05 [95% CI 0·003 to 0·09]) but not the dry season (appendix p 15); season did not modify other associations. Estimates did not differ between urban and rural studies.

Most covariates were not strongly associated with enteropathogen or MSTM presence in the environment, suggesting they are not strong confounders of the relationship between these exposures and child health (appendix pp 16–17). Adjusted estimates were slightly larger in magnitude than unadjusted estimates (appendix pp 18–19). Estimates were similar using parametric versus. TMLE models (appendix pp 20–22) and using environmental data from up to 4 months before, 1 month before, or any time with respect to diarrhoea measurements (appendix p 22).

Based on the Newcastle-Ottawa scale, intervention studies ranged from three to six out of a possible nine stars for study quality (appendix pp 32–33). The primary reasons for losing stars were the quasi-randomised nature of two intervention studies, the caregiver recall of child diarrhoeal disease instead of laboratory validation, and the absence of masking of participants and assessors to the intervention status.

## Discussion

Enteropathogen detection in the domestic environment was associated with higher prevalence of subsequent infection with the same pathogen and lower HAZ (pooled ΔHAZ –0·09 [95% CI –0·17 to –0·01]) but not diarrhoea (pooled prevalence ratio 1·22 [95% CI 0·95 to 1·58]) among children. Detection of any human or animal MSTM was not associated with diarrhoea (not enough studies for pooled estimate) or child growth (pooled ΔHAZ –0·01 [–0·13 to 0·11] for human markers, –0·02 [–0·24 to 0·21] for animal markers). Soil, child hands, and stored drinking water emerged as important transmission pathways. Our findings support a causal chain between environmental contamination with pathogens, enteric infection, and growth faltering. In a previous analysis, we found that the WASH interventions in our review had minimal effects on enteropathogen and MSTM detection in the domestic environment.^26^ Taken together, this set of findings indicates that small or non-significant child health effects in recent WASH trials stem from insufficient reductions in environmental pathogen exposure.

Notably, there was no overall association between pathogens in the environment and diarrhoea except during wet seasons. When children are frequently exposed to pathogens, asymptomatic colonisation and subclinical infections are common. A study of birth cohorts from sub-Saharan Africa, Asia, and South America detected one or more pathogen in 65% of non-diarrhoeal stools.^28^ In our analysis, diarrhoea prevalence was 6–26% and pathogen prevalence in stool was 17–87%, indicating common asymptomatic colonisation. Acquired pathogen-specific immunity and vaccines can affect the manifestation of diarrhoea following pathogen exposure,^29^ and non-pathogenic causes can cause diarrhoea symptoms. Caregiver-reported diarrhoea is also subject to poor recall and potential misclassification.^30^ In a study in Bangladesh, survey questions on diarrhoea symptoms, pictorial surveys, and visual assessment of stool had poor agreement with each other and low sensitivity and specificity against pathogen detection in stool.^31^ Our findings support recommendations to augment self-reported diarrhoea measurements with stool testing for enteric pathogens in future studies.^32^

Pathogens in the environment associated with increased diarrhoea in our analysis included *A lumbricoides*, *T trichiura,* and rotavirus. This finding could partly be because soil-transmitted helminths eggs and rotavirus are resilient and have prolonged survival in the environment.^33^, ^34^ Rotavirus has consistently been identified among the pathogens with the highest attributable burden of child diarrhoea in low-income countries.^28^, ^35^ Other dominant diarrhoeagenic pathogens in prior studies included *Cryptosporidium*, *Shigella*, *Campylobacter*, and norovirus. Although multiple studies in our review detected these pathogen targets, they did not subsequently measure diarrhoea within our 4-month window to allow estimating associations. Excluding these major drivers of paediatric diarrhoea from our analysis could contribute to the overall lack of associations between environmental pathogens and diarrhoea. We note that *A lumbricoides* and *T trichiura* infections typically do not lead to diarrhoeal symptoms;^36^ the associations we observed could reflect a chance finding, co-occurrence of other pathogens in soil, or residual confounding.

Detection of human MSTM was not associated with diarrhoea or growth. The GFD avian marker was marginally associated with diarrhoea and the GFD avian, BacR ruminant and BacCow animal markers were associated with reduced growth, but associations were inconsistent across different markers and health endpoints. The sensitivity or specificity of MSTM in identifying host faeces is imperfect and regionally variable.^37^ Studies in our review conducted local validation to select well performing markers but observed cross-detection between hosts (appendix p 1). The sensitivity and specificity of human markers is low in low-income country settings where faecal contamination is widespread and humans share microbiota with animals.^37^ Our findings support recommendations for developing MSTM that can better distinguish human and specific animal faecal sources in different settings.^38^ Notably, the GFD marker was the only MSTM associated with increased diarrhoea in our analysis, while multiple animal markers were associated with reduced linear and ponderal growth. These findings support evidence that exposure to animals, specifically poultry, contributes to enteric pathogen transmission and growth faltering.^39^ In validation studies in the parent trials, the GFD avian marker had greater than 80% sensitivity and specificity in Bangladesh^17^ and 78% sensitivity and 100% specificity in Mozambique.^21^ Our findings suggest that well performing MSTM can be a potentially useful tool for detecting zoonotic health risks.

Our analysis adds to a body of research on the relationship between faecal contamination and child health. In one study, in India (included in our analysis), detection of any pathogen in improved water sources and detection of human or animal markers in stored drinking water and on hands was associated with increased risk of diarrhoea.^23^ In Tanzania, a case-control study found that pathogenic *E coli* in stored water was associated with decreased child diarrhoea.^40^ Most other studies in low-income countries have characterised environmental faecal contamination using FIB, which have been associated with increased risk of diarrhoea.^41^ An IPD analysis found approximately 10% higher odds of diarrhoea for each log_10_ FIB increase in drinking water and on child hands.^4^ In the same analysis, HAZ decreased with each log_10_ FIB increase in drinking water (ΔHAZ=–0·04) and on fomites (ΔHAZ=–0·06),^4^ similar to the HAZ reduction associated with enteropathogen detection in the environment in our analysis (ΔHAZ=–0·09). Thus, advanced measures to characterise environmental contamination did not yield clearer insights over FIB with respect to predicting child diarrhoea or growth.

Notably, however, our findings of infection with a pathogen following its detection in the environment indicate that environmental monitoring of pathogens can be a useful population-level surveillance tool, akin to wastewater-based epidemiology, which has been successfully used to monitor SARS-CoV-2 in various settings, including low-income countries.^42^ A 2023 study in Kenya, Benin, and India found that detection of soil-transmitted helminths species in soil was strongly associated with infection with the same species and proposed soil-transmitted helminth monitoring in soil as a surveillance alternative to collecting stool samples from individuals.^43^ Environmental sampling for specific pathogens is logistically simpler, less intrusive, and less time-consuming than collecting human biospecimens, especially when infection prevalence is low (eg, pathogen close to elimination). For example, environmental surveillance for polioviruses is recommended for polio-endemic countries as part of polio eradication programmes.^44^, ^45^ Our findings provide scientific support for these recommendations and indicate that pathogen-specific environmental surveillance can be used to characterise pathogen circulation in a given area, detect hotspots to target for human or animal vaccination campaigns and other interventions, identify specific transmission pathways, and evaluate intervention effectiveness. Because screening for a comprehensive panel of pathogens remains cost-prohibitive and requires advanced facilities, efforts not targeting an individual pathogen can rely on FIB measurements and test for pathogens of interest in a subset for more nuanced information.

Regardless of the analytical target used, predicting health risks from environmental measurements has limitations. Although FIB are imperfect predictors of health risks,^5^ they can be measured inexpensively with minimal equipment. FIB also indicate viable organisms when (typically) enumerated by culture. Measuring pathogens and MSTM is more expensive and requires more extensive facilities. Therefore, the number of samples tested is often small and the prevalence and abundance of enteropathogens in the environment is low, limiting statistical precision. Also, although viability can be assessed by newer PCR methods, typically used molecular methods cannot determine viability. Additionally, the abundance of faecal organisms in the environment varies substantially temporally and spatially^46^, ^47^ so grab samples at one point in time and space are unlikely to adequately characterise contamination. In an analysis among beachgoers in the USA, averaging repeated *Enterococcus* measurements in recreational waters revealed associations with gastrointestinal illness among swimmers.^48^ Fine-grained longitudinal sampling of the domestic environment can better characterise faecal contamination in low-income countries; such sampling is more feasible using inexpensive and widely available FIB methods compared with pathogen-specific and MSTM methods analysed in this Article. Additionally, environmental measurements give little information about the dose ingested by children, which is determined by the duration and frequency of exposure in addition to the level of contamination.^49^ Children's contact patterns with environmental matrices vary with age and setting.^50^ Combining assessments of these patterns with environmental measurements might better predict health risks.

Our analysis had several limitations. Only a small number of studies were included, and few pathogens were measured in both environmental and stool samples. Due to the smaller sample sizes of environmental sampling, rare detection of many of the targets, and low diarrhoea prevalence in most studies, we could not estimate all exposure-outcome associations, and our estimates might have false negatives. The IPD approach allowed us to combine data across studies to increase statistical precision; IPD analyses with additional data from future studies might detect associations we missed. We did not correct for multiple comparisons; some of our observed associations might be false positives, especially when results were inconsistent across sample types and individual studies. Most covariates were weakly associated with the environmental measurements, and our estimates from unadjusted, parametrically adjusted, and TMLE models were similar. Therefore, we believe we adequately adjusted for measured confounding but residual confounding from unmeasured factors may remain. For the infection outcomes, although stool was sampled subsequent to environmental sampling, the observed associations could reflect reverse causation from chronic shedding by colonised children contaminating the environment. Differing periods between environmental and health measurements might also lead to inconsistent associations between studies. However, decreasing or increasing the window we allowed between environmental and diarrhoea measurements in our analyses did not change our findings.

In conclusion, enteropathogen detection in the environment was associated with increased risk of child enteric infections and slightly lower linear growth but not symptomatic diarrhoea in our analysis. Our findings support a causal chain between pathogen presence in the environment, child infection, and child growth, indicating that environmental monitoring of pathogens can be an effective tool for population-level infection surveillance. Our results also indicate a need for MSTM that can better differentiate faecal hosts in settings where humans and animals live in close proximity. To further assess links between environmental faecal exposure and child health, future research should collect longitudinal and spatial environmental samples, measure a combination of FIB and pathogens in the environment, and subsequently test for the same pathogens in stool.

## Data sharing

The de-identified, aggregated individual participant data, and data dictionary can be shared by request of investigators and after approval by the original trial data contributors. Data inquiries can be directed to the corresponding author. Statistical analysis code is available at https://github.com/amertens/wash-ipd.

## Declaration of interests

A portion of JK's salary is supported by an unrestricted donation to the London School of Hygiene & Tropical Medicine from Reckitt; this salary support is wholly unrelated to her role in the preparation of this manuscript. All other authors declare no competing interests.
