# Supplementary material for: Is detection of enteropathogens and human or animal faecal markers in the environment associated with subsequent child enteric infections and growth: an individual participant data meta-analysis
Source: Lancet Glob Health. 2024 Feb 14;12(3):e433–44. doi: 10.1016/S2214-109X(23)00563-6 (PMC10882208; doi:10.1016/S2214-109X(23)00563-6)
Supplement: Supplementary appendix [file mmc1.pdf]

THE LANCET
Global Health

Supplementary appendix

This appendix formed part of the original submission and has been peer reviewed. We post it as supplied by the authors.

Supplement to: Mertens A, Arnold BF, Benjamin-Chung J, et al. Is detection of enteropathogens and human or animal faecal markers in the environment associated with subsequent child enteric infections and growth: an individual participant data meta-analysis. *Lancet Glob Health* 2024; **12:** e433–44.

Supplementary Material

Is detection of enteropathogens and human or animal faecal markers in the environment associated with child enteric infections and growth: an individual participant data meta-analysis

SUPPLEMENTARY TEXT

Text S1. Literature review methods

We conducted a search of the PubMed, Embase, CAB Direct Global Health, Agricultural & Environmental Science Database, Web Of Science, and Scopus databases to identify studies that (1) implemented a WASH intervention with a prospective design and concurrent control (i.e., randomised controlled trial, matched cohort, controlled before-and-after study), (2) measured pathogens and/or microbial source tracking (MST) markers in environmental samples, and (3) measured at least one of: pathogen-specific infections, diarrhoea or child anthropometry. We limited the search to intervention studies to allow assessing intervention effects on environmental contamination as an additional objective.[1] We were not aware of observational studies that report data on both environmental pathogens/MST markers and child health other than observational analyses nested within large trials, and attempting to identify and obtain individual-level data from a large and diffuse observational literature would have been prohibitive while unlikely to yield substantial additional data. We included studies published after 2000 to reflect recent advances in laboratory methods but we did not limit our search to any specific method (e.g., molecular, culture-based, microscopy). We excluded studies that only measured fecal indicator bacteria. We limited our search to studies in English. One reviewer (Andrew Mertens) screened abstracts, and two independent reviewers (Andrew Mertens, Ruwan Thilakaratne) examined the full texts of short-listed articles with differences resolved with a third reviewer (Ayse Ercumen). The initial review was conducted on 19 January 2021 and was updated on 22 March 2023. The search terms are listed in Table S1.

Text S2. Sensitivity and specificity of microbial source tracking markers used in included studies

The accuracy of MST markers in identifying host faeces is imperfect, and sensitivity/specificity values >80% are considered adequate.[2] A validation study from 16 countries on six continents found that that the sensitivity of BacHum, BacCow and BacR was 87-92%, while host-specificity was 69% for BacHum, 57% for BacCow and 84% for BacR.[3] The studies in our analysis performed setting-specific validation to select the markers with the best demonstrated local performance. For the Total Sanitation Campaign trial, the selected human marker (BacHum) had <50% sensitivity and 78-80% specificity, and it cross-reacted with chicken feces.[4] The selected animal marker (BacCow) had 95% sensitivity and 100% specificity.[4] For WASH Benefits Bangladesh, the selected HumM2, BacR and GFD markers had >80% sensitivity and specificity tested against local human, chicken/duck, cow and goat faeces.[5] For MapSan, the selected human markers (HF183, *M. smithii*) had 64-71% sensitivity and 67-71% specificity, and they cross-reacted with avian feces.[6] The avian GFD marker had 78% sensitivity and 100% specificity. [6]

Supplementary References

1 Mertens A, Arnold BF, Benjamin-Chung J, *et al.* Effects of water, sanitation, and hygiene interventions on detection of enteropathogens and host-specific faecal markers in the environment: a systematic review and individual participant data meta-analysis. *The Lancet Planetary Health* 2023; **7**: e197–208.

2 Ahmed W, Hughes B, Harwood VJ. Current Status of Marker Genes of Bacteroides and Related Taxa for Identifying Sewage Pollution in Environmental Waters. *Water* 2016; **8**: 231.

3 Reischer GH, Ebdon JE, Bauer JM, *et al.* Performance characteristics of qPCR assays targeting human- and ruminant-associated bacteroidetes for microbial source tracking across sixteen countries on six continents. *Environ Sci Technol* 2013; **47**: 8548–56.

4 Odagiri M, Schriewer A, Hanley K, *et al.* Validation of Bacteroidales quantitative PCR assays targeting human and animal fecal contamination in the public and domestic domains in India. *Sci Total Environ* 2015; **502**: 462–70.

5 Boehm AB, Wang D, Ercumen A, *et al.* Occurrence of Host-Associated Fecal Markers on Child Hands, Household Soil, and Drinking Water in Rural Bangladeshi Households. *Environ Sci Technol Lett* 2016; **3**: 393–8.
6 Holcomb DA, Knee J, Sumner T, *et al.* Human fecal contamination of water, soil, and surfaces in households sharing poor-quality sanitation facilities in Maputo, Mozambique. *Int J Hyg Environ Health* 2020; **226**: 113496.

Supplementary Figures

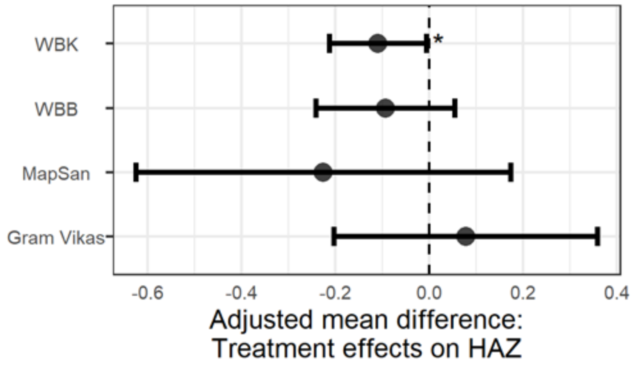

Figure S1. Water, sanitation and hygiene (WASH) intervention effects on child height-for-age Z-scores (HAZ) within the subset of children used in the primary analysis who had time-matched growth measurements and environmental samples. WBK: WASH Benefits Kenya; WBB: WASH Benefits Bangladesh, MapSan: Maputo Sanitation study, Gram Vikas: Gram Vikas matched cohort study.

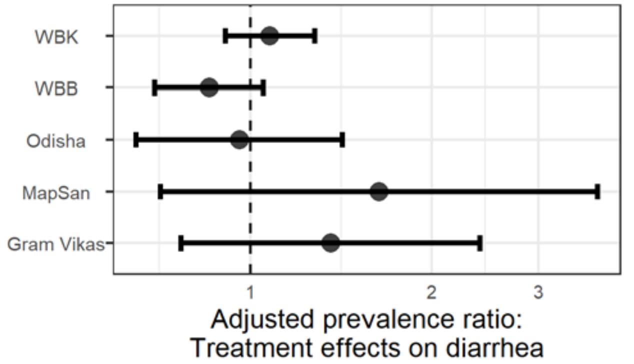

Figure S2. Water, sanitation and hygiene (WASH) intervention effects on child diarrhoeal disease within the subset of children used in the primary analysis who had time-matched diarrhoea observations and environmental samples. WBK: WASH Benefits Kenya; WBB: WASH Benefits Bangladesh, Odisha: Total Sanitation Campaign study; MapSan: Maputo Sanitation study, Gram Vikas: Gram Vikas matched cohort study.

Pathogen group	Sample	Diarrhoea	HAZ	WHZ	WAZ	Stunting	Wasting	Underweight
Any pathogen	**Any sample: POOLED**	1.22 (0.95, 1.58)	-0.09 (-0.17, -0.01)	0.00 (-0.12, 0.11)	-0.01 (-0.09, 0.06)	1.06 (0.96, 1.18)	1.05 (0.73, 1.52)	1.02 (0.89, 1.17)
	Any sample: Boehm 2016		-0.11 (-0.43, 0.21)	-0.16 (-0.40, 0.07)	-0.16 (-0.45, 0.13)			
	Any sample: Fuhrmeister 2020	0.79 (0.56, 1.10)	-0.02 (-0.17, 0.13)	-0.07 (-0.20, 0.05)	-0.01 (-0.16, 0.13)	1.19 (0.98, 1.44)	1.38 (0.80, 2.37)	1.05 (0.84, 1.33)
	Any sample: Reese 2017	1.27 (0.47, 3.42)	-0.12 (-0.32, 0.08)	0.12 (-0.10, 0.35)		0.98 (0.79, 1.21)	0.82 (0.45, 1.47)	
	Source water: Odagiri 2016	1.40 (0.97, 2.02)			0.00 (-0.16, 0.16)			0.97 (0.79, 1.19)
	Source water: Reese 2017		-0.15 (-0.41, 0.12)	0.26 (-0.09, 0.60)		1.21 (0.95, 1.54)	0.86 (0.41, 1.79)	
	Stored water: Fuhrmeister 2020	0.98 (0.68, 1.41)	-0.17 (-0.34, 0.00)	-0.03 (-0.17, 0.11)	-0.09 (-0.24, 0.07)	1.33 (1.11, 1.58)	1.28 (0.78, 2.09)	1.21 (0.96, 1.54)
	Stored water: Reese 2017		-0.01 (-0.25, 0.23)	0.10 (-0.12, 0.31)		0.79 (0.58, 1.07)	0.72 (0.36, 1.45)	
	Child hand rinse: Boehm 2016	1.83 (1.01, 3.31)	-0.17 (-0.50, 0.16)	-0.08 (-0.36, 0.20)	-0.14 (-0.48, 0.19)	1.04 (0.62, 1.76)		
	Child hand rinse: Fuhrmeister 2020	1.31 (0.73, 2.35)	-0.05 (-0.28, 0.17)	-0.04 (-0.21, 0.14)	0.00 (-0.21, 0.21)	1.29 (0.99, 1.66)	1.57 (0.96, 2.59)	1.20 (0.88, 1.64)
	Mother hand rinse: Fuhrmeister 2020	0.99 (0.67, 1.47)	-0.03 (-0.22, 0.16)	-0.01 (-0.17, 0.15)	-0.03 (-0.20, 0.15)	1.09 (0.90, 1.32)	0.95 (0.57, 1.59)	1.02 (0.81, 1.29)
	Latrine soil: Capone 2021		0.17 (-1.11, 1.44)	-0.17 (-0.66, 0.32)	-0.04 (-0.51, 0.43)	0.99 (0.53, 1.86)		
	House soil: POOLED		-0.05 (-0.19, 0.10)	0.02 (-0.11, 0.16)	0.00 (-0.09, 0.09)			
	House soil: Boehm 2016		0.32 (-0.03, 0.68)	0.07 (-0.47, 0.60)	0.29 (-0.14, 0.71)			
	House soil: Fuhrmeister 2020	1.15 (0.76, 1.74)	-0.02 (-0.18, 0.13)	-0.04 (-0.18, 0.10)	-0.02 (-0.16, 0.13)	1.10 (0.91, 1.32)	0.89 (0.56, 1.41)	1.10 (0.85, 1.42)
	House soil: Kwong 2021	1.74 (1.05, 2.89)	-0.25 (-0.57, 0.06)	-0.21 (-0.63, 0.21)	-0.30 (-0.69, 0.08)			1.85 (0.74, 4.61)
	House soil: Steinbaum 2019	1.10 (0.90, 1.33)	-0.11 (-0.25, 0.04)	0.12 (0.01, 0.23)	0.02 (-0.11, 0.15)	1.09 (0.91, 1.30)	1.41 (0.59, 3.40)	1.11 (0.79, 1.56)
	Flies: Capone 2022		-0.09 (-0.49, 0.31)	0.16 (-0.22, 0.54)	0.06 (-0.22, 0.34)	0.96 (0.71, 1.32)	0.61 (0.22, 1.65)	0.56 (0.28, 1.12)
Any bacteria	**Any sample: POOLED**		-0.05 (-0.16, 0.06)	-0.03 (-0.15, 0.10)	-0.01 (-0.10, 0.08)	0.98 (0.81, 1.19)		1.03 (0.90, 1.19)
	Any sample: Fuhrmeister 2020	0.81 (0.58, 1.13)	-0.04 (-0.19, 0.10)	-0.08 (-0.20, 0.05)	-0.03 (-0.17, 0.11)	1.21 (0.99, 1.46)	1.47 (0.85, 2.53)	1.09 (0.87, 1.37)
	Any sample: Reese 2017	1.27 (0.47, 3.42)	-0.12 (-0.32, 0.08)	0.12 (-0.10, 0.35)		0.98 (0.79, 1.21)	0.82 (0.45, 1.47)	
	Source water: Odagiri 2016	1.24 (0.84, 1.81)			-0.02 (-0.16, 0.13)			1.03 (0.85, 1.24)
	Source water: Reese 2017		-0.15 (-0.41, 0.12)	0.26 (-0.09, 0.60)		1.21 (0.95, 1.54)	0.86 (0.41, 1.79)	
	Stored water: Fuhrmeister 2020	0.98 (0.68, 1.41)	-0.17 (-0.34, 0.00)	-0.03 (-0.17, 0.11)	-0.09 (-0.24, 0.07)	1.33 (1.11, 1.58)	1.28 (0.78, 2.09)	1.21 (0.96, 1.54)
	Stored water: Reese 2017		-0.01 (-0.25, 0.23)	0.10 (-0.12, 0.31)		0.79 (0.58, 1.07)	0.72 (0.36, 1.45)	
	Child hand rinse: Fuhrmeister 2020	1.52 (0.88, 2.64)	-0.05 (-0.26, 0.17)	0.00 (-0.18, 0.19)	0.04 (-0.17, 0.24)	1.28 (1.01, 1.62)	1.44 (0.87, 2.41)	1.17 (0.86, 1.60)
	Mother hand rinse: Fuhrmeister 2020	1.11 (0.74, 1.66)	-0.07 (-0.27, 0.13)	0.01 (-0.16, 0.17)	-0.03 (-0.22, 0.15)	1.17 (0.97, 1.42)	0.85 (0.49, 1.48)	1.07 (0.84, 1.36)
	Latrine soil: Capone 2021		0.47 (-0.21, 1.15)	-0.20 (-0.71, 0.30)	0.28 (-0.10, 0.66)	0.73 (0.48, 1.09)		0.94 (0.31, 2.80)
	House soil: Fuhrmeister 2020	1.15 (0.76, 1.74)	-0.02 (-0.18, 0.13)	-0.04 (-0.18, 0.10)	-0.02 (-0.16, 0.13)	1.10 (0.91, 1.32)	0.89 (0.56, 1.41)	1.10 (0.85, 1.42)
	Flies: Capone 2022		0.02 (-0.37, 0.41)	0.00 (-0.35, 0.35)	-0.07 (-0.36, 0.23)	0.94 (0.70, 1.27)	0.67 (0.25, 1.85)	0.75 (0.38, 1.48)
Any virus	**Any sample: POOLED**		0.01 (-0.18, 0.19)	-0.12 (-0.32, 0.08)	-0.05 (-0.16, 0.05)	0.93 (0.76, 1.14)		1.03 (0.87, 1.22)
	Any sample: Boehm 2016		-0.11 (-0.43, 0.21)	-0.16 (-0.40, 0.07)	-0.16 (-0.45, 0.13)			
	Any sample: Fuhrmeister 2020		0.16 (-0.15, 0.47)	-0.33 (-0.65, -0.01)	-0.17 (-0.51, 0.18)	0.69 (0.38, 1.25)		1.18 (0.74, 1.88)
	Source water: Odagiri 2016	1.23 (0.77, 1.98)			-0.03 (-0.18, 0.11)			1.03 (0.84, 1.25)
	Child hand rinse: Boehm 2016	1.83 (1.01, 3.31)	-0.17 (-0.50, 0.16)	-0.08 (-0.36, 0.20)	-0.14 (-0.48, 0.19)	1.04 (0.62, 1.76)		
	Child hand rinse: Fuhrmeister 2020		0.11 (-0.44, 0.66)	-0.39 (-0.81, 0.03)	-0.23 (-0.71, 0.26)			1.40 (0.78, 2.50)
	Mother hand rinse: Fuhrmeister 2020		0.19 (-0.16, 0.53)	-0.25 (-0.63, 0.12)	-0.10 (-0.51, 0.31)			1.01 (0.55, 1.87)
	Latrine soil: Capone 2021		-0.02 (-0.54, 0.51)	-0.01 (-0.44, 0.42)	-0.11 (-0.42, 0.21)	0.83 (0.59, 1.19)	0.53 (0.19, 1.44)	0.91 (0.43, 1.94)
	House soil: Boehm 2016		0.32 (-0.03, 0.68)	0.07 (-0.47, 0.60)	0.29 (-0.14, 0.71)			
	Flies: Capone 2022		-0.06 (-0.48, 0.35)	0.27 (-0.17, 0.71)	0.17 (-0.15, 0.50)	1.11 (0.80, 1.53)		0.83 (0.38, 1.85)
Any protozoa	Any sample: Fuhrmeister 2020	1.54 (0.88, 2.70)	-0.36 (-0.71, 0.00)	0.00 (-0.41, 0.40)	-0.17 (-0.54, 0.21)			
	Child hand rinse: Fuhrmeister 2020		-0.54 (-0.96, -0.12)	-0.28 (-0.70, 0.15)	-0.45 (-0.89, -0.02)			1.53 (0.74, 3.17)
	Mother hand rinse: Fuhrmeister 2020		-0.21 (-0.73, 0.32)	0.27 (-0.36, 0.90)	0.10 (-0.40, 0.60)	1.14 (0.60, 2.16)		
	Latrine soil: Capone 2021		0.22 (-0.26, 0.70)	-0.05 (-0.40, 0.30)	0.07 (-0.26, 0.40)	0.81 (0.60, 1.11)	1.19 (0.42, 3.36)	1.24 (0.55, 2.80)
	Flies: Capone 2022		0.17 (-0.50, 0.85)	0.11 (-0.33, 0.55)	0.04 (-0.38, 0.45)	0.87 (0.51, 1.50)		
Any STH	**Any sample: POOLED**		-0.11 (-0.24, 0.02)	-0.03 (-0.23, 0.17)	-0.05 (-0.19, 0.09)			1.30 (0.98, 1.71)
	Latrine soil: Capone 2021		0.49 (-0.10, 1.07)	-0.20 (-0.55, 0.16)	-0.06 (-0.36, 0.25)	0.79 (0.55, 1.13)	0.69 (0.21, 2.30)	1.56 (0.65, 3.74)
	House soil: Kwong 2021	1.74 (1.05, 2.89)	-0.25 (-0.57, 0.06)	-0.21 (-0.63, 0.21)	-0.30 (-0.69, 0.08)			1.85 (0.74, 4.61)
	House soil: Steinbaum 2019	1.10 (0.90, 1.33)	-0.11 (-0.25, 0.04)	0.12 (0.01, 0.23)	0.02 (-0.11, 0.15)	1.09 (0.91, 1.30)	1.41 (0.59, 3.40)	1.11 (0.79, 1.56)
	Flies: Capone 2022		-0.23 (-1.00, 0.54)	-0.06 (-0.46, 0.33)	-0.15 (-0.58, 0.28)	1.04 (0.66, 1.65)		1.58 (0.83, 3.01)

P-value strength (estimate direction): **<0.01 (decreased risk)**; **<0.05 (decreased risk)**; **0.05-0.1 (decreased risk)**; **0.1-1**; **0.05-0.1 (increased risk)**; **<0.05 (increased risk)**; **<0.01 (increased risk)**; **Not estimated**

Any human or animal MST

	Diarrhoea	HAZ	WHZ	WAZ	Stunting	Wasting	Underweight
**Any sample: POOLED**		-0.02 (-0.25, 0.21)	-0.06 (-0.23, 0.12)	0.07 (-0.02, 0.16)	0.99 (0.74, 1.33)		
Any sample: Boehm 2016	0.62 (0.40, 0.95)	-0.22 (-0.51, 0.08)	-0.12 (-0.45, 0.20)	-0.22 (-0.56, 0.11)	1.85 (0.86, 3.97)		1.11 (0.55, 2.24)
Any sample: Fuhrmeister 2020	0.80 (0.48, 1.33)	0.24 (-0.03, 0.51)	-0.18 (-0.41, 0.05)	0.10 (-0.19, 0.38)	0.74 (0.57, 0.95)		0.72 (0.50, 1.03)
Any sample: Holcomb 2021		-0.03 (-0.26, 0.21)	0.10 (-0.16, 0.35)	0.07 (-0.08, 0.22)	1.05 (0.86, 1.27)	0.83 (0.35, 1.97)	1.00 (0.68, 1.46)
Source water: Odagiri 2016				0.13 (0.00, 0.26)			
Stored water: Boehm 2016	1.25 (0.85, 1.84)	-0.22 (-0.44, 0.00)	-0.18 (-0.40, 0.04)	-0.23 (-0.46, -0.01)	1.32 (0.96, 1.82)	1.07 (0.54, 2.09)	1.30 (0.90, 1.87)
Stored water: Fuhrmeister 2020	0.89 (0.61, 1.29)	0.00 (-0.16, 0.15)	0.00 (-0.14, 0.15)	0.01 (-0.14, 0.16)	1.04 (0.86, 1.26)	1.31 (0.76, 2.23)	0.97 (0.76, 1.24)
Stored water: Holcomb 2021		-0.33 (-0.87, 0.20)	0.16 (-0.30, 0.61)	0.03 (-0.37, 0.43)	1.28 (0.89, 1.84)		
Child hand rinse: Boehm 2016	0.88 (0.62, 1.24)	0.08 (-0.12, 0.27)	-0.11 (-0.31, 0.09)	-0.06 (-0.27, 0.15)	0.97 (0.69, 1.36)	1.01 (0.57, 1.79)	0.75 (0.53, 1.06)
Child hand rinse: Fuhrmeister 2020		0.05 (-0.64, 0.74)	0.10 (-0.51, 0.72)	-0.28 (-1.31, 0.75)			
Mother hand rinse: Fuhrmeister 2020		0.50 (-0.09, 1.08)	0.14 (-0.22, 0.51)	0.47 (-0.09, 1.04)	0.75 (0.47, 1.22)		0.54 (0.35, 0.82)
Latrine soil: Holcomb 2021		-0.06 (-0.59, 0.48)	0.22 (-0.30, 0.73)	0.16 (-0.09, 0.42)	0.98 (0.69, 1.40)		0.84 (0.41, 1.74)
House soil: Boehm 2016	0.76 (0.53, 1.08)	-0.01 (-0.23, 0.20)	-0.17 (-0.38, 0.05)	-0.13 (-0.36, 0.10)	1.14 (0.77, 1.69)	1.79 (0.86, 3.73)	0.96 (0.67, 1.36)
House soil: Fuhrmeister 2020	0.88 (0.43, 1.78)	-0.05 (-0.29, 0.19)	-0.30 (-0.54, -0.07)	-0.29 (-0.54, -0.03)	0.91 (0.67, 1.25)		1.25 (0.75, 2.08)
House soil: Holcomb 2021		0.13 (-0.47, 0.72)	-0.39 (-0.74, -0.04)	-0.38 (-0.73, -0.02)	1.14 (0.69, 1.90)		1.76 (1.00, 3.11)
Flies: Capone 2022		-0.11 (-0.69, 0.47)	0.08 (-0.40, 0.56)	0.00 (-0.38, 0.37)	1.05 (0.69, 1.61)		

Any human MST

	Diarrhoea	HAZ	WHZ	WAZ	Stunting	Wasting	Underweight
**Any sample: POOLED**		-0.01 (-0.13, 0.11)	0.04 (-0.13, 0.21)	0.05 (-0.06, 0.16)	1.04 (0.92, 1.18)		0.94 (0.82, 1.08)
Any sample: Boehm 2016		0.07 (-0.24, 0.38)	0.16 (-0.19, 0.52)	0.04 (-0.32, 0.40)			0.70 (0.36, 1.36)
Any sample: Fuhrmeister 2020	1.06 (0.78, 1.45)	0.00 (-0.17, 0.17)	-0.10 (-0.23, 0.04)	-0.09 (-0.24, 0.07)	1.01 (0.83, 1.22)	1.16 (0.73, 1.82)	1.01 (0.80, 1.28)
Any sample: Holcomb 2021		-0.03 (-0.25, 0.19)	0.13 (-0.12, 0.39)	0.10 (-0.06, 0.25)	1.06 (0.88, 1.27)	0.76 (0.38, 1.50)	0.96 (0.64, 1.43)
Source water: Odagiri 2016	1.00 (0.66, 1.52)			0.15 (0.01, 0.29)			0.92 (0.76, 1.11)
Stored water: Fuhrmeister 2020		0.00 (-0.56, 0.56)	0.08 (-0.40, 0.56)	-0.16 (-0.79, 0.47)	1.34 (0.84, 2.14)		
Stored water: Holcomb 2021		-0.32 (-0.89, 0.24)	0.31 (-0.18, 0.80)	0.17 (-0.27, 0.61)	1.28 (0.88, 1.86)		
Child hand rinse: Boehm 2016		-0.15 (-1.05, 0.74)	0.14 (-0.39, 0.67)	-0.47 (-1.38, 0.43)			
Child hand rinse: Fuhrmeister 2020	1.53 (0.82, 2.84)	-0.10 (-0.41, 0.20)	-0.17 (-0.43, 0.09)	-0.18 (-0.45, 0.09)	1.07 (0.78, 1.47)	1.68 (0.89, 3.18)	1.17 (0.77, 1.79)
Mother hand rinse: Fuhrmeister 2020	1.03 (0.63, 1.69)	-0.11 (-0.34, 0.12)	-0.03 (-0.23, 0.17)	-0.08 (-0.28, 0.13)	1.00 (0.76, 1.31)	1.13 (0.63, 2.02)	0.90 (0.64, 1.27)
Latrine soil: Holcomb 2021		-0.06 (-0.59, 0.48)	0.22 (-0.30, 0.73)	0.16 (-0.09, 0.42)	0.98 (0.69, 1.40)		0.84 (0.41, 1.74)
House soil: Boehm 2016	0.97 (0.50, 1.90)	0.19 (-0.12, 0.50)	0.18 (-0.18, 0.54)	0.26 (-0.10, 0.62)	0.69 (0.34, 1.43)		
House soil: Fuhrmeister 2020	1.02 (0.67, 1.55)	0.00 (-0.21, 0.22)	-0.01 (-0.17, 0.16)	0.00 (-0.19, 0.18)	0.91 (0.69, 1.21)	0.75 (0.41, 1.39)	0.90 (0.66, 1.24)
House soil: Holcomb 2021		0.23 (-0.36, 0.83)	-0.33 (-0.74, 0.09)	-0.27 (-0.68, 0.14)	1.11 (0.65, 1.89)		1.18 (0.49, 2.83)
Flies: Capone 2022		-0.02 (-0.48, 0.43)	0.07 (-0.32, 0.46)	0.00 (-0.31, 0.30)	1.05 (0.75, 1.47)		0.95 (0.50, 1.80)

Any animal MST

	Diarrhoea	HAZ	WHZ	WAZ	Stunting	Wasting	Underweight
**Any sample: POOLED**		-0.02 (-0.24, 0.21)	-0.09 (-0.25, 0.06)	-0.08 (-0.22, 0.06)	0.91 (0.72, 1.14)		1.29 (0.76, 2.18)
Any sample: Boehm 2016	0.63 (0.41, 0.97)	-0.11 (-0.42, 0.20)	-0.08 (-0.38, 0.23)	-0.12 (-0.45, 0.22)	1.49 (0.77, 2.87)		0.96 (0.53, 1.74)
Any sample: Fuhrmeister 2020	0.70 (0.44, 1.11)	0.21 (-0.05, 0.47)	-0.17 (-0.41, 0.07)	0.10 (-0.19, 0.38)	0.74 (0.58, 0.96)		0.74 (0.50, 1.08)
Any sample: Holcomb 2021		0.01 (-0.42, 0.44)	-0.04 (-0.42, 0.34)	-0.04 (-0.38, 0.30)	0.85 (0.60, 1.22)		
Source water: Odagiri 2016				-0.20 (-0.60, 0.20)			1.79 (1.32, 2.42)
Stored water: Boehm 2016	1.25 (0.85, 1.84)	-0.22 (-0.44, 0.00)	-0.18 (-0.40, 0.04)	-0.23 (-0.46, -0.01)	1.32 (0.96, 1.82)	1.07 (0.54, 2.09)	1.30 (0.90, 1.87)
Stored water: Fuhrmeister 2020	0.84 (0.58, 1.21)	0.00 (-0.15, 0.16)	-0.02 (-0.17, 0.12)	-0.01 (-0.16, 0.14)	1.04 (0.85, 1.28)	1.32 (0.76, 2.31)	1.01 (0.79, 1.30)
Child hand rinse: Boehm 2016	0.89 (0.63, 1.26)	0.12 (-0.09, 0.33)	-0.09 (-0.30, 0.11)	-0.01 (-0.24, 0.21)	0.92 (0.64, 1.31)	1.03 (0.58, 1.84)	0.71 (0.49, 1.02)
Child hand rinse: Fuhrmeister 2020		0.15 (-0.72, 1.02)	0.31 (-0.19, 0.82)	-0.19 (-1.46, 1.09)			
Mother hand rinse: Fuhrmeister 2020		0.37 (-0.26, 1.00)	0.11 (-0.29, 0.51)	0.39 (-0.21, 0.99)	0.82 (0.48, 1.40)		0.58 (0.36, 0.94)
Latrine soil: Holcomb 2021		-0.30 (-1.18, 0.58)	-0.20 (-0.88, 0.48)	-0.33 (-1.14, 0.49)			
House soil: Boehm 2016	0.74 (0.52, 1.06)	0.01 (-0.21, 0.22)	-0.15 (-0.36, 0.06)	-0.11 (-0.33, 0.11)	1.11 (0.76, 1.64)	1.55 (0.79, 3.03)	0.94 (0.67, 1.31)
House soil: Fuhrmeister 2020	0.70 (0.34, 1.42)	-0.15 (-0.41, 0.11)	-0.28 (-0.53, -0.02)	-0.33 (-0.61, -0.05)	1.06 (0.72, 1.58)		1.42 (0.79, 2.53)
House soil: Holcomb 2021		-0.29 (-0.80, 0.22)	-0.22 (-0.76, 0.33)	-0.25 (-0.75, 0.24)			
Flies: Capone 2022		-0.27 (-0.62, 0.07)	0.01 (-0.31, 0.32)	-0.16 (-0.39, 0.06)	1.07 (0.81, 1.39)	0.85 (0.33, 2.25)	1.98 (1.06, 3.72)

P-value strength (estimate direction): <0.01 (decreased risk); <0.05 (decreased risk); 0.05-0.1 (decreased risk); 0.1-1; 0.05-0.1 (increased risk); <0.05 (increased risk); <0.01 (increased risk); Not estimated

Figure S3. Heatmap of significance and direction of associations between aggregate measures of environmental contamination (rows) and child diarrhoea and growth outcomes (columns). Cells are colored by the strength of significance and direction of association, and the point estimate and confidence intervals are printed within cells, with relative risks for binary outcomes and mean differences for continuous outcomes. Each row is for a different sample type in a specific study or a pooled estimate across studies, and y-axis labels are colored by sample type, matching the primary figure legends. Estimates aggregated across any sample type are only plotted if there are multiple sample types for a study. Grey cells mark missing outcomes or exposure-outcome combinations too sparse to estimate. All estimates are adjusted for potential confounders.

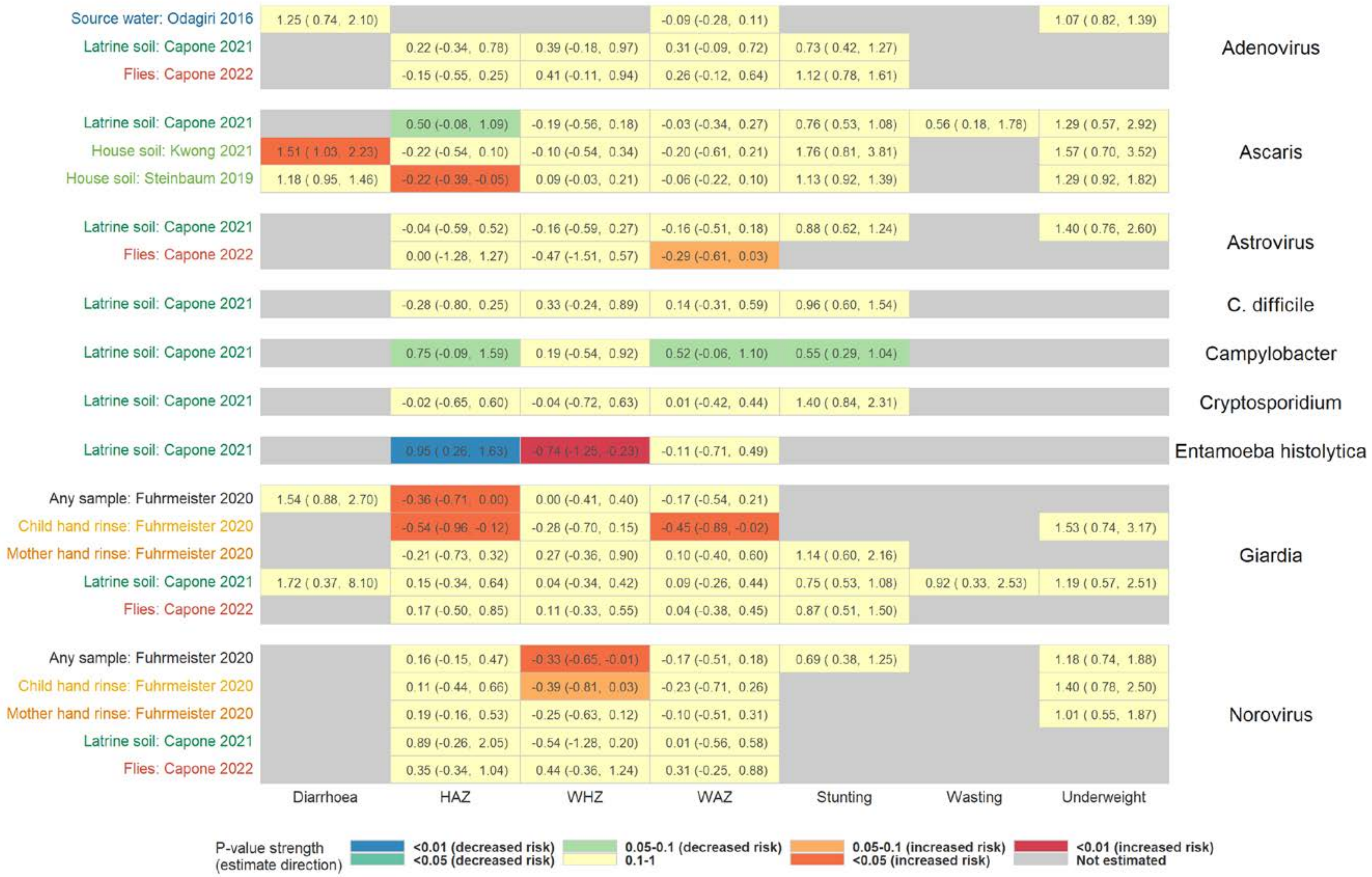
Source water: Odagiri 2016
1.25 (0.74, 2.10)
-0.09 (-0.28, 0.11)
1.07 (0.82, 1.39)
Latrine soil: Capone 2021
0.22 (-0.34, 0.78)
0.39 (-0.18, 0.97)
0.31 (-0.09, 0.72)
0.73 (0.42, 1.27)
Flies: Capone 2022
-0.15 (-0.55, 0.25)
0.41 (-0.11, 0.94)
0.26 (-0.12, 0.64)
1.12 (0.78, 1.61)
Adenovirus
Latrine soil: Capone 2021
0.50 (-0.08, 1.09)
-0.19 (-0.56, 0.18)
-0.03 (-0.34, 0.27)
0.76 (0.53, 1.08)
0.56 (0.18, 1.78)
1.29 (0.57, 2.92)
House soil: Kwong 2021
1.51 (1.03, 2.23)
-0.22 (-0.54, 0.10)
-0.10 (-0.54, 0.34)
-0.20 (-0.61, 0.21)
1.76 (0.81, 3.81)
1.57 (0.70, 3.52)
House soil: Steinbaum 2019
1.18 (0.95, 1.46)
-0.22 (-0.39, -0.05)
0.09 (-0.03, 0.21)
-0.06 (-0.22, 0.10)
1.13 (0.92, 1.39)
1.29 (0.92, 1.82)
Ascaris
Latrine soil: Capone 2021
-0.04 (-0.59, 0.52)
-0.16 (-0.59, 0.27)
-0.16 (-0.51, 0.18)
0.88 (0.62, 1.24)
1.40 (0.76, 2.60)
Flies: Capone 2022
0.00 (-1.28, 1.27)
-0.47 (-1.51, 0.57)
-0.29 (-0.61, 0.03)
Astrovirus
Latrine soil: Capone 2021
-0.28 (-0.80, 0.25)
0.33 (-0.24, 0.89)
0.14 (-0.31, 0.59)
0.96 (0.60, 1.54)
C. difficile
Latrine soil: Capone 2021
0.75 (-0.09, 1.59)
0.19 (-0.54, 0.92)
0.52 (-0.06, 1.10)
0.55 (0.29, 1.04)
Campylobacter
Latrine soil: Capone 2021
-0.02 (-0.65, 0.60)
-0.04 (-0.72, 0.63)
0.01 (-0.42, 0.44)
1.40 (0.84, 2.31)
Cryptosporidium
Latrine soil: Capone 2021
0.95 (0.26, 1.63)
-0.74 (-1.25, -0.23)
-0.11 (-0.71, 0.49)
Entamoeba histolytica
Any sample: Fuhrmeister 2020
1.54 (0.88, 2.70)
-0.36 (-0.71, 0.00)
0.00 (-0.41, 0.40)
-0.17 (-0.54, 0.21)
Child hand rinse: Fuhrmeister 2020
-0.54 (-0.96, -0.12)
-0.28 (-0.70, 0.15)
-0.45 (-0.89, -0.02)
1.53 (0.74, 3.17)
Mother hand rinse: Fuhrmeister 2020
-0.21 (-0.73, 0.32)
0.27 (-0.36, 0.90)
0.10 (-0.40, 0.60)
1.14 (0.60, 2.16)
Latrine soil: Capone 2021
1.72 (0.37, 8.10)
0.15 (-0.34, 0.64)
0.04 (-0.34, 0.42)
0.09 (-0.26, 0.44)
0.75 (0.53, 1.08)
0.92 (0.33, 2.53)
1.19 (0.57, 2.51)
Flies: Capone 2022
0.17 (-0.50, 0.85)
0.11 (-0.33, 0.55)
0.04 (-0.38, 0.45)
0.87 (0.51, 1.50)
Giardia
Any sample: Fuhrmeister 2020
0.16 (-0.15, 0.47)
-0.33 (-0.65, -0.01)
-0.17 (-0.51, 0.18)
0.69 (0.38, 1.25)
1.18 (0.74, 1.88)
Child hand rinse: Fuhrmeister 2020
0.11 (-0.44, 0.66)
-0.39 (-0.81, 0.03)
-0.23 (-0.71, 0.26)
1.40 (0.78, 2.50)
Mother hand rinse: Fuhrmeister 2020
0.19 (-0.16, 0.53)
-0.25 (-0.63, 0.12)
-0.10 (-0.51, 0.31)
1.01 (0.55, 1.87)
Latrine soil: Capone 2021
0.89 (-0.26, 2.05)
-0.54 (-1.28, 0.20)
0.01 (-0.56, 0.58)
Flies: Capone 2022
0.35 (-0.34, 1.04)
0.44 (-0.36, 1.24)
0.31 (-0.25, 0.88)
Norovirus
Diarrhoea
HAZ
WHZ
WAZ
Stunting
Wasting
Underweight
P-value strength
(estimate direction)
<0.01 (decreased risk)
<0.05 (decreased risk)
0.05-0.1 (decreased risk)
0.1-1
0.05-0.1 (increased risk)
<0.05 (increased risk)
<0.01 (increased risk)
Not estimated

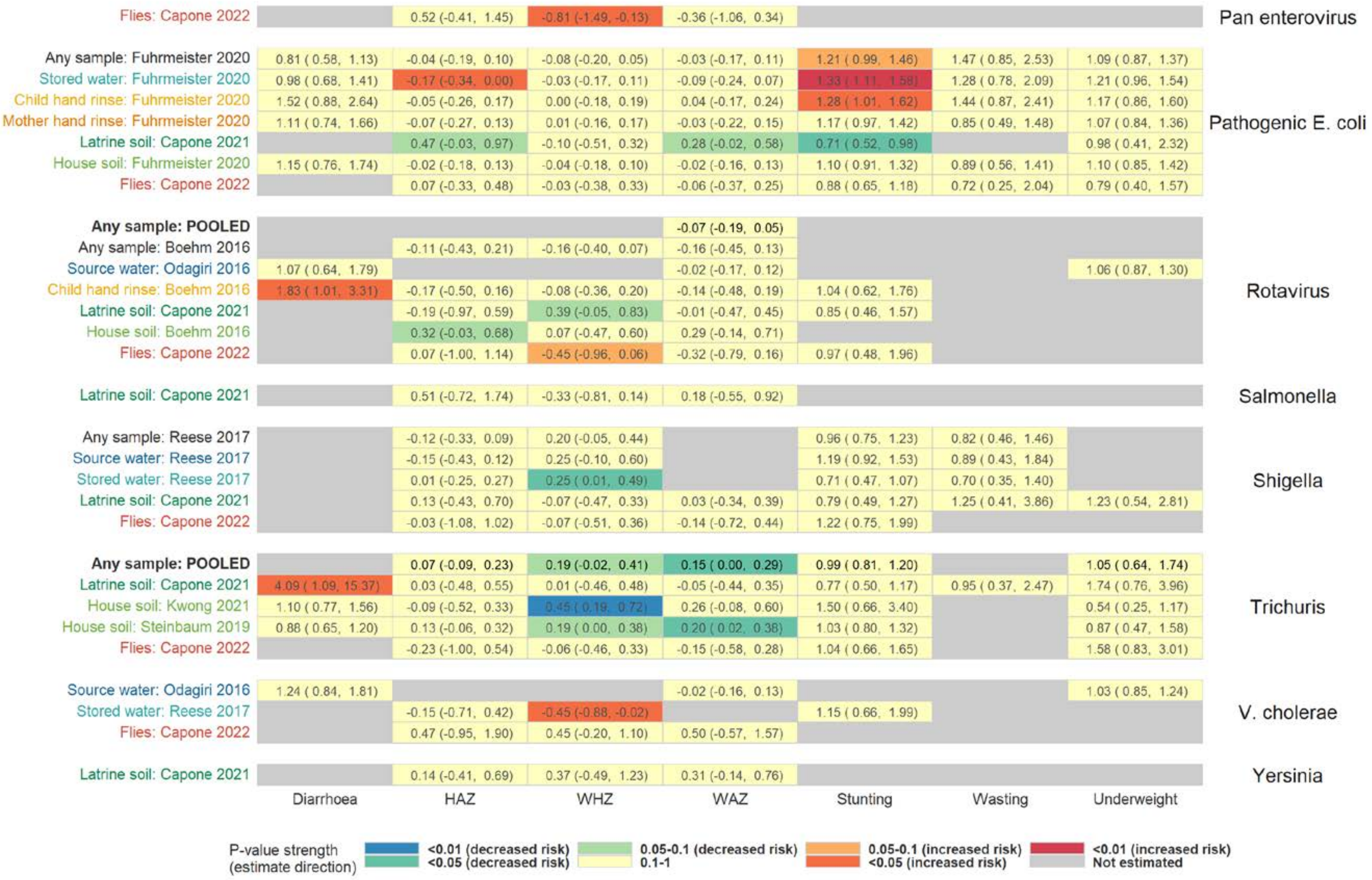

Figure S4. Heatmap of significance and direction of associations between the detection of specific pathogens in environmental samples (rows) and child diarrhoea and growth outcomes (columns). Cells are colored by the strength of significance and direction of association, and the point estimate and confidence intervals are printed within cells, with relative risks for binary outcomes and mean differences for continuous outcomes. Each row is for a different sample type in a specific study or a pooled estimate across studies, and y-axis labels are colored by sample type, matching the primary figure legends. Estimates aggregated across any sample type are only plotted if there are multiple sample types for a study. Grey cells mark missing outcomes or exposure-outcome combinations too sparse to estimate. All estimates are adjusted for potential confounders.

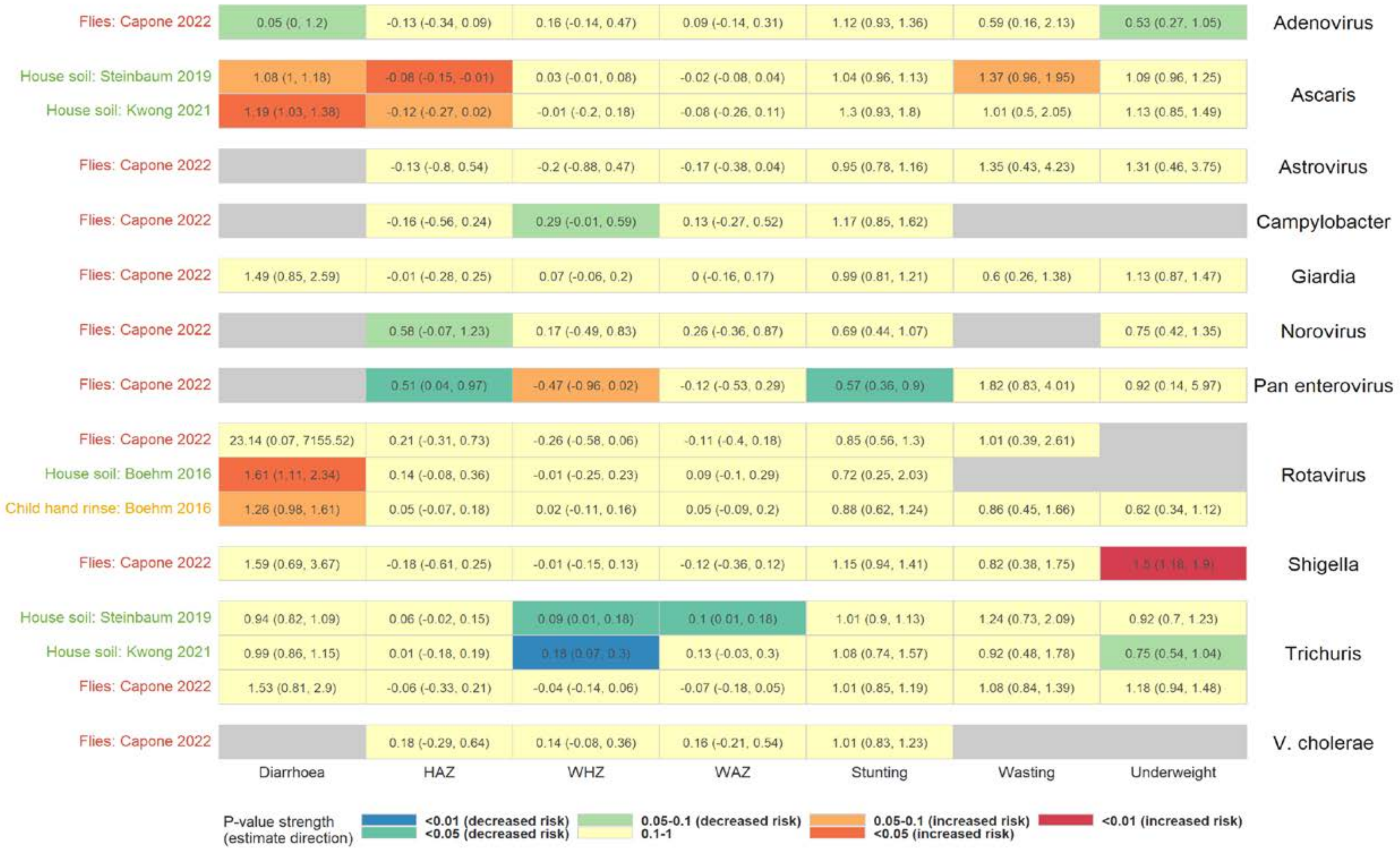

Figure S5. Heatmap of significance and direction of associations between the abundance of specific pathogens in environmental samples (rows) and child diarrhoea and growth outcomes (columns). Cells are colored by the strength of significance and direction of association, and the point estimate and confidence intervals are printed within cells, with relative risks for binary outcomes and mean differences for continuous outcomes. Each row is for a different sample type in a specific study or a pooled estimate across studies, and y-axis labels are colored by sample type, matching the primary figure legends. Estimates aggregated across any sample type are only plotted if there are multiple sample types for a study. Grey cells mark missing outcomes or exposure-outcome combinations too sparse to estimate. All estimates are adjusted for potential confounders.

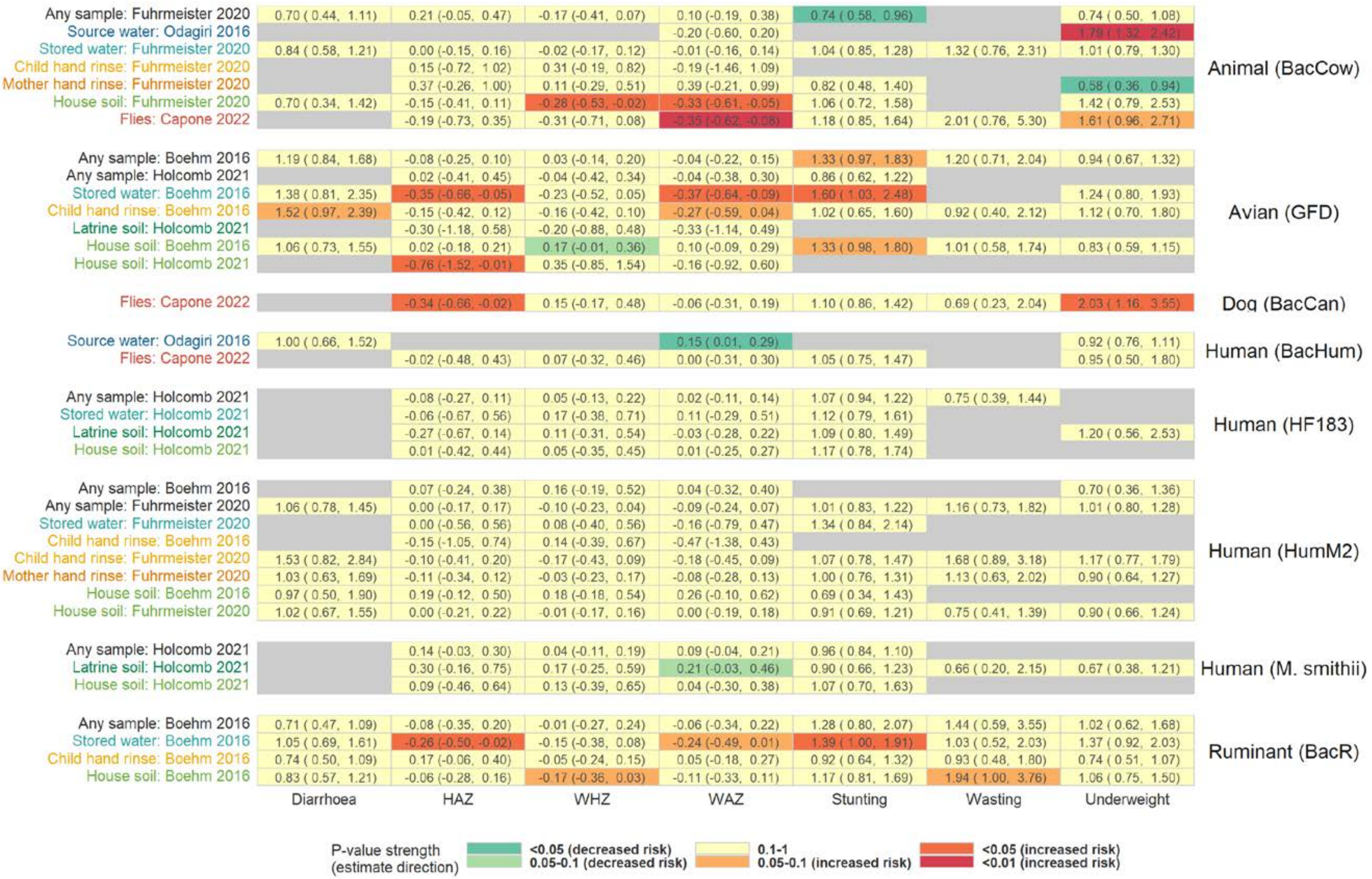

Marker	Sample: study	Diarrhoea	HAZ	WHZ	WAZ	Stunting	Wasting	Underweight
Animal (BacCow)	Any sample: Fuhrmeister 2020	0.70 (0.44, 1.11)	0.21 (-0.05, 0.47)	-0.17 (-0.41, 0.07)	0.10 (-0.19, 0.38)	0.74 (0.58, 0.96)		0.74 (0.50, 1.08)
	Source water: Odagiri 2016				-0.20 (-0.60, 0.20)			1.79 (1.32, 2.42)
	Stored water: Fuhrmeister 2020	0.84 (0.58, 1.21)	0.00 (-0.15, 0.16)	-0.02 (-0.17, 0.12)	-0.01 (-0.16, 0.14)	1.04 (0.85, 1.28)	1.32 (0.76, 2.31)	1.01 (0.79, 1.30)
	Child hand rinse: Fuhrmeister 2020		0.15 (-0.72, 1.02)	0.31 (-0.19, 0.82)	-0.19 (-1.46, 1.09)			
	Mother hand rinse: Fuhrmeister 2020		0.37 (-0.26, 1.00)	0.11 (-0.29, 0.51)	0.39 (-0.21, 0.99)	0.82 (0.48, 1.40)		0.58 (0.36, 0.94)
	House soil: Fuhrmeister 2020	0.70 (0.34, 1.42)	-0.15 (-0.41, 0.11)	-0.28 (-0.53, -0.02)	-0.33 (-0.61, -0.05)	1.06 (0.72, 1.58)		1.42 (0.79, 2.53)
	Flies: Capone 2022		-0.19 (-0.73, 0.35)	-0.31 (-0.71, 0.08)	-0.35 (-0.62, -0.08)	1.18 (0.85, 1.64)	2.01 (0.76, 5.30)	1.61 (0.96, 2.71)
Avian (GFD)	Any sample: Boehm 2016	1.19 (0.84, 1.68)	-0.08 (-0.25, 0.10)	0.03 (-0.14, 0.20)	-0.04 (-0.22, 0.15)	1.33 (0.97, 1.83)	1.20 (0.71, 2.04)	0.94 (0.67, 1.32)
	Any sample: Holcomb 2021		0.02 (-0.41, 0.45)	-0.04 (-0.42, 0.34)	-0.04 (-0.38, 0.30)	0.86 (0.62, 1.22)		
	Stored water: Boehm 2016	1.38 (0.81, 2.35)	-0.35 (-0.66, -0.05)	-0.23 (-0.52, 0.05)	-0.37 (-0.64, -0.09)	1.60 (1.03, 2.48)		1.24 (0.80, 1.93)
	Child hand rinse: Boehm 2016	1.52 (0.97, 2.39)	-0.15 (-0.42, 0.12)	-0.16 (-0.42, 0.10)	-0.27 (-0.59, 0.04)	1.02 (0.65, 1.60)	0.92 (0.40, 2.12)	1.12 (0.70, 1.80)
	Latrine soil: Holcomb 2021		-0.30 (-1.18, 0.58)	-0.20 (-0.88, 0.48)	-0.33 (-1.14, 0.49)			
	House soil: Boehm 2016	1.06 (0.73, 1.55)	0.02 (-0.18, 0.21)	0.17 (-0.01, 0.36)	0.10 (-0.09, 0.29)	1.33 (0.98, 1.80)	1.01 (0.58, 1.74)	0.83 (0.59, 1.15)
	House soil: Holcomb 2021		-0.76 (-1.52, -0.01)	0.35 (-0.85, 1.54)	-0.16 (-0.92, 0.60)			
Dog (BacCan)	Flies: Capone 2022		-0.34 (-0.66, -0.02)	0.15 (-0.17, 0.48)	-0.06 (-0.31, 0.19)	1.10 (0.86, 1.42)	0.69 (0.23, 2.04)	2.03 (1.16, 3.55)
Human (BacHum)	Source water: Odagiri 2016	1.00 (0.66, 1.52)			0.15 (0.01, 0.29)			0.92 (0.76, 1.11)
	Flies: Capone 2022		-0.02 (-0.48, 0.43)	0.07 (-0.32, 0.46)	0.00 (-0.31, 0.30)	1.05 (0.75, 1.47)		0.95 (0.50, 1.80)
Human (HF183)	Any sample: Holcomb 2021		-0.08 (-0.27, 0.11)	0.05 (-0.13, 0.22)	0.02 (-0.11, 0.14)	1.07 (0.94, 1.22)	0.75 (0.39, 1.44)	
	Stored water: Holcomb 2021		-0.06 (-0.67, 0.56)	0.17 (-0.38, 0.71)	0.11 (-0.29, 0.51)	1.12 (0.79, 1.61)		
	Latrine soil: Holcomb 2021		-0.27 (-0.67, 0.14)	0.11 (-0.31, 0.54)	-0.03 (-0.28, 0.22)	1.09 (0.80, 1.49)		1.20 (0.56, 2.53)
	House soil: Holcomb 2021		0.01 (-0.42, 0.44)	0.05 (-0.35, 0.45)	0.01 (-0.25, 0.27)	1.17 (0.78, 1.74)		
Human (HumM2)	Any sample: Boehm 2016		0.07 (-0.24, 0.38)	0.16 (-0.19, 0.52)	0.04 (-0.32, 0.40)			0.70 (0.36, 1.36)
	Any sample: Fuhrmeister 2020	1.06 (0.78, 1.45)	0.00 (-0.17, 0.17)	-0.10 (-0.23, 0.04)	-0.09 (-0.24, 0.07)	1.01 (0.83, 1.22)	1.16 (0.73, 1.82)	1.01 (0.80, 1.28)
	Stored water: Fuhrmeister 2020		0.00 (-0.56, 0.56)	0.08 (-0.40, 0.56)	-0.16 (-0.79, 0.47)	1.34 (0.84, 2.14)		
	Child hand rinse: Boehm 2016		-0.15 (-1.05, 0.74)	0.14 (-0.39, 0.67)	-0.47 (-1.38, 0.43)			
	Child hand rinse: Fuhrmeister 2020	1.53 (0.82, 2.84)	-0.10 (-0.41, 0.20)	-0.17 (-0.43, 0.09)	-0.18 (-0.45, 0.09)	1.07 (0.78, 1.47)	1.68 (0.89, 3.18)	1.17 (0.77, 1.79)
	Mother hand rinse: Fuhrmeister 2020	1.03 (0.63, 1.69)	-0.11 (-0.34, 0.12)	-0.03 (-0.23, 0.17)	-0.08 (-0.28, 0.13)	1.00 (0.76, 1.31)	1.13 (0.63, 2.02)	0.90 (0.64, 1.27)
	House soil: Boehm 2016	0.97 (0.50, 1.90)	0.19 (-0.12, 0.50)	0.18 (-0.18, 0.54)	0.26 (-0.10, 0.62)	0.69 (0.34, 1.43)		
	House soil: Fuhrmeister 2020	1.02 (0.67, 1.55)	0.00 (-0.21, 0.22)	-0.01 (-0.17, 0.16)	0.00 (-0.19, 0.18)	0.91 (0.69, 1.21)	0.75 (0.41, 1.39)	0.90 (0.66, 1.24)
Human (M. smithii)	Any sample: Holcomb 2021		0.14 (-0.03, 0.30)	0.04 (-0.11, 0.19)	0.09 (-0.04, 0.21)	0.96 (0.84, 1.10)		
	Latrine soil: Holcomb 2021		0.30 (-0.16, 0.75)	0.17 (-0.25, 0.59)	0.21 (-0.03, 0.46)	0.90 (0.66, 1.23)	0.66 (0.20, 2.15)	0.67 (0.38, 1.21)
	House soil: Holcomb 2021		0.09 (-0.46, 0.64)	0.13 (-0.39, 0.65)	0.04 (-0.30, 0.38)	1.07 (0.70, 1.63)		
Ruminant (BacR)	Any sample: Boehm 2016	0.71 (0.47, 1.09)	-0.08 (-0.35, 0.20)	-0.01 (-0.27, 0.24)	-0.06 (-0.34, 0.22)	1.28 (0.80, 2.07)	1.44 (0.59, 3.55)	1.02 (0.62, 1.68)
	Stored water: Boehm 2016	1.05 (0.69, 1.61)	-0.26 (-0.50, -0.02)	-0.15 (-0.38, 0.08)	-0.24 (-0.49, 0.01)	1.39 (1.00, 1.91)	1.03 (0.52, 2.03)	1.37 (0.92, 2.03)
	Child hand rinse: Boehm 2016	0.74 (0.50, 1.09)	0.17 (-0.06, 0.40)	-0.05 (-0.24, 0.15)	0.05 (-0.18, 0.27)	0.92 (0.64, 1.32)	0.93 (0.48, 1.80)	0.74 (0.51, 1.07)
	House soil: Boehm 2016	0.83 (0.57, 1.21)	-0.06 (-0.28, 0.16)	-0.17 (-0.36, 0.03)	-0.11 (-0.33, 0.11)	1.17 (0.81, 1.69)	1.94 (1.00, 3.76)	1.06 (0.75, 1.50)

Figure S6. Heatmap of significance and direction of associations between the detection of specific microbial source tracking markers in environmental samples (rows) and child diarrhoea and growth outcomes (columns). Cells are colored by the strength of significance and direction of association, and the point estimate and confidence intervals are printed within cells, with relative risks for binary outcomes and mean differences for continuous outcomes. Each row is for a different sample type in a specific study or a pooled estimate across studies, and y-axis labels are colored by sample type, matching the primary figure legends. Estimates aggregated across any sample type are only plotted if there are multiple sample types for a study. Grey cells mark missing outcomes or exposure-outcome combinations too sparse to estimate. All estimates are adjusted for potential confounders.

Sample	Diarrhoea	HAZ	WHZ	WAZ	Stunting	Wasting	Underweight	Marker
Flies: Capone 2022	0.41 (0.13, 1.28)	-0.05 (-0.22, 0.11)	-0.09 (-0.27, 0.08)	-0.09 (-0.21, 0.02)	1.03 (0.94, 1.12)	1.33 (0.9, 1.99)		Animal (BacCow)
Stored water: Fuhrmeister 2020	0.77 (0.56, 1.06)	-0.13 (-0.27, 0)	0 (-0.11, 0.1)	-0.09 (-0.21, 0.02)	1.16 (1.04, 1.29)	0.73 (0.44, 1.22)	1.1 (0.94, 1.29)	
House soil: Fuhrmeister 2020	0.96 (0.85, 1.07)	-0.07 (-0.12, -0.03)	-0.02 (-0.06, 0.02)	-0.06 (-0.1, -0.01)	1.11 (1.05, 1.17)	1.01 (0.88, 1.16)	1.07 (1, 1.16)	
Mother hand rinse: Fuhrmeister 2020	0.94 (0.81, 1.09)	0.01 (-0.05, 0.06)	-0.02 (-0.07, 0.03)	-0.01 (-0.07, 0.05)	1.02 (0.95, 1.09)	1 (0.85, 1.19)	0.99 (0.91, 1.08)	
Child hand rinse: Fuhrmeister 2020	1.04 (0.85, 1.26)	0.01 (-0.07, 0.1)	-0.03 (-0.1, 0.05)	-0.03 (-0.11, 0.06)	1 (0.91, 1.1)	1.32 (1.03, 1.71)	0.99 (0.87, 1.12)	
Stored water: Holcomb 2021		0.35 (0.03, 0.68)	-0.15 (-0.53, 0.22)	0.11 (-0.07, 0.29)	0.93 (0.73, 1.18)	1 (0.31, 3.24)	0.94 (0.56, 1.57)	Avian (GFD)
Source water: Holcomb 2021		0.27 (-0.11, 0.65)	-0.23 (-0.58, 0.13)	-0.08 (-0.32, 0.16)	0.95 (0.68, 1.32)	1.3 (0.7, 2.43)	1.46 (0.76, 2.81)	
House soil: Holcomb 2021		0.4 (-0.13, 0.92)	-0.13 (-0.68, 0.43)	-0.01 (-0.33, 0.31)	0.81 (0.54, 1.2)	1.51 (0.46, 4.98)	1.17 (0.56, 2.45)	
Latrine soil: Holcomb 2021		0.32 (-0.03, 0.66)	-0.23 (-0.55, 0.08)	-0.05 (-0.28, 0.17)	0.85 (0.64, 1.12)	1.37 (0.54, 3.44)	1.82 (1.12, 2.94)	
Stored water: Boehm 2016	1.73 (0.76, 3.94)	-0.62 (-1.11, -0.13)	-0.3 (-0.79, 0.19)	-0.59 (-1.07, -0.12)	2.13 (1.12, 4.06)		1.48 (0.76, 2.89)	
House soil: Boehm 2016	1.37 (0.81, 2.31)	0.04 (-0.22, 0.31)	0.1 (-0.16, 0.37)	0.04 (-0.2, 0.27)	1.28 (0.84, 1.96)	0.96 (0.43, 2.15)	0.78 (0.47, 1.29)	
Child hand rinse: Boehm 2016	1.66 (0.86, 3.19)	0.03 (-0.42, 0.48)	0.03 (-0.36, 0.43)	-0.11 (-0.61, 0.4)	0.86 (0.43, 1.73)	0.4 (0.08, 1.94)	0.91 (0.42, 1.99)	
Flies: Capone 2022	1.83 (0.97, 3.42)	-0.08 (-0.22, 0.06)	0.07 (-0.09, 0.23)	0.01 (-0.1, 0.12)	0.98 (0.89, 1.09)	0.83 (0.52, 1.32)	1.26 (1.03, 1.54)	Dog (BacCan)
Flies: Capone 2022	1.4 (0.93, 2.09)	0.03 (-0.13, 0.2)	0.03 (-0.07, 0.14)	0.07 (-0.03, 0.17)	0.99 (0.9, 1.1)	0.86 (0.64, 1.17)	0.88 (0.71, 1.09)	Human (BacHum)
Stored water: Holcomb 2021		-0.06 (-0.39, 0.26)	-0.02 (-0.28, 0.25)	0 (-0.21, 0.22)	1.2 (0.98, 1.48)	1.03 (0.5, 2.11)	0.9 (0.55, 1.47)	Human (HF183)
Source water: Holcomb 2021		-0.13 (-0.79, 0.53)	0.36 (-0.23, 0.95)	0.2 (-0.12, 0.52)	0.83 (0.54, 1.26)	0.62 (0.29, 1.31)	0.82 (0.34, 2)	
House soil: Holcomb 2021		-0.13 (-0.63, 0.37)	0.29 (-0.13, 0.71)	0.02 (-0.23, 0.27)	1.12 (0.77, 1.64)	0.2 (0.07, 0.54)	0.57 (0.19, 1.71)	
Latrine soil: Holcomb 2021		-0.2 (-0.52, 0.13)	0.11 (-0.18, 0.4)	-0.02 (-0.2, 0.16)	1.1 (0.86, 1.42)	0.88 (0.31, 2.5)	1.09 (0.63, 1.87)	
House soil: Boehm 2016	1.69 (0.6, 4.77)	-0.11 (-1.34, 1.13)	-0.37 (-0.93, 0.2)	-0.33 (-1.42, 0.76)	2.01 (1.08, 3.72)	2.31 (1.03, 5.17)	1.33 (0.59, 2.99)	Human (HumM2)
Stored water: Holcomb 2021		0.64 (-0.58, 1.85)	-0.31 (-1.68, 1.05)	0.21 (-0.64, 1.06)	1.6 (0.67, 3.8)	5.86 (0.19, 179.92)	2.57 (0.29, 23.21)	Human (M. smithii)
Source water: Holcomb 2021		0.92 (-0.32, 2.16)	-0.54 (-1.71, 0.62)	-0.08 (-0.78, 0.63)	0.66 (0.21, 2.07)	1.35 (0.2, 9.09)	2.21 (0.36, 13.42)	
House soil: Holcomb 2021		0.14 (-0.57, 0.84)	-0.03 (-0.61, 0.55)	-0.04 (-0.49, 0.42)	1.05 (0.61, 1.82)	0.45 (0.11, 1.84)	0.77 (0.18, 3.31)	
Latrine soil: Holcomb 2021		0.22 (-0.26, 0.69)	0.17 (-0.24, 0.59)	0.2 (-0.03, 0.43)	1.09 (0.82, 1.44)	0.76 (0.29, 1.99)	0.46 (0.24, 0.89)	
Stored water: Boehm 2016	0.85 (0.53, 1.36)	-0.15 (-0.35, 0.06)	-0.11 (-0.31, 0.1)	-0.16 (-0.38, 0.05)	1.06 (0.72, 1.57)	0.79 (0.38, 1.68)	1.13 (0.74, 1.73)	Ruminant (BacR)
House soil: Boehm 2016	1.08 (0.8, 1.45)	0.01 (-0.18, 0.19)	-0.02 (-0.2, 0.16)	0 (-0.19, 0.19)	1.13 (0.87, 1.48)	1.19 (0.73, 1.94)	0.93 (0.64, 1.35)	
Child hand rinse: Boehm 2016	0.62 (0.38, 1.01)	0.21 (-0.02, 0.44)	0.01 (-0.17, 0.2)	0.09 (-0.12, 0.3)	0.76 (0.5, 1.13)	0.71 (0.37, 1.35)	0.59 (0.38, 0.92)	

P-value strength (estimate direction): <0.01 (decreased risk); <0.05 (decreased risk); 0.05-0.1 (decreased risk); 0.1-1; 0.05-0.1 (increased risk); <0.05 (increased risk); <0.01 (increased risk)

Figure S7. Heatmap of significance and direction of associations between the abundance of specific microbial source tracking markers in environmental samples (rows) and child diarrhoea and growth outcomes (columns). Cells are colored by the strength of significance and direction of association, and the point estimate and confidence intervals are printed within cells, with relative risks for binary outcomes and mean differences for continuous outcomes. Each row is for a different sample type in a specific study or a pooled estimate across studies, and y-axis labels are colored by sample type, matching the primary figure legends. Estimates aggregated across any sample type are only plotted if there are multiple sample types for a study. Grey cells mark missing outcomes or exposure-outcome combinations too sparse to estimate. All estimates are adjusted for potential confounders.

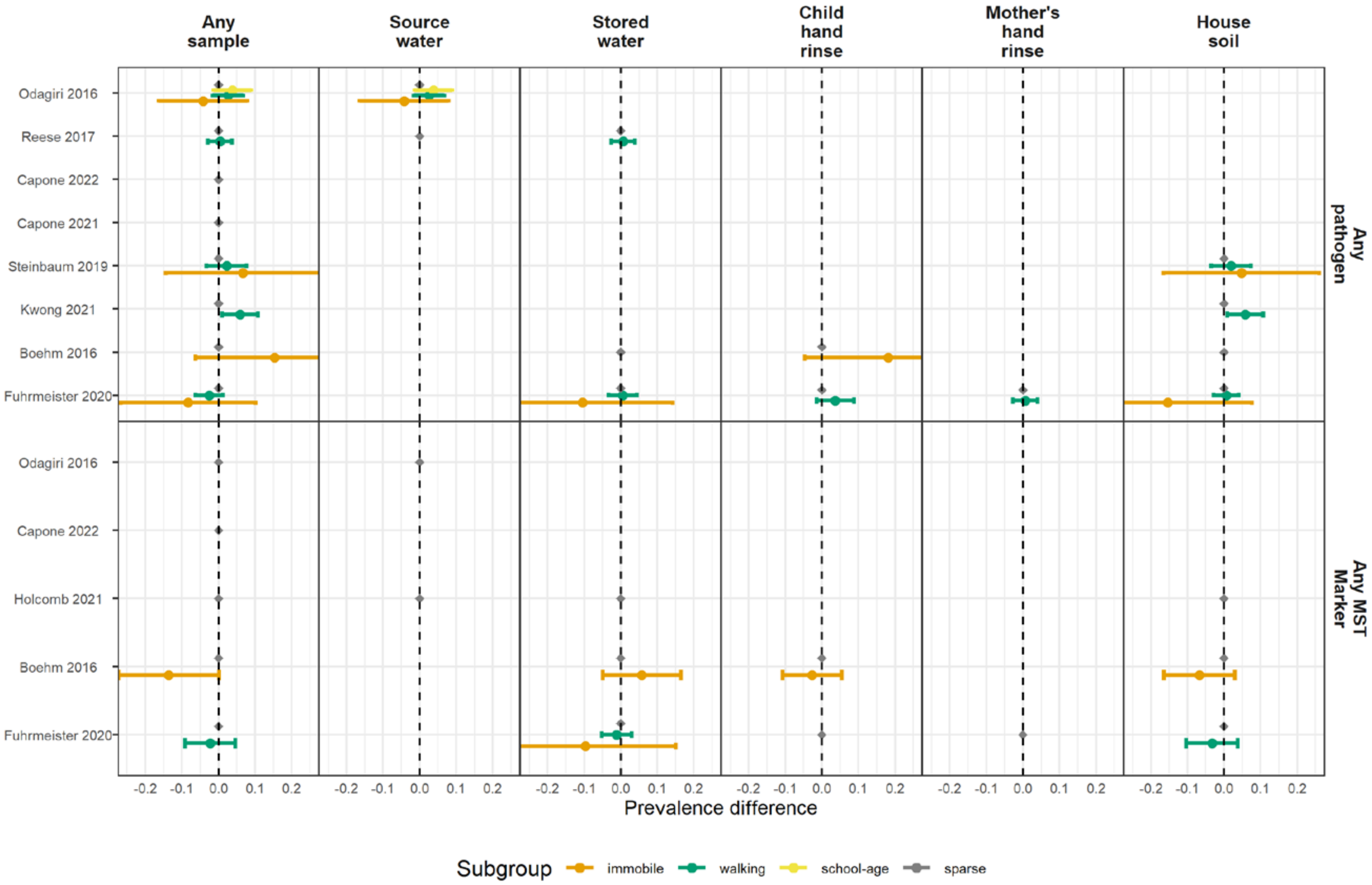

Figure S8. Forest plots of child diarrhoeal disease prevalence differences between environmental samples with and without any enteropathogen or any MST marker detected, stratified by child age. Grey points mark sparse age strata without estimated relative risks. Significant effect modification, as determined by the p-values on the regression model interaction term, is marked above points with asterisks (P < 0.05 = *, P < 0.01 = **, P < 0.001 = ***).

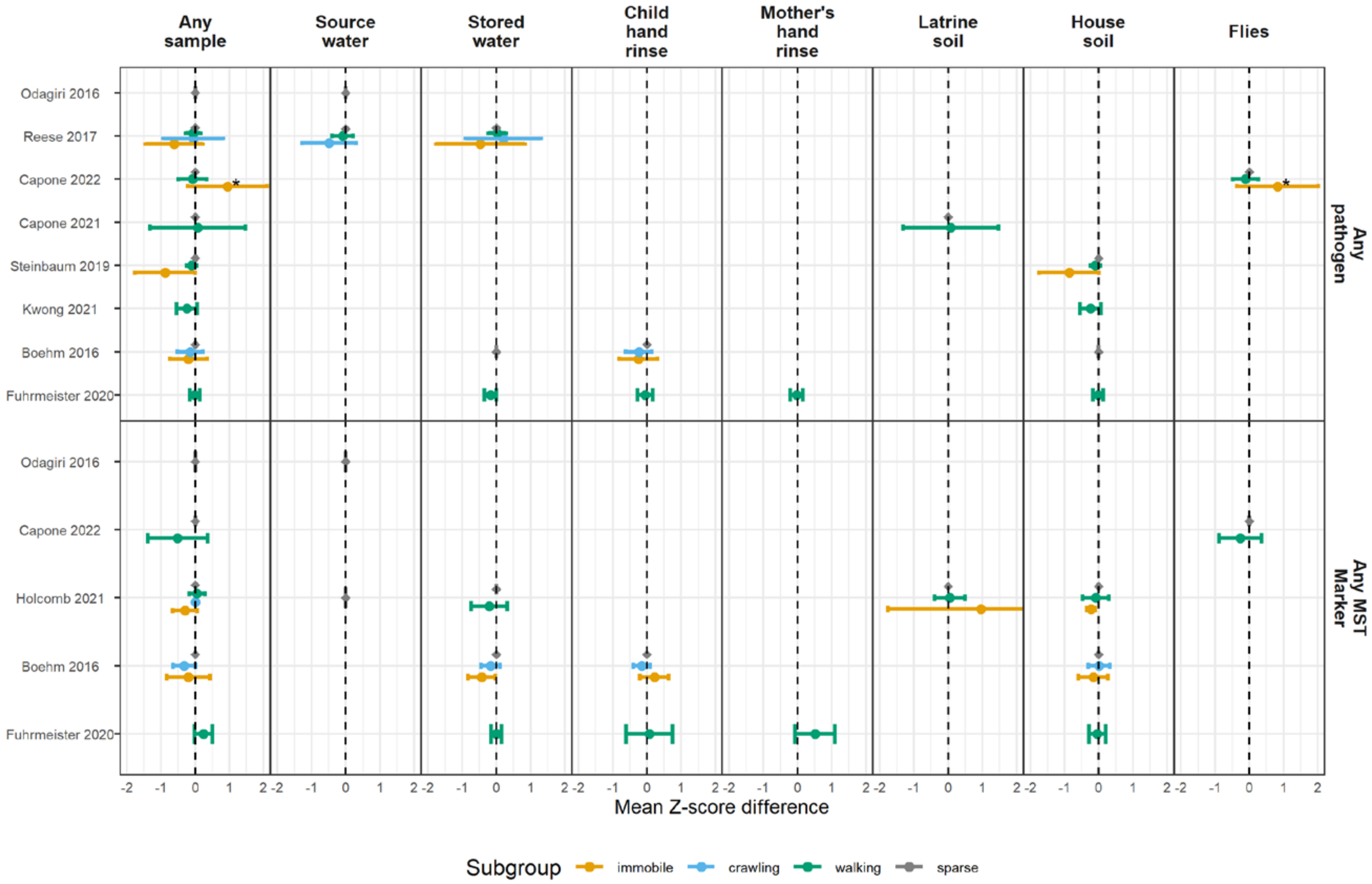

Figure S9. Forest plots of associations between any enteropathogen/any MST markers in different types of environmental samples and child height-for-age Z-score (HAZ), stratified by child age. Grey points mark sparse age strata without estimated mean differences. Significant effect modification, as determined by the p-values on the regression model interaction term, is marked above points with asterisks (P < 0.05 = *, P < 0.01 = **, P < 0.001 = ***).

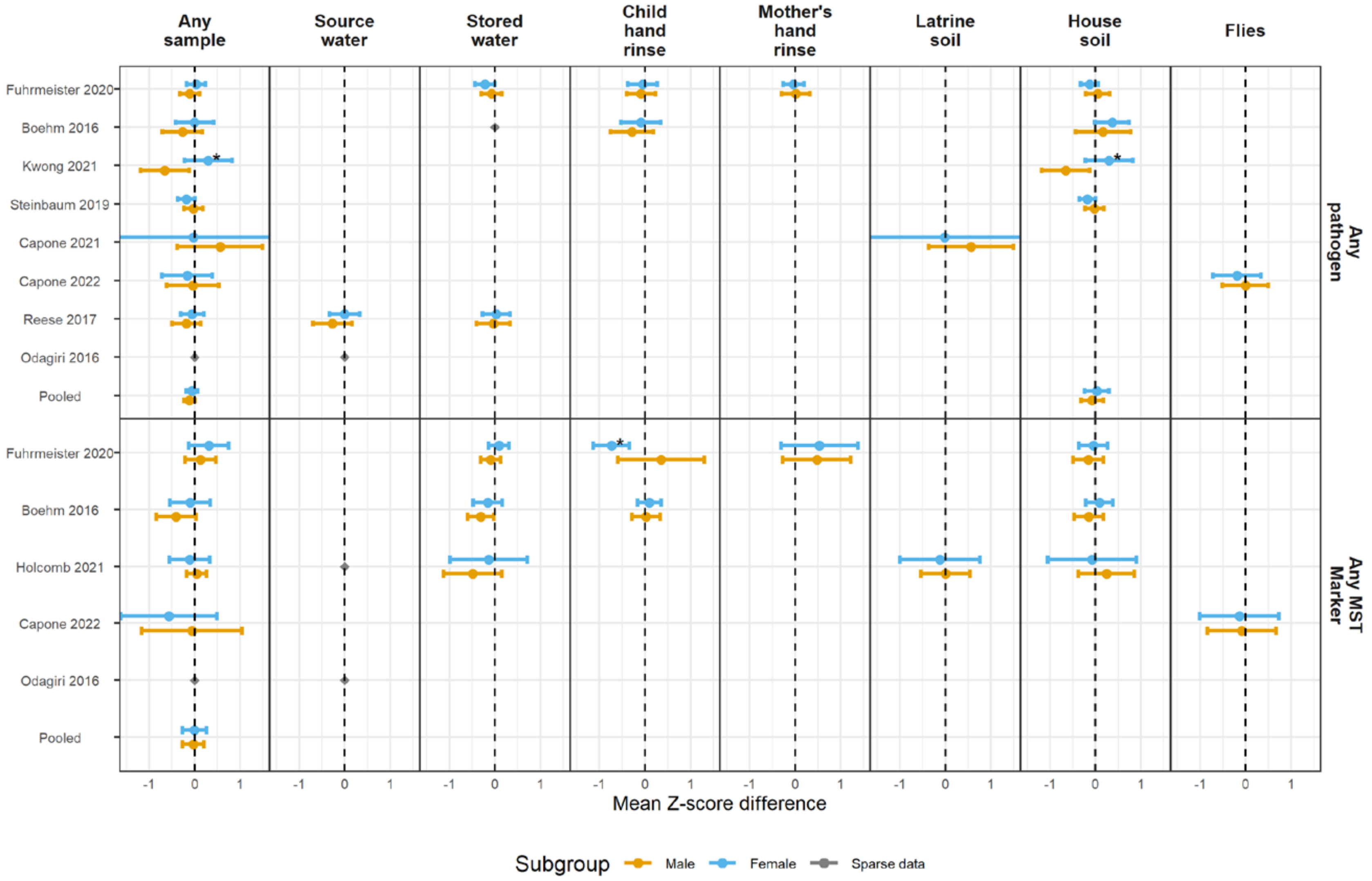

Figure S10. Forest plots of associations between any enteropathogen/any MST markers in different types of environmental samples and child height-for-age Z-scores (HAZ), stratified by child sex. Significant effect modification, as determined by the p-values on the regression model interaction term, is marked above points with asterisks (P < 0.05 = *, P < 0.01 = **, P < 0.001 = ***).

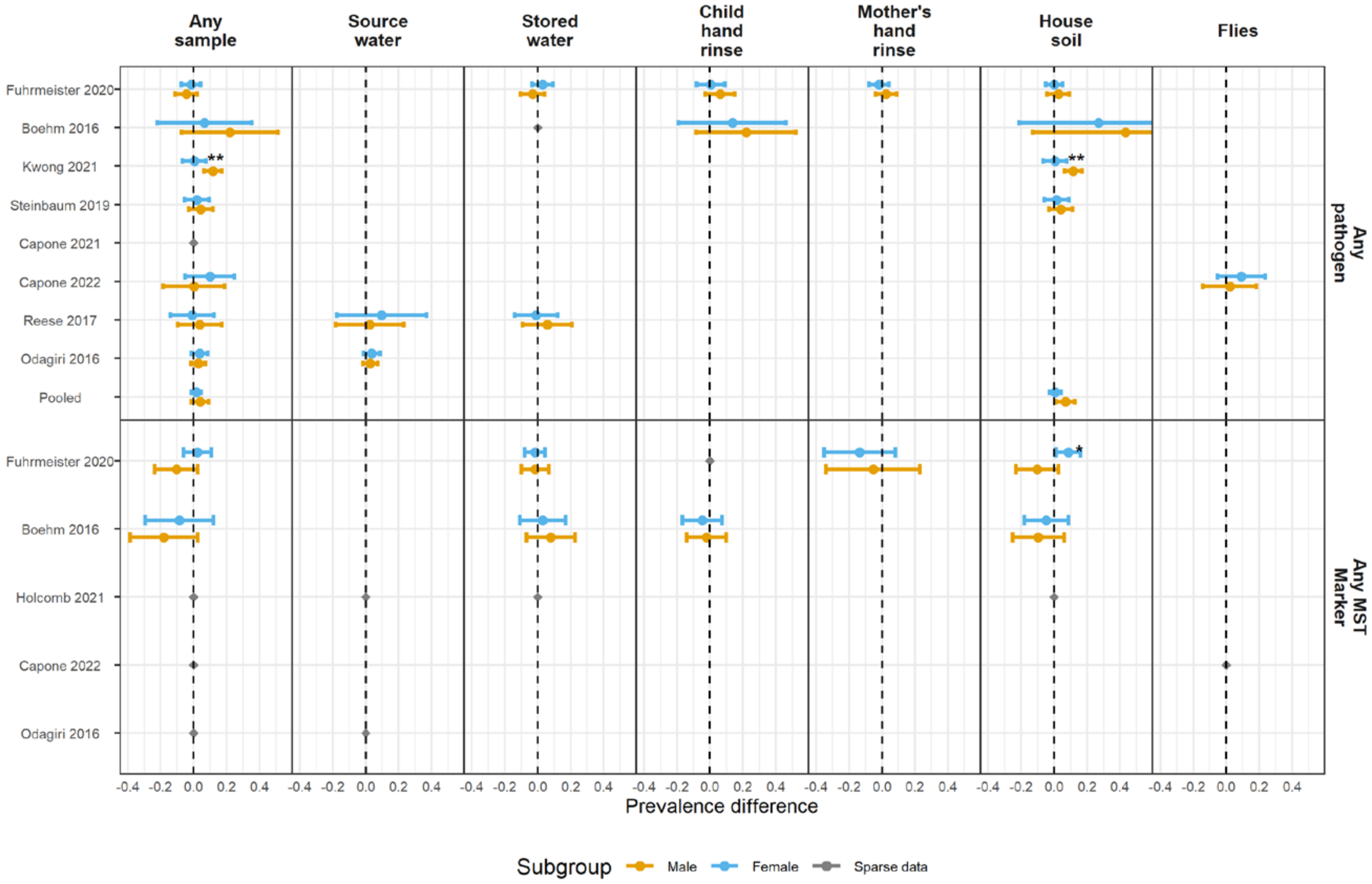

Figure S11. Forest plots of child diarrhoeal disease prevalence differences between environmental samples with and without any enteropathogen or any MST marker detected, stratified by child sex. Significant effect modification, as determined by the p-values on the regression model interaction term, is marked above points with asterisks (P < 0.05 = *, P < 0.01 = **, P < 0.001 = ***).

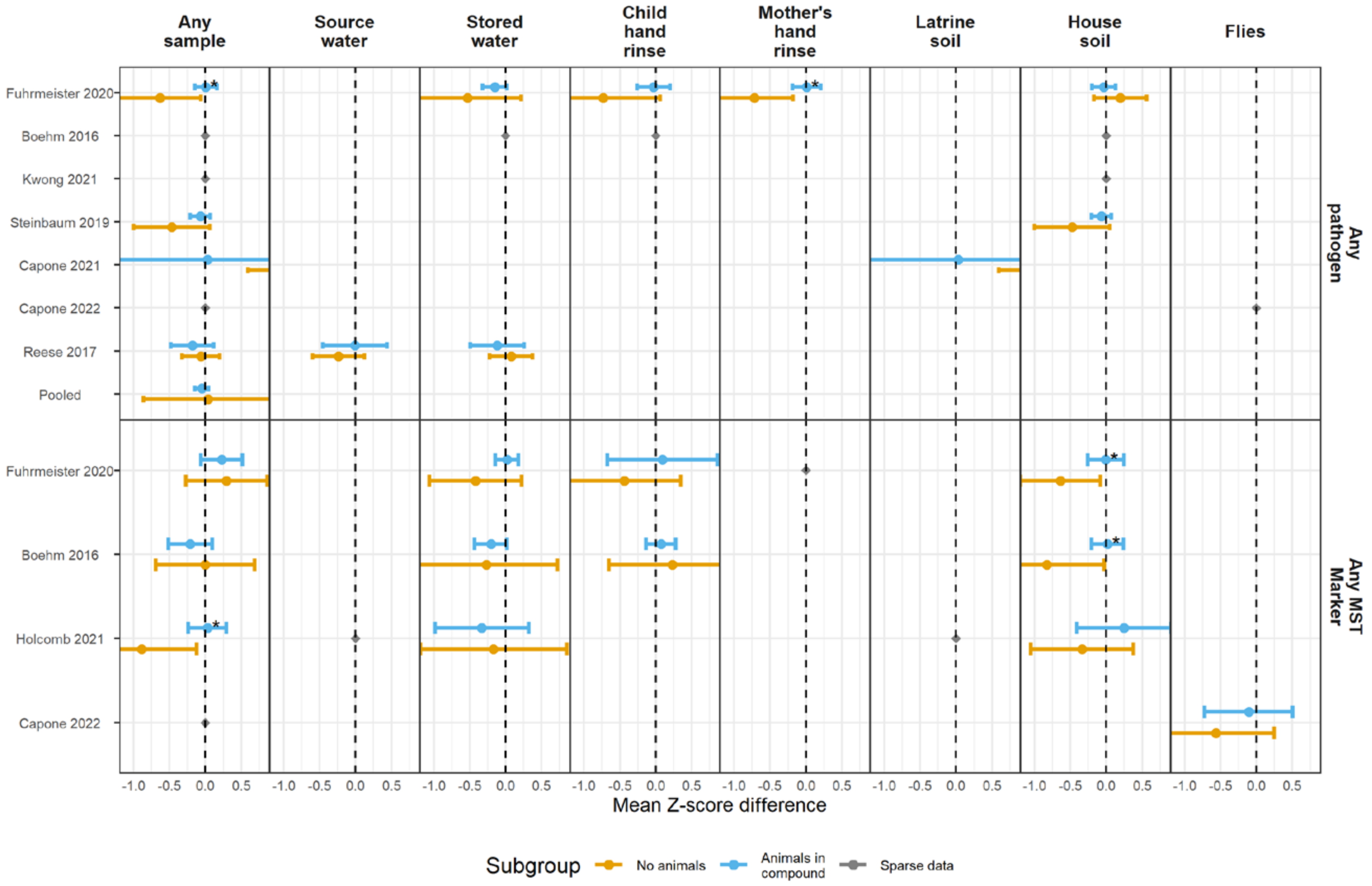

Figure S12. Forest plots of associations between any enteropathogen/any MST markers in different types of environmental samples and child height-for-age Z-scores (HAZ), stratified by whether any animals were present in the compound. Significant effect modification, as determined by the p-values on the regression model interaction term, is marked above points with asterisks (P < 0.05 = *, P < 0.01 = **, P < 0.001 = ***).

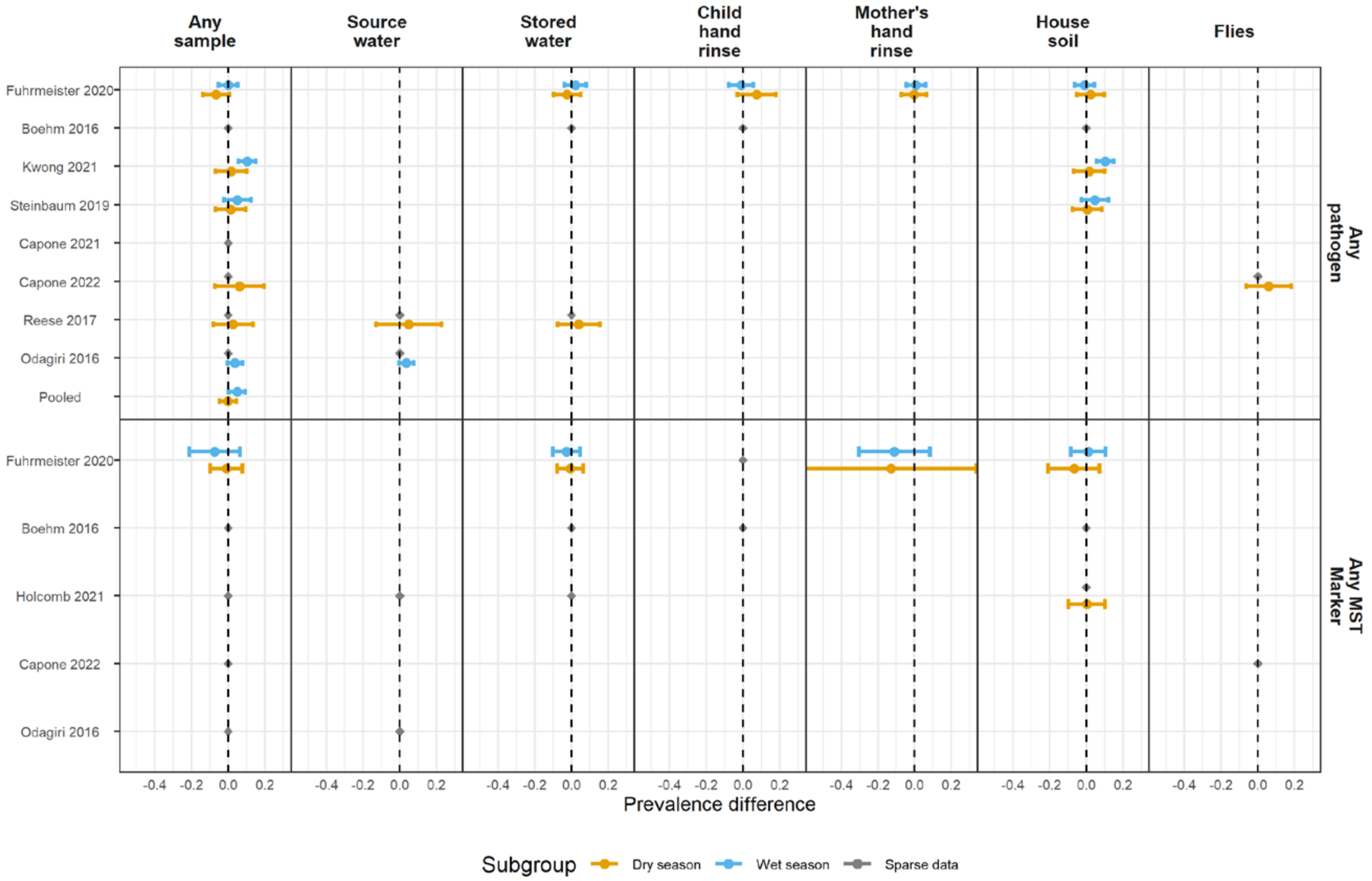

Figure S13. Forest plots of child diarrhoeal disease prevalence differences between environmental samples with and without any enteropathogen or any MST marker detected, stratified by whether the diarrhoeal disease occurred during the wet versus dry season (defined by the 6 months of highest average rainfall). Significant effect modification, as determined by the p-values on the regression model interaction term, is marked above points with asterisks (P < 0.05 = *, P < 0.01 = **, P < 0.001 = ***).

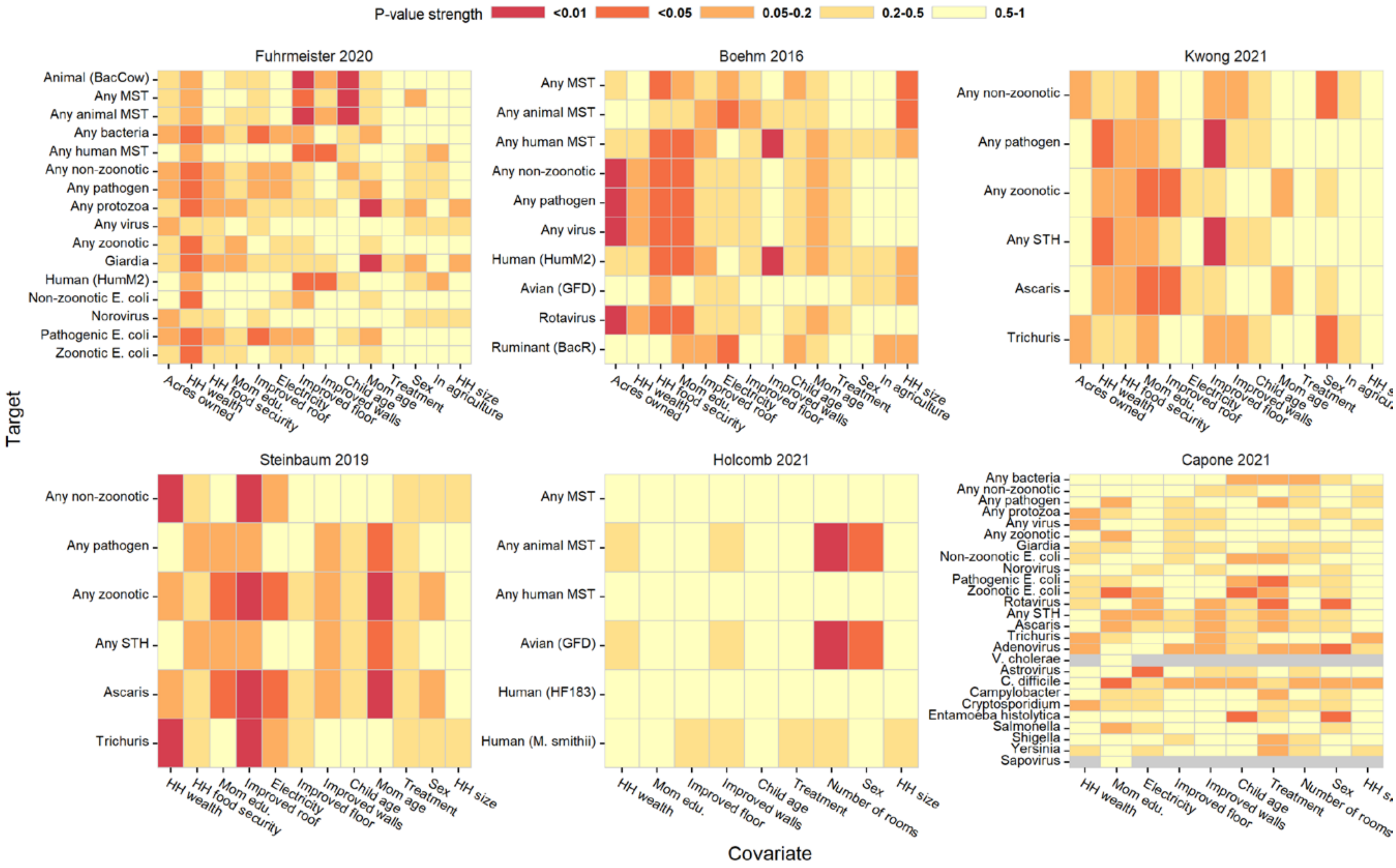
P-value strength
<0.01
<0.05
0.05-0.2
0.2-0.5
0.5-1
Fuhrmeister 2020
Boehm 2016
Kwong 2021
Steinbaum 2019
Holcomb 2021
Capone 2021
Target
Covariate
Animal (BacCow)
Any MST
Any animal MST
Any bacteria
Any human MST
Any non-zoonotic
Any pathogen
Any protozoa
Any virus
Any zoonotic
Giardia
Human (HumM2)
Non-zoonotic E. coli
Norovirus
Pathogenic E. coli
Zoonotic E. coli
Avian (GFD)
Rotavirus
Ruminant (BacR)
Any STH
Ascaris
Trichuris
Human (HF183)
Human (M. smithii)
Adenovirus
V. cholerae
Astrovirus
C. difficile
Campylobacter
Cryptosporidium
Entamoeba histolytica
Salmonella
Shigella
Yersinia
Sapovirus
Acres owned
HH wealth
HH food security
Mom edu.
Improved roof
Electricity
Improved floor
Improved walls
Child age
Mom age
Treatment
Sex
In agriculture
HH size
Number of rooms

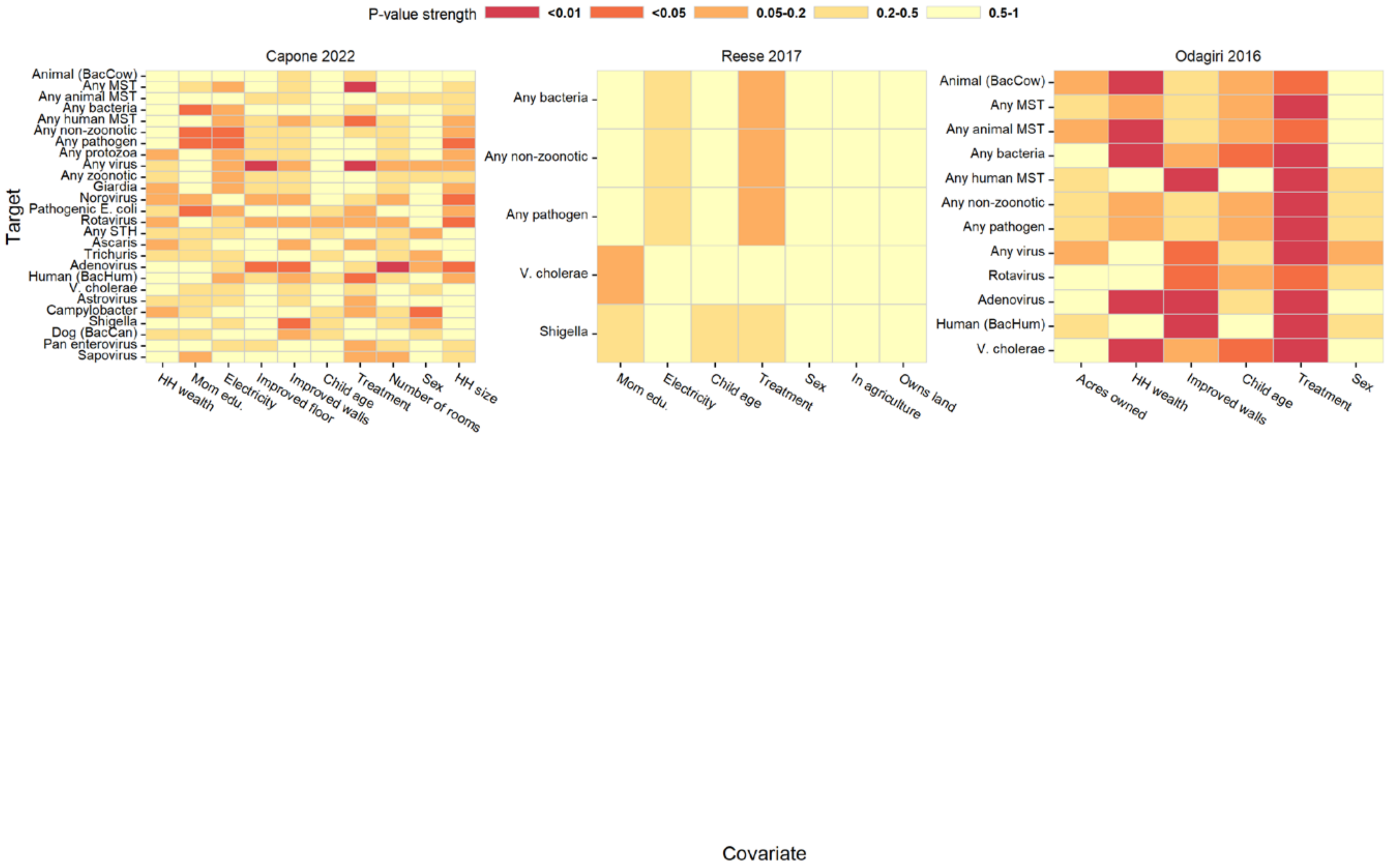

Figure S14. Study-specific associations between adjustment covariates (columns) and the presence of different enteropathogens and MST markers (rows) in aggregated environmental samples. Cells of the heatmaps are colored by P-values of bivariate likelihood ratio tests, and heatmaps are stratified by study.

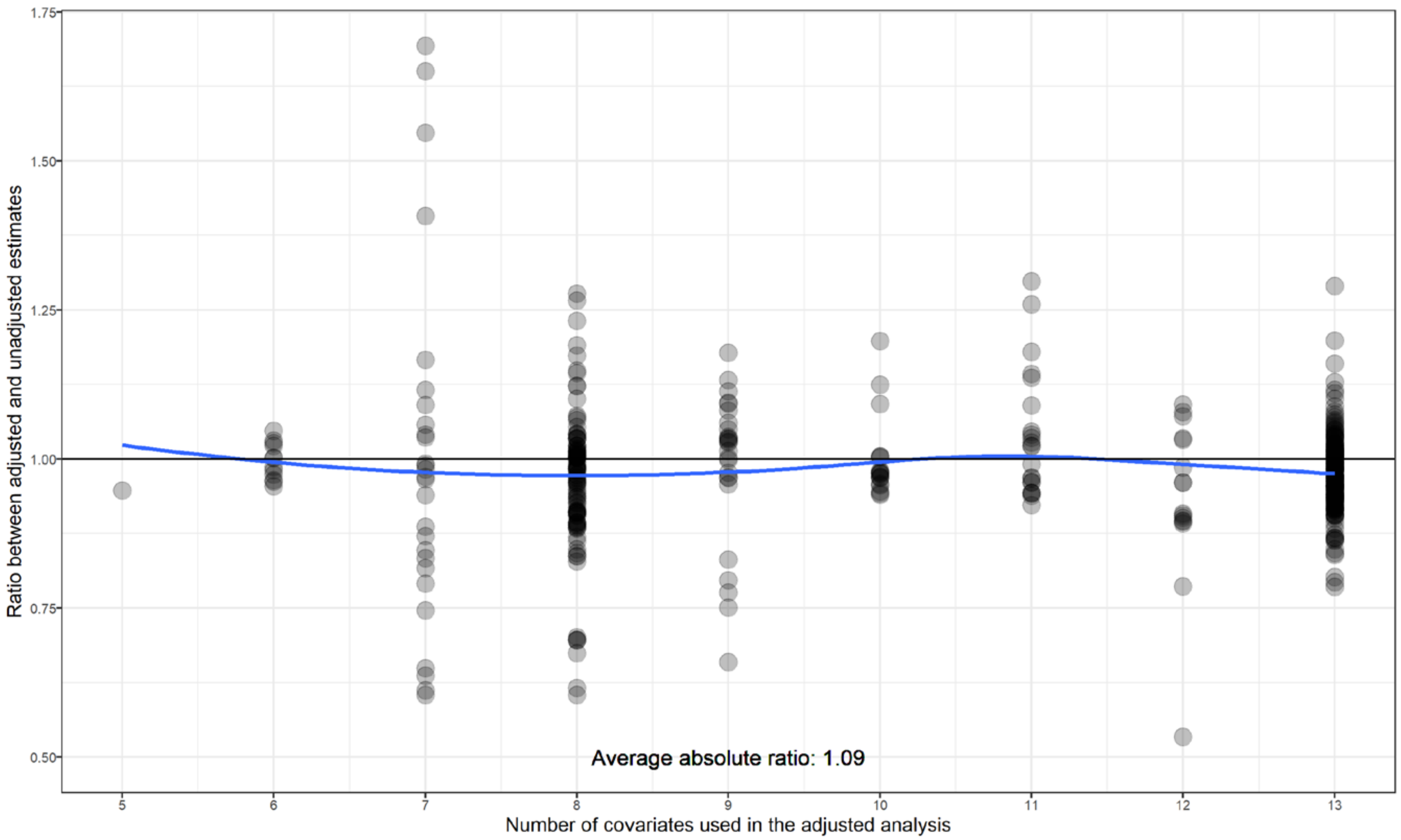

Figure S15. Comparison between associations estimated with and without adjusting for confounders for the binary diarrhoea and growth outcomes. Points mark the ratio of relative risks estimated using adjusted and unadjusted generalized linear models. The blue line shows the average ratio between adjusted estimates and unadjusted estimates, fitted using a cubic spline. The X-axis marks the number of covariates measured in the individual study.

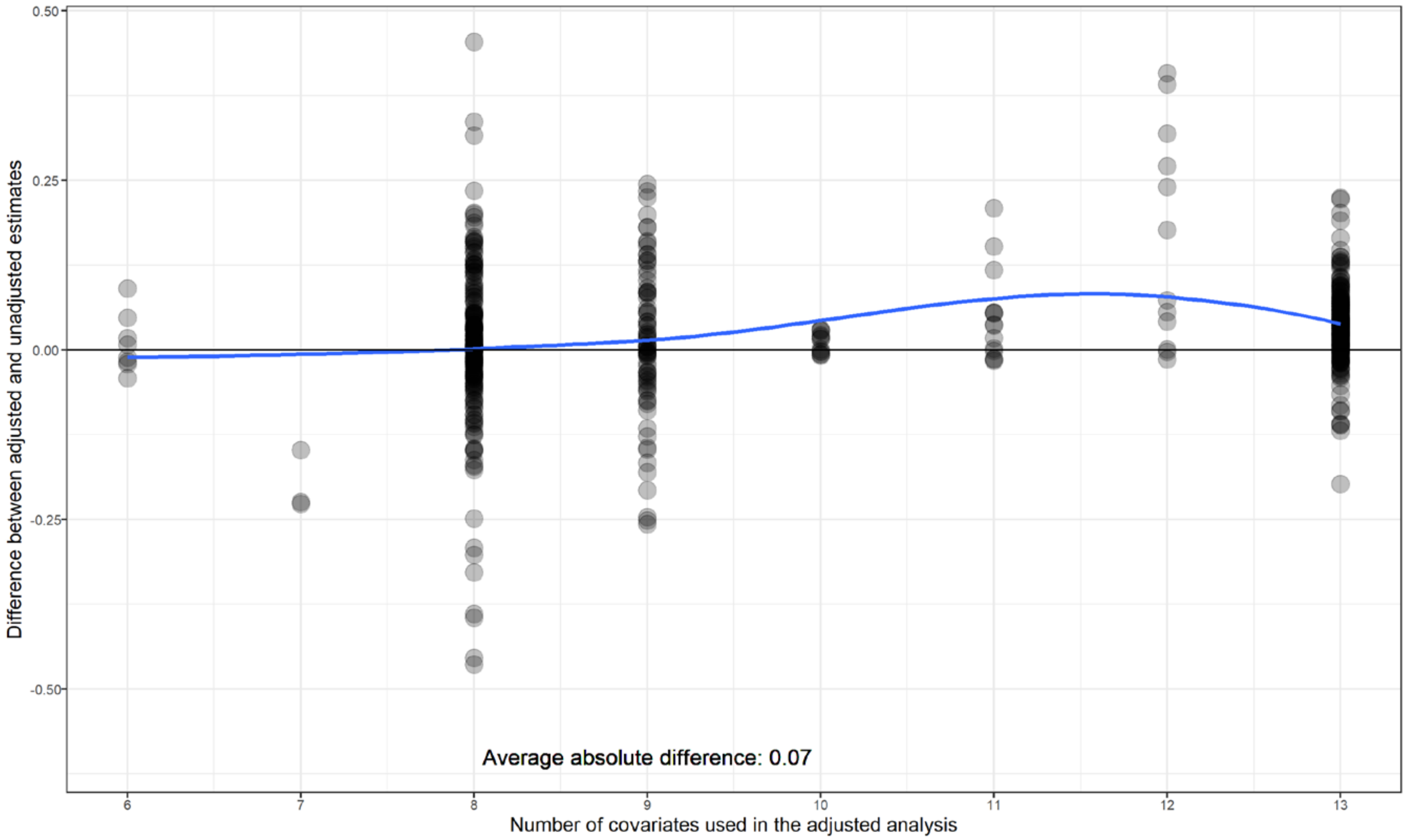

Figure S16. Comparison between associations estimated with and without adjusting for confounders for the continuous growth outcomes. Points mark the differences between mean differences estimated using adjusted and unadjusted generalized linear models. The blue line shows the average difference in differences between adjusted estimates and unadjusted estimates, fitted using a cubic spline. The X-axis marks the number of covariates measured in the individual study.

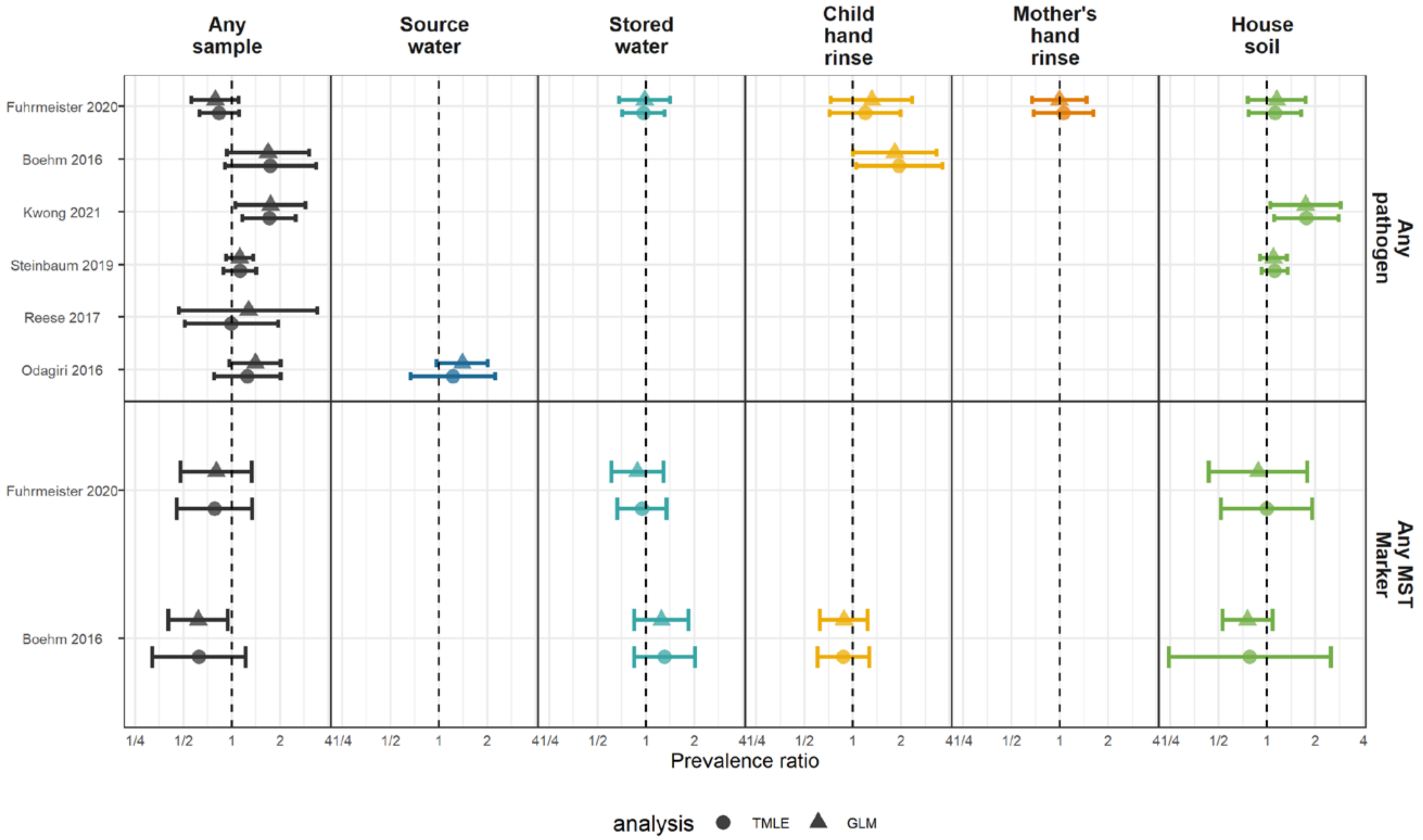

Figure S17. Comparison between associations estimated with generalized linear models (GLM) and machine-learning based targeted likelihood estimation models (TMLE) for the diarrhoea outcome.

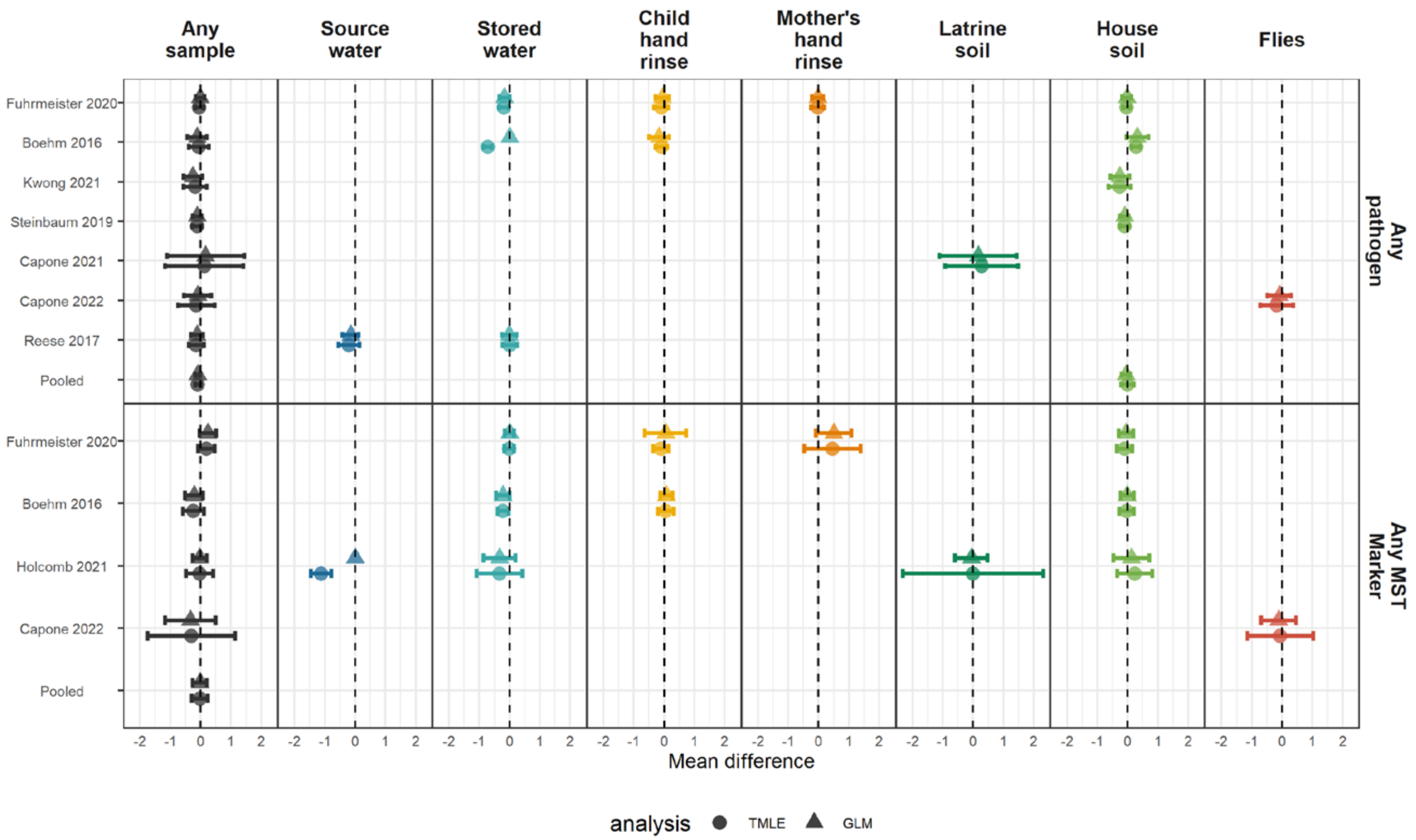

Figure S18. Comparison between associations estimated with generalized linear models (GLM) and machine-learning based targeted likelihood estimation models (TMLE) for the height-for-age Z-score (HAZ) outcome.

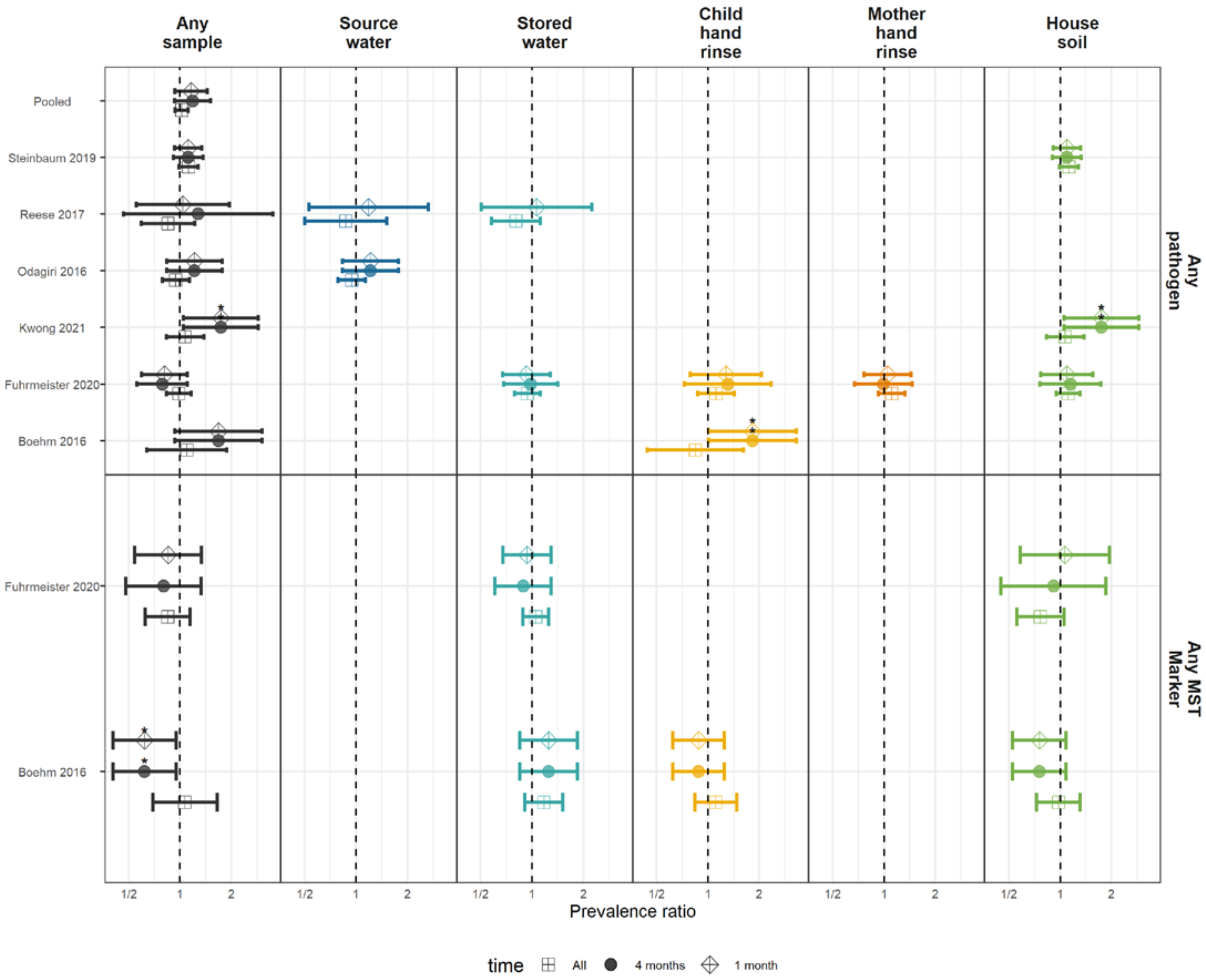

Figure S19. Comparison between associations estimated in the primary diarrhoea analysis (diarrhoeal disease occurring within 4 months after environmental sampling) with associations estimated only using diarrhoeal disease cases occurring within 1 month after environmental sampling or occurring at any time. Diarrhoea occurring at any time included all diarrhoeal cases, even cases occurring prior to environmental sampling, under the hypothesis that enteropathogen presence at one time is a surrogate variable for general environmental contamination.

SUPPLEMENTARY TABLES

Table S1. Systematic Review Search Terms by Database

Each row is search terms for each specific database, with columns showing search terms separated by domain for readability. In the database queries, the Study Design, WASH, Environmental Markers, child fecal markers, and the two child health outcome markers were separated by AND while the two health outcome columns were separated by OR (Diarrhea OR Child Growth).

Data-base	Study design	WASH	Environmental markers	Child fecal markers	Diarrhea	Child growth
**Pubmed**	((matched [tw]) OR (trial [tw]) OR (RCT [tw]) OR (experiment [tw]) OR (intervention [tw]) OR (randomized [tw]) OR (randomised [tw]) OR (quasi-randomized [tw]) OR (quasi-randomised [tw]) OR (quasi-experimental [tw]) OR (pseudo-randomized [tw]) OR (pseudo-randomised [tw]) OR (“non-randomized controlled trials as topic” [mh])	(Water [tw]) OR (Sanitation [tw]) OR (Hygiene [tw]) OR (Handwashing [tw]) OR (WSH [tw]) OR (“Sanitation” [mh]) OR (“Water Supply” [mh]) OR (“Drainage, Sanitary” [mh]) OR (Sanitary Drainage [tw]) OR (“Toilet Facilities” [mh]) OR (“Drinking Water” [mh]) OR (“Hand Hygiene” [mh]) OR (“Water Purification” [mh]) OR (“Waste Water” [mh]) OR (disinfect* [tw])	(molecular source tracking [tw]) OR (microbial source tracking [tw]) OR (microbial transmission [tw]) OR (diarrheal pathogen [tw]) OR (diarrheal pathogens [tw]) OR (diarrhoeal pathogen [tw]) OR (diarrhoeal pathogens [tw]) OR (fecal-oral [tw]) OR (faecal-oral [tw]) OR (enteric pathogen [tw]) OR (enteric pathogens [tw]) OR (ruminant* [tw]) OR (avian* [tw]) OR (“Feces” [mh]) OR (Feces [tw]) OR (Faeces [tw]) OR (Fecal [tw]) OR (Faecal [tw]) OR (Fecally [tw]) OR (Faecally [tw]) OR	(Enteric infection* [tw]) OR (Soil-transmitted helminth* [tw]) OR (Protozoan* [tw]) OR (Seroconversion [tw]) OR (Fecal microbio* [tw]) OR (Faecal microbio* [tw]) OR (Fecal biomarker* [tw]) OR (Faecal biomarker* [tw]) OR (“Intestinal Diseases, Parasitic/epidemiology” [mh]) OR (“Seroconversion” [mh]) OR (Seroconversion [tw]) OR (“Enteritis/epidemiology” [mh]) OR (“Helminthiasis/complications” [mh]) OR (Helminthiasis [tw]) OR (Helminthiases)OR (“Helminthiasis/epidemiology” [mh]) OR (“Helminthiasis/prevention and control” [mh]) OR (Intestinal infection* [tw]) OR (Viral infection* [tw]) OR	(Diarrh* [tw]) OR (Dysentery [tw]) OR (“Diarrhea/epidemiology” [mh]) OR (“Diarrhea/etiology” [mh]) OR (“Diarrhea/prevention and control“ [mh]) OR (“Diarrhea, Infantile” [mh]) OR (“Dysentery“ [mh]) OR (Bacterial infection* [tw]) OR (Parasite infection* [tw]) OR (Parasitic infection* [tw]) OR (Helminth infection* [tw]) OR (Fecal sampling [tw]) OR (Faecal sampling [tw]) OR OR (Stool sampling [tw]) OR (Stool collection [tw])	(Child growth faltering [tw]) OR (Growth faltering [tw])OR (Child development [tw]) OR (Length-for-age [tw]) OR (Height-for-age [tw]) OR (Weight-for-age [tw]) OR (Head circumference [tw]) OR (Waist circumference [tw]) OR (Stunt* [tw]) OR (Wasting [tw]) OR (Wasted [tw]) OR (Linear growth [tw]) OR (Anthropometric measurement* [tw]) OR (Maln* [tw]) OR (Undernourish* [tw]) OR (Undernutrition [tw]) OR (Underweight [tw]) OR (“Growth Disorders” [mh]) OR (Growth Disorders [tw]) OR (“Child nutrition disorders” [mh]) OR (Child nutrition disorder* [tw]) OR (“Malnutrition” [mh]) OR (“Wasting Syndrome” [mh]) OR (Wasting syndrome [tw]) OR (“Thinness” [mh]) OR (Thinness [tw]) OR (Growth velocity [tw])
**Embase**	(“matched”:ab,ti,kw OR “trial”:ab,ti,kw OR “RCT”:ab,ti,kw OR “experiment”:ab,ti,kw OR “intervention”:ab,ti,kw OR “randomized”:ab,ti,kw OR “randomised”:ab,ti,kw OR “quasi-randomized”:ab,ti,kw OR “quasi-	“Water”:ab,ti,kw OR “Sanitation”:ab,ti,kw OR “Hygiene”:ab,ti,kw OR “Piped water”:ab,ti,kw OR “Handwashing”:ab,ti,kw OR “WSH”:ab,ti,kw OR “Sanitation”/exp OR “Water	“molecular source tracking”:ab,ti,kw OR “microbial source tracking”:ab,ti,kw OR “microbial transmission”:ab,ti,kw OR “diarrh?eal pathogen?”:ab,ti,kw OR “f?ecal-oral”:ab,ti,kw OR “enteric pathogen?”:ab,ti,kw OR “ruminant*”:ab,ti,kw OR “avian*”:ab,ti,kw OR “feces”/exp OR “f?ecal”:ab,ti,kw OR “f?eces”:ab,ti,kw OR “f?ecally”:ab,ti,kw OR “environmental exposure”/exp	“Enteric infection?”:ab,ti,kw OR “Soil-transmitted helminth?”:ab,ti,kw OR “Protozoan?”:ab,ti,kw OR “Seroconversion”:ab,ti,kw OR “F?ecal microbio*”:ab,ti,kw OR “F?ecal biomarkers”:ab,ti,kw OR “Seroconversion”/exp OR “Seroconversion”:ab,ti,kw OR “Enteritis”/exp/dm_ep OR “Helminthiasis”/exp/dm_co OR “Helminthias?s”:ab,ti,kw OR “Helminthiasis” /exp/dm_ep OR “Helminthiasis”/exp/dm_pc OR “Intestinal infection?”:ab,ti,kw OR “Viral infection?”:ab,ti,kw OR	“Diarrh*”:ab,ti,kw OR “Dysentery”:ab,ti,kw OR “Diarrhea”/exp/dm_ep OR “Diarrhea”/exp/dm_et OR “Diarrhea”/exp/dm_pc OR “Feces”/exp OR “Diarrhea, Infantile”/exp OR “Dysentery”/exp “Bacterial infection?”:ab,ti,kw OR	“Child growth”:ab,ti,kw OR “Growth faltering”:ab,ti,kw OR “Child development”:ab,ti,kw OR “Length-for-age”:ab,ti,kw OR “Height-for-age”:ab,ti,kw OR “Weight-for-age”:ab,ti,kw OR “Head circumference”:ab,ti,kw OR “Waist circumference”:ab,ti,kw OR “Stunt*”:ab,ti,kw OR “Wasting”:ab,ti,kw OR “Wasted”:ab,ti,kw OR “Linear growth”:ab,ti,kw OR “Anthropometric measurement?”:ab,ti,kw OR “Maln*”:ab,ti,kw OR “Undernourish*”:ab,ti,kw OR

	randomised":ab,ti, kw OR "quasi-experimental":ab,ti,kw OR "pseudo-randomized":ab,ti, kw OR "pseudo-randomised":ab,ti, kw OR "randomized controlled trial"/exp OR "experimental study"/exp OR "controlled study"/exp OR "quasi experimental study"/exp)	Supply"/exp OR "Drainage, Sanitary"/exp OR "Sanitary Drainage":ab,ti,kw OR "Toilet Facilities"/exp OR "Drinking Water"/exp OR "Hand Hygiene"/exp OR "Water Purification"/exp OR "Waste Water"/exp OR "disinfect*":ab,ti, kw OR "hand washing"/exp OR "water quality"/exp OR "Hygiene"/exp	OR "contamination"/exp OR "feces analysis"/exp OR "marker gene"/exp OR "bacteroidales"/exp		"Parasite infection?":ab,ti,kw OR "Parasitic infection?":ab,ti,kw OR "Helminth infection?":ab,ti,kw OR "F?ecal sampling":ab,ti,kw OR "Stool sampling":ab,ti,kw OR "Stool collection":ab,ti,kw OR "gastrointestinal disease"/exp OR "intestine infection"/exp	"Undernutrition":ab,ti,kw OR "Underweight":ab,ti,kw OR "Growth Disorders"/exp OR "Growth Disorder?":ab,ti,kw OR "Child nutrition disorder"/exp OR "Child nutrition disorder?":ab,ti,kw OR "Malnutrition"/exp OR "Wasting Syndrome"/exp OR "Wasting syndrome":ab,ti,kw OR "Thinness"/exp OR "Thinness":ab,ti,kw OR "Growth velocity":ab,ti,kw OR "Child nutrition"/exp OR "Child development"/exp OR "nutritional status"/exp OR "infant nutrition disorders"/exp
**CAB Global health**	Ab:"matched" OR ab:"trial" OR ab:"RCT" OR ab:"experiment" OR ab:"intervention" OR ab:"randomized" OR ab:"quasi-randomized" OR ab:"quasi-experimental" OR ab:"impact" OR ab:"pseudo-randomized" OR ab:"randomised" OR ab:"quasi-randomised" OR ab:"pseudo-randomised"title:"matched" OR title:"trial" OR title:"RCT" OR title:"experiment" OR title:"intervention" OR title:"randomized" OR title:"quasi-randomized" OR title:"quasi-experimental" OR title:"impact" OR	ab:"Water" OR ab:"Sanitation" OR ab:"Hygiene" OR ab:"Hand washing" OR ab:"WSH" OR ab:"Sanitary drainage" OR ab:"Toilet Facilities" OR ab:"Hand Hygiene" OR ab:"disinfect*" OR title:"Water" OR title:"Sanitation" OR title:"Hygiene" OR title:"Hand washing" OR title:"WSH" OR title:"Sanitary drainage" OR title:"Toilet Facilities" OR title:"Hand Hygiene" OR title:"disinfect*" OR de:"water quality" OR id:"chlorinated drinking water" OR id:"water composition and	ab:"molecular source tracking" OR ab:"microbial source tracking" OR ab:"microbial transmission" OR ab:"diarrheal pathogen" OR ab:"diarrheal pathogen" OR ab:"diarrheal pathogens" OR ab:"diarrhoeal pathogens" OR ab:"diarrh* pathogen" OR ab:"fecal-oral" OR ab:"faecal-oral" OR ab:"enteric pathogens" OR ab:"ruminant*" OR ab:"avian*" OR ab:"Feces" OR ab:"Faeces" OR ab:"Fecal" OR ab:"Faecal" OR ab:"Fecally" OR ab:"Faecally" OR title:"molecular source tracking" OR title:"microbial source tracking" OR title:"microbial transmission" OR title:"diarrheal pathogen" OR title:"diarrhoeal pathogen" OR title:"diarrheal pathogens" OR title:"diarrhoeal pathogens" OR title:"fecal-oral" OR title:"faecal-oral" OR title:"enteric pathogens" OR title:"ruminant*" OR title:"avian*" OR title:"Feces" OR title:"Faeces" OR title:"Fecal" OR title:"Faecal" OR title:"Fecally" OR title:"Faecally" OR de:"faeces" OR id:"feces" OR de:"biological indicators" OR de:"contamination" OR	ab:"Enteric infection*" OR ab:"Soil-transmitted helminth*" OR ab:"Protozoan*" OR ab:"Fecal microbio*" OR ab:"Faecal microbio*" OR ab:"Fecal biomarker*" OR ab:"Faecal biomarker*" OR ab:"Intestinal Disease*" OR ab:"Seroconversion" OR ab:"Enteritis" OR ab:"Helminthiasis" OR ab:"Intestinal infection*" OR ab:"Viral infection*" OR ab:"Bacterial infection*" OR ab:"Parasite infection*" OR ab:"Parasitic infection*" OR ab:"Helminth infection*" OR ab:"Fecal sampling" OR ab:"Faecal sampling" OR ab:"Stool examination" OR ab:"Stool sampling" OR ab:"Stool collection" OR title:"Enteric infection*" OR title:"Soil-transmitted helminth*" OR title:"Protozoan*" OR title:"Seroconversion" OR title:"Fecal microbio*" OR title:"Faecal microbio*" OR title:"Fecal biomarker*" OR title:"Faecal biomarker*" OR title:"Intestinal Disease*" OR title:"Enteritis" OR title:"Helminthiasis" OR title:"Intestinal infection*" OR title:"Viral infection*" OR title:"Bacterial infection*" OR title:"Parasite infection*" OR title:"Parasitic infection*" OR title:"Helminth infection*" OR title:"Fecal sampling" OR title:"Faecal sampling"	ab:"diarrh*" OR ab:"Dysentery" OR title:"diarrh*" OR title:"Dysentery" OR id:"diarrhea" OR de:"diarrhea" OR title:"Stool examination" OR title:"Stool sampling" OR title:"Stool collection" OR de:"faecal examination" OR de:"helminthoses" OR de:"helminths" OR de:"parasites" OR de:"parasitoses" OR de:"intestinal microorganisms" OR de:"diarrhoeagenic E. coli" OR de:"Diarrhoeagenic Escherichia coli"	ab:"Child growth" OR ab:"Growth faltering" OR ab:"Child development" OR ab:"Length-for-age" OR ab:"Height-for-age" OR ab:"Weight-for-age" OR ab:"Head circumference" OR ab:"Waist circumference" OR ab:"Stunt*" OR ab:"Wasting" OR ab:"Wasted" OR ab:"Linear growth" OR ab:"Anthropometric measurement*" OR ab:"Maln*" OR ab:"Undernourish*" OR ab:"Undernutrition" OR ab:"Underweight" OR ab:"Growth Disorder*" OR ab:"child nutrition disorder*" OR ab:"Malnutrition" OR ab:"Wasting Syndrome" OR ab:"Thinness" OR ab:"Growth velocity" OR ab:"Acute malnutrition" OR title:"Child growth" OR title:"Growth faltering" OR title:"Child development" OR title:"Length-for-age" OR title:"Height-for-age" OR title:"Weight-for-age" OR title:"Head circumference" OR title:"Waist circumference" OR title:"Stunt*" OR title:"Wasting" OR title:"Wasted" OR title:"Linear growth" OR title:"Anthropometric measurement*" OR title:"Maln*" OR title:"Undernourish*" OR title:"Undernutrition" OR title:"Underweight" OR title:"Growth Disorder*" OR title:"Child nutrition disorder*" OR title:"Malnutrition" OR title:"Wasting Syndrome" OR title:"Thinness" OR title:"Growth

	title:"pseudo-randomized" OR title:"randomised" OR title:"quasi-randomised" OR title:"pseudo-randomised"OR de:"randomized controlled trials" OR de:"experimental design" OR de:"health impact assessment"	quality" OR de:"chlorination" OR de:"drinking water" OR de:"latrine" OR de:"water supply" OR de:"Water treatment"	de:"indicator species" OR de:"microbial contamination" OR de:"marker genes" OR de:"microbiological techniques"			velocity" OR title:"Acute malnutrition"OR de:"child development" OR de:"growth" OR de:"arm circumference" OR de:"child development" OR id:"mid-upper-arm circumference" OR de:"anthropometric dimensions"
**Web of Science**	TS=(("matched") OR ("trial") OR ("RCT") OR ("experiment") OR ("intervention") OR ("randomized") OR ("randomised") OR ("quasi-randomized") OR ("quasi-randomised") OR ("quasi-experimental") OR ("pseudo-randomized") OR ("pseudo-randomised"))	TS=(("Water") OR ("Sanitation") OR ("Hygiene") OR ("Handwashing") OR ("WSH") OR ("Sanitary Drainage") OR ("disinfect*"))	TS=(("molecular source tracking") OR ("microbial source tracking") OR ("microbial transmission") OR ("diarrh$eal pathogen") OR ("diarrh$eal pathogens") OR ("f$ecal-oral") OR ("enteric pathogen$") OR ("ruminant*") OR ("avian*") OR ("f$ecal") OR ("f$eces") OR ("f$ecally"))	TS=(("Enteric infection$") OR ("Soil-transmitted helminth$") OR ("Protozoan$") OR ("Seroconversion") OR ("F$ecal microbio*") OR ("F$ecal biomarker") OR ("F$ecal biomarkers") OR ("Seroconversion") OR ("Helminthias$s") OR ("Intestinal infection$") OR ("Viral infection$") OR ("Bacterial infection$") OR ("Parasite infection$") OR ("Parasitic infection$") OR ("Helminth infection$") OR ("F$ecal sampling")	OR ("Diarrh*") OR ("Dysentery") OR ("Stool sampling") OR ("Stool collection") OR ("Enteritis")	OR ("Child growth") OR ("Growth faltering")OR ("Child development") OR ("Length-for-age") OR ("Height-for-age") OR ("Weight-for-age") OR ("Head circumference") OR ("Waist circumference") OR ("Stunt*") OR ("Wasting") OR ("Wasted") OR ("Linear growth") OR ("Anthropometric measurement$") OR ("Maln*") OR ("Undernourish*") OR ("Undernutrition") OR ("Underweight") OR ("Growth Disorders") OR ("Child nutrition disorder$") OR ("Wasting syndrome") OR ("Thinness") OR ("Growth velocity"))
**Agricultural & Environmental Science Database**	AB,TI,IF("matched") OR AB,TI,IF("trial") OR AB,TI,IF("RCT") OR AB,TI,IF("experiment") OR AB,TI,IF("intervention") OR AB,TI,IF("randomized") OR AB,TI,IF("randomised") OR AB,TI,IF("quasi-randomized") OR AB,TI,IF("quasi-randomised") OR AB,TI,IF("quasi-experimental") OR AB,TI,IF("pseudo-	AB,TI,IF("Water") OR AB,TI,IF("Sanitation") OR AB,TI,IF("Hygiene") OR AB,TI,IF("Handwashing") OR AB,TI,IF("WSH") OR AB,TI,IF("Sanitary Drainage") OR AB,TI,IF("disinfect*") OR SU("Sanitation") OR SU("Water treatment") OR SU("Hygiene") OR SU("Drinking water") OR	AB,TI,IF("molecular source tracking") OR AB,TI,IF("microbial source tracking") OR AB,TI,IF("microbial transmission") OR AB,TI,IF("diarrheal pathogen?") OR AB,TI,IF("diarrhoeal pathogen?") OR AB,TI,IF("enteric pathogen?") OR AB,TI,IF("ruminant*") OR AB,TI,IF("avian*") OR AB,TI,IF("f?ec????") OR SU("Contamination") OR SU("Environmental assessment") OR SU("environmental conditions")	AB,TI,IF("Enteritis") OR AB,TI,IF("Enteric infection?") OR AB,TI,IF("Soil-transmitted helminth?") OR AB,TI,IF("Protozoan?") OR AB,TI,IF("Seroconversion") OR AB,TI,IF("Fecal microbiological") OR AB,TI,IF("Fecal microbiology") OR AB,TI,IF("Fecal microbiota") OR AB,TI,IF("Faecal microbiota") OR AB,TI,IF("Faecal microbiological") OR AB,TI,IF("Faecal microbiology") OR AB,TI,IF("Fecal biomarker?") OR AB,TI,IF("Faecal biomarker") OR AB,TI,IF("Faecal biomarkers") OR AB,TI,IF("Helminthias?s") OR AB,TI,IF("Viral infection") OR AB,TI,IF("Viral infections") OR AB,TI,IF("Bacterial infection") OR AB,TI,IF("Bacterial infections") OR AB,TI,IF("Parasite infection") OR AB,TI,IF("Parasite infections") OR AB,TI,IF("Parasitic infection") OR AB,TI,IF("Parasitic infections") OR AB,TI,IF("Helminth infection") OR AB,TI,IF("Helminth infections") OR	AB,TI,IF("Diarrh*") OR AB,TI,IF("Dysentery") OR SU("Diarrhea") OR AB,TI,IF("Intestinal infection") OR AB,TI,IF("Intestinal infections")	AB,TI,IF("Child growth") OR AB,TI,IF("Growth faltering") OR AB,TI,IF("Child development") OR AB,TI,IF("Length-for-age") OR AB,TI,IF("Height-for-age") OR AB,TI,IF("Weight-for-age") OR AB,TI,IF("Head circumference") OR AB,TI,IF("Waist circumference") OR AB,TI,IF("Stunt*") OR AB,TI,IF("Wasting") OR AB,TI,IF("Wasted") OR AB,TI,IF("Linear growth") OR AB,TI,IF("Anthropometric measurement") OR AB,TI,IF("Anthropometric measurements") OR AB,TI,IF("Maln*") OR AB,TI,IF("Undernourish*") OR AB,TI,IF("Undernutrition") OR AB,TI,IF("Underweight") OR AB,TI,IF("Growth Disorder") OR AB,TI,IF("Growth Disorders") OR AB,TI,IF("Child nutrition disorder") OR

	randomized”) OR AB,TI,IF(“pseudo-randomised”) OR SU(“Randomized controlled trials”) OR SU(“Randomized”)	SU(“Groundwater”)		AB,TI,IF(“Fecal sampling”) OR AB,TI,IF(“Faecal sampling”) OR AB,TI,IF(“Stool sampling”) OR AB,TI,IF(“Stool collection”) OR SU(“Bacterial infections”) OR SU(“Viral infections”) OR SU(“Parasitic diseases”)		AB,TI,IF(“Child nutrition disorders”) OR AB,TI,IF(“Wasting syndrome”) OR AB,TI,IF(“Thinness”) OR AB,TI,IF(“Growth velocity”) OR SU(“Underweight”) OR SU(“Weight”) OR SU(“Physical growth”) OR SU(“Growth disorders”) OR SU(“Malnutrition”)
**Scopus**	TITLE-ABS-KEY(“matched”) OR TITLE-ABS-KEY(“trial”) OR TITLE-ABS-KEY(“RCT”) OR TITLE-ABS-KEY(“experiment”) OR TITLE-ABS-KEY(“intervention”) OR TITLE-ABS-KEY(“randomi*ed”) OR TITLE-ABS-KEY(“quasi-randomi*ed”) OR TITLE-ABS-KEY(“quasi-experimental”) OR TITLE-ABS-KEY(“pseudo-randomi*ed”)	TITLE-ABS-KEY(“Water”) OR TITLE-ABS-KEY(“Sanitation”) OR TITLE-ABS-KEY(“Hygiene”) OR TITLE-ABS-KEY(“Handwashing”) OR TITLE-ABS-KEY(“WSH”) OR TITLE-ABS-KEY(“Sanitary Drainage”) OR TITLE-ABS-KEY(“disinfect*”)	TITLE-ABS-KEY(“molecular source tracking”) OR TITLE-ABS-KEY(“microbial source tracking”) OR TITLE-ABS-KEY(“microbial transmission”) OR TITLE-ABS-KEY(“diarrh*eal pathogen”) OR TITLE-ABS-KEY(“f*ecal-oral”) OR TITLE-ABS-KEY(“enteric pathogen”) OR TITLE-ABS-KEY(“ruminant*”) OR TITLE-ABS-KEY(“avian*”) OR TITLE-ABS-KEY(“f*ecal”) OR TITLE-ABS-KEY (“f*eces”) OR TITLE-ABS-KEY (“f*ecally”)	TITLE-ABS-KEY(“Enteric infection”) OR TITLE-ABS-KEY(“Soil-transmitted helminth”) OR TITLE-ABS-KEY(“Protozoan”) OR TITLE-ABS-KEY(“Seroconversion”) OR TITLE-ABS-KEY(“F*ecal microbio*”) OR TITLE-ABS-KEY(“F*ecal biomarker”) OR TITLE-ABS-KEY(“Seroconversion”) OR TITLE-ABS-KEY(“Helminthiasis”) OR TITLE-ABS-KEY(“Bacterial infection”) OR TITLE-ABS-KEY(“Parasite infection”) OR TITLE-ABS-KEY(“Parasitic infection”) OR TITLE-ABS-KEY(“Helminth infection”) OR TITLE-ABS-KEY(“F*ecal sampling”)	TITLE-ABS-KEY(“Diarrh*”) OR TITLE-ABS-KEY(“Dysentery”) OR TITLE-ABS-KEY(“Intestinal infection”) OR TITLE-ABS-KEY(“Viral infection”) OR OR TITLE-ABS-KEY(“Stool sampling”) OR TITLE-ABS-KEY(“Stool collection”)	TITLE-ABS-KEY(“Child growth”) OR TITLE-ABS-KEY(“Growth faltering”) OR TITLE-ABS-KEY(“Child development”) OR TITLE-ABS-KEY(“Length-for-age”) OR TITLE-ABS-KEY(“Height-for-age”) OR TITLE-ABS-KEY(“Weight-for-age”) OR TITLE-ABS-KEY(“Head circumference”) OR TITLE-ABS-KEY(“Waist circumference”) OR TITLE-ABS-KEY(“Stunt*”) OR TITLE-ABS-KEY(“Wasting”) OR TITLE-ABS-KEY(“Wasted”) OR TITLE-ABS-KEY(“Linear growth”) OR TITLE-ABS-KEY(“Anthropometric measurement”) OR TITLE-ABS-KEY(“Maln*”) OR TITLE-ABS-KEY(“Undernourish*”) OR TITLE-ABS-KEY(“Undernutrition”) OR TITLE-ABS-KEY(“Underweight”) OR TITLE-ABS-KEY(“Growth Disorder”) OR TITLE-ABS-KEY(“Child nutrition disorder”) OR TITLE-ABS-KEY(“Wasting syndrome”) OR TITLE-ABS-KEY(“Thinness”) OR TITLE-ABS-KEY(“Growth velocity”)

Table S2. PRISMA Checklist

Topic	No.	Item	Location where item is reported
**TITLE**			
**Title**	1	Identify the report as a systematic review.	Page 1
**ABSTRACT**			
**Abstract**	2	See the PRISMA for Abstracts checklist below	
**INTRODUCTION**			
**Rationale**	3	Describe the rationale for the review in the context of existing knowledge.	Introduction, paragraph 1
**Objectives**	4	Provide an explicit statement of the objective(s) or question(s) the review addresses.	Introduction, paragraph 1
**METHODS**			
**Eligibility criteria**	5	Specify the inclusion and exclusion criteria for the review and how studies were grouped for the syntheses.	Methods, paragraph 1
**Information sources**	6	Specify all databases, registers, websites, organisations, reference lists and other sources searched or consulted to identify studies. Specify the date when each source was last searched or consulted.	Methods, paragraph 1
**Search strategy**	7	Present the full search strategies for all databases, registers and websites, including any filters and limits used.	Methods, paragraph 1, citing related article.
**Selection process**	8	Specify the methods used to decide whether a study met the inclusion criteria of the review, including how many reviewers screened each record and each report retrieved, whether they worked independently, and if applicable, details of automation tools used in the process.	Methods, paragraph 1, citing related article.
**Data collection process**	9	Specify the methods used to collect data from reports, including how many reviewers collected data from each report, whether they worked independently, any processes for obtaining or confirming data from study investigators, and if applicable, details of automation tools used in the process.	Methods, paragraph 1, citing related article.

Topic	No.	Item	Location where item is reported
**Data items**	10a	List and define all outcomes for which data were sought. Specify whether all results that were compatible with each outcome domain in each study were sought (e.g. for all measures, time points, analyses), and if not, the methods used to decide which results to collect.	Methods, paragraph 2
	10b	List and define all other variables for which data were sought (e.g. participant and intervention characteristics, funding sources). Describe any assumptions made about any missing or unclear information.	Methods, paragraph 1, citing related article.
**Study risk of bias assessment**	11	Specify the methods used to assess risk of bias in the included studies, including details of the tool(s) used, how many reviewers assessed each study and whether they worked independently, and if applicable, details of automation tools used in the process.	Methods, paragraph 1, Table S2
**Effect measures**	12	Specify for each outcome the effect measure(s) (e.g. risk ratio, mean difference) used in the synthesis or presentation of results.	Methods, paragraph 3
**Synthesis methods**	13a	Describe the processes used to decide which studies were eligible for each synthesis (e.g. tabulating the study intervention characteristics and comparing against the planned groups for each synthesis (item 5)).	Methods, paragraph 3
	13b	Describe any methods required to prepare the data for presentation or synthesis, such as handling of missing summary statistics, or data conversions.	Methods, paragraph 3
	13c	Describe any methods used to tabulate or visually display results of individual studies and syntheses.	Figure captions
	13d	Describe any methods used to synthesize results and provide a rationale for the choice(s). If meta-analysis was performed, describe the model(s), method(s) to identify the presence and extent of statistical heterogeneity, and software package(s) used.	Methods, paragraph 3
	13e	Describe any methods used to explore possible causes of heterogeneity among study results (e.g. subgroup analysis, meta-regression).	Methods, paragraphs 4,5
	13f	Describe any sensitivity analyses conducted to assess robustness of the synthesized results.	Methods, paragraph 5
**Reporting bias assessment**	14	Describe any methods used to assess risk of bias due to missing results in a synthesis (arising from reporting biases).	Not applicable
**Certainty assessment**	15	Describe any methods used to assess certainty (or confidence) in the body of evidence for an outcome.	Not applicable

Topic	No.	Item	Location where item is reported
**RESULTS**			
**Study selection**	16a	Describe the results of the search and selection process, from the number of records identified in the search to the number of studies included in the review, ideally using a flow diagram.	Figure S1
	16b	Cite studies that might appear to meet the inclusion criteria, but which were excluded, and explain why they were excluded.	Results, paragraph 1
**Study characteristics**	17	Cite each included study and present its characteristics.	Results, paragraph 1
**Risk of bias in studies**	18	Present assessments of risk of bias for each included study.	Table S2
**Results of individual studies**	19	For all outcomes, present, for each study: (a) summary statistics for each group (where appropriate) and (b) an effect estimate and its precision (e.g. confidence/credible interval), ideally using structured tables or plots.	Figures 1 ,2 3, S2-S20
**Results of syntheses**	20a	For each synthesis, briefly summarise the characteristics and risk of bias among contributing studies.	Not applicable
	20b	Present results of all statistical syntheses conducted. If meta-analysis was done, present for each the summary estimate and its precision (e.g. confidence/credible interval) and measures of statistical heterogeneity. If comparing groups, describe the direction of the effect.	Figures1 ,2 3, S2-S20
	20c	Present results of all investigations of possible causes of heterogeneity among study results.	Results, Subgroup analyses section, and Figures S9-S14
	20d	Present results of all sensitivity analyses conducted to assess the robustness of the synthesized results.	Results, Sensitivity analyses section, and Figures S15-S20
**Reporting biases**	21	Present assessments of risk of bias due to missing results (arising from reporting biases) for each synthesis assessed.	Not applicable
**Certainty of evidence**	22	Present assessments of certainty (or confidence) in the body of evidence for each outcome assessed.	Figures 1 ,2 3, S2-S20
**DISCUSSION**			
**Discussion**	23a	Provide a general interpretation of the results in the context of other evidence.	Discussion, paragraph 1
	23b	Discuss any limitations of the evidence included in the review.	Discussion, paragraphs 2, 7

Topic	No.	Item	Location where item is reported
	23c	Discuss any limitations of the review processes used.	Discussion, paragraph 2, cited related article
	23d	Discuss implications of the results for practice, policy, and future research.	Discussion, paragraph 5, 6, 8
**OTHER INFORMATION**			
**Registration and protocol**	24a	Provide registration information for the review, including register name and registration number, or state that the review was not registered.	https://osf.io/8sgzn/
	24b	Indicate where the review protocol can be accessed, or state that a protocol was not prepared.	https://osf.io/8sgzn/
	24c	Describe and explain any amendments to information provided at registration or in the protocol.	Not applicable
**Support**	25	Describe sources of financial or non-financial support for the review, and the role of the funders or sponsors in the review.	Abstract, Funding
**Competing interests**	26	Declare any competing interests of review authors.	Not applicable
**Availability of data, code and other materials**	27	Report which of the following are publicly available and where they can be found: template data collection forms; data extracted from included studies; data used for all analyses; analytic code; any other materials used in the review.	https://github.com/amertens/wash-ipd

PRISMA FOR ABSTRACTS

Topic	No.	Item	Reported?
**TITLE**			
**Title**	1	Identify the report as a systematic review.	Yes
**BACKGROUND**			
**Objectives**	2	Provide an explicit statement of the main objective(s) or question(s) the review addresses.	Yes
**METHODS**			
**Eligibility criteria**	3	Specify the inclusion and exclusion criteria for the review.	Yes

Topic	No.	Item	Reported?
**Information sources**	4	Specify the information sources (e.g. databases, registers) used to identify studies and the date when each was last searched.	No
**Risk of bias**	5	Specify the methods used to assess risk of bias in the included studies.	Yes
**Synthesis of results**	6	Specify the methods used to present and synthesize results.	Yes
**RESULTS**			
**Included studies**	7	Give the total number of included studies and participants and summarise relevant characteristics of studies.	Yes
**Synthesis of results**	8	Present results for main outcomes, preferably indicating the number of included studies and participants for each. If meta-analysis was done, report the summary estimate and confidence/credible interval. If comparing groups, indicate the direction of the effect (i.e. which group is favoured).	Yes
**DISCUSSION**			
**Limitations of evidence**	9	Provide a brief summary of the limitations of the evidence included in the review (e.g. study risk of bias, inconsistency and imprecision).	Yes
**Interpretation**	10	Provide a general interpretation of the results and important implications.	Yes
**OTHER**			
**Funding**	11	Specify the primary source of funding for the review.	Yes
**Registration**	12	Provide the register name and registration number.	Yes

Table S3. Risk of bias based on modified Newcastle-Ottawa scale

Stars are given for low risk of bias in each category, up to a total of nine stars. Scoring details are in the footnotes.

Study	Reference	Selection bias	Response bias	Follow-up bias	Misclassification bias	Outcome assessment	Outcome measurement	Bias in analysis	Total
		Is there evidence of selection bias, which refers to systematic differences between baseline characteristics of the groups that are compared?[a]	Is there evidence of response bias?[b]	Is there evidence of bias due to missing follow-up data?[c]	Is there risk of households not receiving the intervention being misclassified as having received it, or vice versa?[d]	Is there evidence of bias arising from how the outcome was assessed?[e]	Is there evidence of ascertainment bias?[f]	Is there evidence that analysis was not appropriately adjusted for clustering and/or confounding, if appropriate?[g]	Total number of stars (x/9 possible stars).
Total Sanitation Campaign (Odisha)		*	possible (no blinding)	possible (86% of possible weeks are reported weeks)	* household-level interventions	caregiver recall for diarrhoea, direct measurement for growth, and laboratory detection for pathogen-specific infections	possible (no blinding of assessor or person under study)	** adjusted for clustering	4
WASH Benefits Bangladesh		*	* included negative control outcome, participants not blinded	* 94% complete FU	* household-level interventions	caregiver recall for diarrhoea, direct measurement for growth, and laboratory detection for pathogen-specific infections	possible, data collectors not blinded (statistical analysis blinded)	**	6
WASH Benefits Kenya		*	* included negative control outcome, participants not blinded	* <1% loss to FU	* household-level interventions	caregiver recall for diarrhoea, direct measurement for growth, and laboratory detection for pathogen-specific infections	possible, data collectors not blinded (statistical analysis blinded)	**	5

Study	Reference	Selection bias	Response bias	Follow-up bias	Misclassification bias	Outcome assessment	Outcome measurement	Bias in analysis	Total
Gram Vikas		selection bias is possible, as the study is not randomized and there are some baseline differences between intervention and control group	* no, assessed through negative control outcome	substantial loss to FU	* household-level interventions	caregiver recall for diarrhoea, direct measurement for growth, and laboratory detection for pathogen-specific infections	possible (no blinding of assessor or person under study)	**	4
MapSan		selection bias is possible, as the study is not randomized, but intervention and control groups were mostly balanced at baseline. Control households were more likely to have covered floors and higher quality walls and intervention groups had more people per household.	possible (no blinding)	substantial loss to FU	* household-level interventions	caregiver recall for diarrhoea, direct measurement for growth, and laboratory detection for pathogen-specific infections	possible (no blinding of assessor or person under study)	**	3

[a] RCTs receive 1 star, unless evidence of selection bias (e.g. randomisation procedures not followed). Meaningful differences between groups at baseline in RCTs receive 0 stars. Rates of declining to participate >10% receive 0 stars. Non- or quasi-randomised studies receive 0 stars.

[b] If intervention recipient was not blinded to intervention status, 0 stars.

[c] <10% receives 1 star, greater than or equal to 10% receives 0 stars.

[d] Interventions delivered at the household/individual level receive 1 star. Interventions delivered at the community level that missed a substantial, i.e. greater than or equal to 10%, proportion of the target population receive 0 stars, including when there is insufficient information to verify whether this is the case. Interventions with substantial risk of contamination (control households receiving intervention) receive 0 stars.

[e] Parent / person recall (=0 stars). Fieldworker assessed (=1 star). Physician/microbiologically assessed (=2 stars)

[f] If outcome measurement staff were not blinded to intervention status, 0 stars.

[g] Scoring is based on losing stars (max. 2). Individual RCTs with baseline balance on covariates are unlikely to require adjustment (=2 stars). Cluster-RCTs and non-randomised trials may require adjustment for clustering (-1 star if not done). RCTs or cRCTs may require adjustment for covariates, with justification (-1 star if not done). Non-randomised studies require adjustment for covariates (-1 star if not done), but also adequate justification for covariate selection (-1 star if not included), and there can be too few or too many covariates.

Table S4. Institution Review Board (IRB) Protocols and Trial Registrations

Study	IRB (Protocol Number) #1	IRB (Protocol Number) #2	IRB (Protocol Number) #3	Clinical trial registration
Mapsan	Comité Nacional de Bioética para a Saúde (CNBS), Ministério da Saúde (333/CNBS/14)	Research Ethics Committee of the London School of Hygiene & Tropical Medicine (reference # 8345)	Institutional Review Board of the Georgia Institute of Technology (protocol # H15160)	NCT02362932
Gram Vikas	London School of Hygiene and Tropical Medicine, London, U.K (No. 9071)	Kalinga Institute of Medical Sciences of KIIT University, Bhubaneswar, India (KIMS/KIIT/IEC/053/2015) ethics committees	Emory University IRB (IRB00079717)	NCT02441699
Total Sanitation Campaign (Odisha)	LSHTM Ethics committee	The Xavier Institute of Management	Bhubaneswar and the Asian Institute of Public Health	NCT01214785
WASH Benefits Bangladesh	Berkeley: 2011-09-3652	Stanford: eProtocol #: 25863 IRB Number: 351	Icddr'b: PR 11063	NCT01590095
WASH Benefits Kenya	Berkeley: 2011-09-3654	Stanford: Protocol ID: 23310 IRB Number: 349 (Panel: 2)	KEMRI: Protocol 2271	NCT01590095
